# The Involvement of the hsa_circ_0088494-miR-876-3p-CTNNB1/CCND1 Axis in Carcinogenesis and Progression of Papillary Thyroid Carcinoma

**DOI:** 10.3389/fcell.2020.605940

**Published:** 2020-12-09

**Authors:** Weiyang Lou, Bisha Ding, Jiannan Wang, Yongfang Xu

**Affiliations:** ^1^Department of Breast Surgery, The First Affiliated Hospital, College of Medicine, Zhejiang University, Hangzhou, China; ^2^Key Laboratory of Combined Multi-Organ Transplantation, Division of Hepatobiliary and Pancreatic Surgery, Program of Innovative Cancer Therapeutics, Department of Surgery, First Affiliated Hospital, College of Medicine, Zhejiang University, Ministry of Public Health, Hangzhou, China; ^3^Key Laboratory of Organ Transplantation, Zhejiang University, Hangzhou, China; ^4^Department of Respiratory Medicine, Thoracic Disease Center, The First Affiliated Hospital, College of Medicine, Zhejiang University, Hangzhou, China

**Keywords:** circular RNA, hsa_circ_0088494, microRNA, miR-876-3p, CTNNB1, CCND1, papillary thyroid carcinoma

## Abstract

Recently, growing studies have demonstrated that circular RNAs (circRNAs) function as critical players in multiple human tumors, including papillary thyroid carcinoma (PTC). However, the expression and underlying potential mechanism of circRNAs in PTC are still not fully elucidated. In this study, 14 candidate differentially expressed circRNAs (DECs) between normal thyroid tissues and benign thyroid tissues or PTC were first screened using the GSE93522 dataset by the GEO2R online tool. Then, the structural loop graphs of these 14 circRNAs were obtained through the CSCD database. After performing miRNA co-prediction by combination of CSCD and CRI databases, a potential circRNA-miRNA sub-network, consisting of 9 circRNAs and 21 miRNAs, was successfully constructed. Subsequently, the expression and prognostic values of these miRNAs were further determined by starBase, and two miRNAs, namely, miR-605-5p and miR-876-3p, were identified as key miRNAs in PTC. Then, their downstream target genes were predicted by the miRNet database. CTNNB1 and CCND1 were found to be two most potential targets of miR-876-3p by combination of multiple *in silico* analyses, including protein–protein interaction (PPI), hub gene screening, correlation analysis, and expression analysis. Conclusively, we established a key hsa_circ_0088494-miR-876-3p-CTNNB1/CCND1 axis linked to carcinogenesis and progression of PTC, which may provide promising therapeutic targets in treating PTC in the future.

## Introduction

Papillary thyroid carcinoma (PTC) is one of the most prevalent malignant tumors all over the world, which accounts for more than 80% of thyroid cancers (Lim et al., 2017). Among all malignant cancer types, PTC’s prognosis is generally good and the 10-year survival rate of PTC has reached about 90% due to its low rate of tumor growth and high degree of tumor differentiation (Gui et al., 2020). However, the total survival rate of PTC patients with some aggressive features, such as lymph node and distant metastatic potentials, is still unsatisfactory (Xue et al., 2020). Thus, it is of great significance to uncover the underlying molecular mechanism of PTC carcinogenesis and progression, which may provide novel therapeutic targets for PTC treatment.

Circular RNAs (circRNAs) are a class of endogenous non-coding RNAs (ncRNAs) with covalently closed loops (Qu et al., 2015). Increasing evidences have showed that deregulation of circRNAs widely appears in a series of human diseases and dysregulated circRNAs play key roles in pathogenesis of these diseases, especially cancers (Ding et al., 2020; Guo et al., 2020; Yang T. et al., 2020). In PTC, several circRNAs have been validated to be involved in development of PTC. For example, Wang et al. (2018) suggested that circ-ITCH inhibited progression of PTC through the miR-22-3p/CBL/beta-catenin pathway; Bi et al. (2018) showed that circRNA_102171 promoted the progression of PTC by regulating CTNNB1P1-dependent activation of the beta-catenin pathway; Liu et al. (2018) also indicated that hsa_circ_0060060 enhanced cisplatin resistance of human thyroid carcinoma cells by autophagy regulation. However, the current knowledge regarding circRNAs in PTC is still not enough, and the expression profile and detailed mechanism in PTC need to be further explored.

In this study, we first identified differentially expressed circRNAs (DECs) between normal thyroid tissues and benign thyroid tissues (normal vs. benign comparison) or PTC (normal vs. PTC comparison). Those identified DECs that were only significantly upregulated or downregulated in normal vs. PTC comparison were screened out and were regarded as candidate circRNAs in PTC. Next, a potential hsa_circRNA/miRNA/mRNA regulatory axis linked to PTC carcinogenesis and progression was established through a series of *in silico* analyses, including miRNA co-prediction, expression analysis, survival analysis, target gene prediction, protein–protein interaction (PPI) network analysis, pathway enrichment analysis, hub gene identification, and miRNA–hub gene expression correlation. The finding from this study may provide key clues for seeking and developing therapeutic targets in treating PTC.

## Results

### Selection of Potential circRNAs in PTC

**FIGURE 1:**
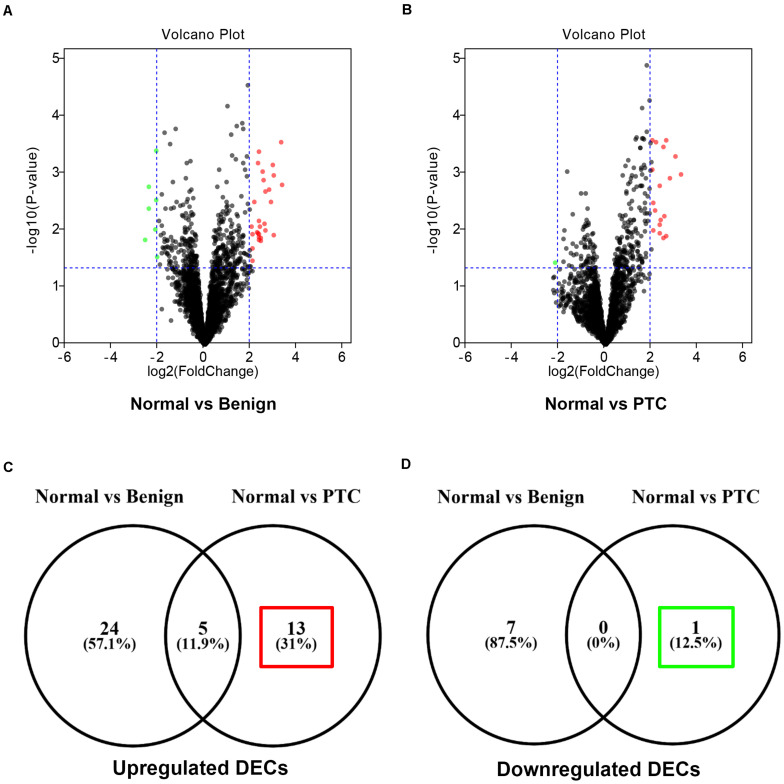
Identification of candidate circRNAs associated with progression of papillary thyroid carcinoma (PTC). **(A)** The volcano plot of differentially expressed circRNAs (DECs) between 6 benign thyroid lesions and 6 normal thyroid tissues. **(B)** The volcano plot of differentially expressed circRNAs (DECs) between 6 PTC and 6 normal thyroid tissues. The red dots and green dots respectively represent upregulated DECs and downregulated DECs with significance (*P* < 0.05 and |log_2_FC| > 1). The black dots are those DECs without significance. **(C)** The interaction analysis for upregulated DECs in both compared groups. **(D)** The interaction analysis for downregulated DECs in both compared groups.

**FIGURE 2:**
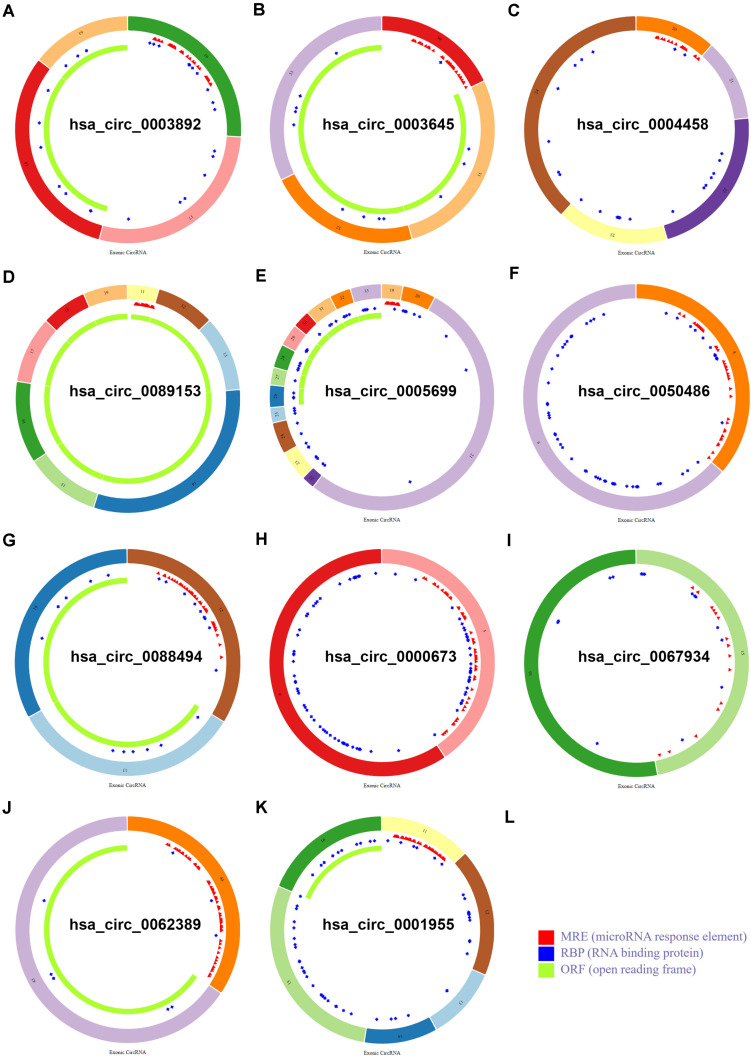
Structural patterns of the 11 circRNAs in the CSCD database. **(A)** The structural pattern of hsa_circ_0003892. **(B)** The structural pattern of hsa_circ_0003645. **(C)** The structural pattern of hsa_circ_0004458. **(D)** The structural pattern of hsa_circ_0089153. **(E)** The structural pattern of hsa_circ_0005699. **(F)** The structural pattern of hsa_circ_0050486. **(G)** The structural pattern of hsa_circ_0088494. **(H)** The structural pattern of hsa_circ_0000673. **(I)** The structural pattern of hsa_circ_0067934. **(J)** The structural pattern of hsa_circ_0062389. **(K)** The structural pattern of hsa_circ_0001955. **(L)** The representation of MRE, RBP, and ORF.

To find some potential circRNAs associated with carcinogenesis and progression of PTC, the GSE93522 dataset from the GEO database was finally used by a series of criteria selection, after performing differential expression analysis between normal thyroid tissues and benign thyroid tissues or PTC using the GEO2R tool. As shown in Figure 1A and Table 1, 29 and 7 circRNAs were significantly upregulated and downregulated in benign thyroid tissues when compared with normal thyroid tissues, respectively. Moreover, a total of 19 significant DECs, consisting of 18 upregulated and 1 downregulated DECs, were discovered in normal vs. PTC comparison (Figure 1B and Table 2). In order to improve the analytic accuracy, integration analysis between normal vs. PTC and normal vs. benign comparisons was carried out. We screened out those circRNAs that were only significantly differentially expressed in normal vs. PTC comparison (not in normal vs. benign comparison). Consequently, as presented in Figures 1C,D, we found that 13 upregulated (red box) and 1 downregulated (green box) circRNAs were identified. These 14 candidate circRNAs and their detailed information, including circBase ID, parental gene, and genome location, are listed in Table 3. To further understand the 14 circRNAs, the structural loop graphs were described by the CSCD database. Finally, 11 of 14 candidate circRNAs were accessed (Figure 2), including hsa_circ_0003892 (Figure 2A), hsa_circ_0003645 (Figure 2B), hsa_circ_0004458 (Figure 2C), hsa_circ_0089153 (Figure 2D), hsa_circ_0005699 (Figure 2E), hsa_circ_0050486 (Figure 2F), hsa_circ_0088494 (Figure 2G), hsa_circ_0000673 (Figure 2H), hsa_circ_0067934 (Figure 2I), hsa_circ_0062389 (Figure 2J), and hsa_circ_0001955 (Figure 2K). All of them were upregulated circRNAs in PTC. The other 3 circRNAs were not available in the CSCD database. Therefore, the 11 circRNAs were regarded as the final candidate circRNAs in PTC.

**TABLE 1:** The differentially expressed circRNAs (DECs) with significance (|log_2_FC| > 2 and *P* < 0.05) between benign thyroid lesions and normal thyroid tissues.

circRNA name	*P*-value	*t*	B	log_2_FC
hsa_circRNA_104342	0.001564	4.023580	–0.9063	3.321699
hsa_circRNA_101868	0.000277	4.994162	0.6605	3.292771
hsa_circRNA_101765	0.001076	4.228282	–0.5652	2.976937
hsa_circRNA_103809	0.011945	2.939085	–2.7627	2.971844
hsa_circRNA_102704	0.000705	4.462817	–0.1809	2.956482
hsa_circRNA_001350	0.003145	3.646917	–1.5449	2.847959
hsa_circRNA_104652	0.001911	3.914755	–1.0895	2.769816
hsa_circRNA_101957	0.009671	3.050815	–2.5708	2.614934
hsa_circRNA_104700	0.002042	3.879060	–1.1498	2.601478
hsa_circRNA_100160	0.007385	3.193435	–2.3250	2.596365
hsa_circRNA_100181	2.95E-06	7.991081	4.5325	2.582072
hsa_circRNA_101764	0.001282	4.131767	–0.7254	2.520950
hsa_circRNA_104916	0.000894	4.330489	–0.3968	2.488498
hsa_circRNA_100660	0.015220	2.810679	–2.9823	2.432437
hsa_circRNA_100146	0.013228	2.885056	–2.8553	2.424340
hsa_circRNA_102631	0.008427	3.123584	–2.4455	2.395230
hsa_circRNA_101958	0.012940	2.896726	–2.8353	2.346514
hsa_circRNA_100883	0.014417	2.839446	–2.9332	2.344302
hsa_circRNA_000963	0.011232	2.971634	–2.7069	2.337819
hsa_circRNA_101217	0.000400	4.783452	0.3321	2.326712
hsa_circRNA_103408	0.006576	3.254812	–2.2192	2.322926
hsa_circRNA_101934	0.000643	4.514227	–0.0976	2.309377
hsa_circRNA_103948	0.011036	2.980939	–2.6909	2.297155
hsa_circRNA_101287	0.010437	3.010505	–2.6401	2.240166
hsa_circRNA_101408	0.003128	3.649905	–1.5398	2.122885
hsa_circRNA_101248	0.020534	2.651386	–3.2524	2.079060
hsa_circRNA_102509	0.033992	2.380562	–3.7027	2.071983
hsa_circRNA_104803	0.011372	2.965096	–2.7181	2.061757
hsa_circRNA_001264	0.008409	3.124738	–2.4435	2.031883
hsa_circRNA_400012	0.028353	–2.478540	–3.5414	–2.054310
hsa_circRNA_100332	0.002891	–3.692060	–1.4678	–2.104710
hsa_circRNA_102051	0.000385	–4.804180	0.3647	–2.106300
hsa_circRNA_102049	0.009392	–3.066280	–2.5441	–2.135810
hsa_circRNA_102485	0.003997	–3.519050	–1.7642	–2.406910
hsa_circRNA_000684	0.001655	–3.992890	–0.9578	–2.421530
hsa_circRNA_103944	0.014229	–2.846400	–2.9213	–2.562840

**TABLE 2:** The differentially expressed circRNAs (DECs) with significance (|log_2_FC| > 2 and *P* < 0.05) between papillary thyroid carcinoma (PTC) and normal thyroid tissues.

circRNA name	*P*-value	*t*	B	log_2_FC
hsa_circRNA_103809	0.001022	4.239200	–0.5241	3.267325
hsa_circRNA_100146	0.000499	4.635729	0.1277	3.009379
hsa_circRNA_101287	0.00118	4.160994	–0.6551	2.773775
hsa_circRNA_102451	0.012167	2.922315	–2.7877	2.633623
hsa_circRNA_104700	0.000257	5.012807	0.7264	2.606228
hsa_circRNA_400024	0.005424	3.347087	–2.0510	2.557434
hsa_circRNA_101748	0.013351	2.873444	–2.8719	2.488262
hsa_circRNA_104566	0.000334	4.863195	0.4915	2.485302
hsa_circRNA_104940	0.006433	3.257263	–2.2071	2.389848
hsa_circRNA_104387	0.001596	3.997283	–0.9315	2.342137
hsa_circRNA_101744	0.011028	2.974014	–2.6984	2.328425
hsa_circRNA_102513	0.007832	3.153883	–2.3867	2.327728
hsa_circRNA_101408	0.000271	4.981205	0.6770	2.188620
hsa_circRNA_104917	0.004330	3.465786	–1.8449	2.133435
hsa_circRNA_101707	0.009942	3.028509	–2.6041	2.078851
hsa_circRNA_103510	0.003177	3.62952	–1.5615	2.062743
hsa_circRNA_103164	0.000258	5.010599	0.7229	2.001435
hsa_circRNA_101555	0.000849	4.340494	–0.3556	2.000987
hsa_circRNA_000104	0.036349	−2.340130	–3.7705	−2.177480

**TABLE 3:** Fourteen candidate circRNAs associated with progression of papillary thyroid carcinoma (PTC) and their corresponding information.

circRNA name	circBase ID	Parental gene	Location
hsa_circRNA_102451	hsa_circ_0003892	*LDLR*	chr19:11230767–11238761
hsa_circRNA_400024	hsa_circ_0092363	*DDX11*	chr12:31241497–31241717
hsa_circRNA_101748	hsa_circ_0003645	*C16orf62*	chr16:19656207–19663412
hsa_circRNA_104566	hsa_circ_0004458	*PSD3*	chr8:18656804–18662408
hsa_circRNA_104940	hsa_circ_0089153	*NUP214*	chr9:134011326–134022971
hsa_circRNA_104387	hsa_circ_0080425	*WBSCR17*	chr7:70880874–70886091
hsa_circRNA_101744	hsa_circ_0005699	*C16orf62*	chr16:19627435–19663412
hsa_circRNA_102513	hsa_circ_0050486	*GPI*	chr19:34868407–34868786
hsa_circRNA_104917	hsa_circ_0088494	*NEK6*	chr9:127083737–127089724
hsa_circRNA_101707	hsa_circ_0000673	*RSL1D1*	chr16:11940357–11940700
hsa_circRNA_103510	hsa_circ_0067934	*PRKCI*	chr3:170013698–170015181
hsa_circRNA_103164	hsa_circ_0062389	*PI4KA*	chr22:21158587–21159453
hsa_circRNA_101555	hsa_circ_0001955	*CSNK1G1*	chr15:64495280–64508912
hsa_circRNA_000104	hsa_circ_0000266	*FAM53B*	chr10:126336614–126337010

### Prediction and Analysis of Binding miRNAs of circRNAs in PTC

**FIGURE 3:**
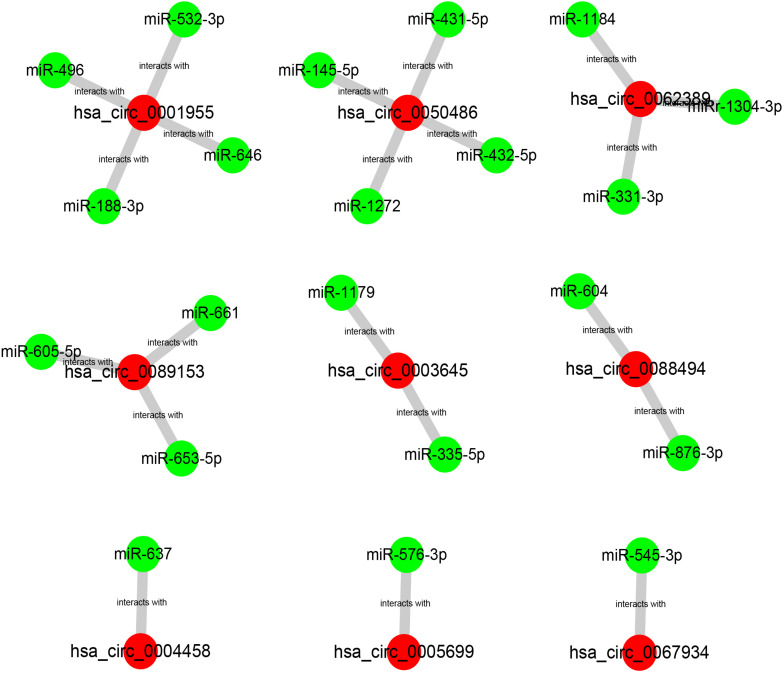
The potential binding miRNAs of 9 circRNAs predicted by CSCD and circular RNA interactome (CRI) databases.

**FIGURE 4:**
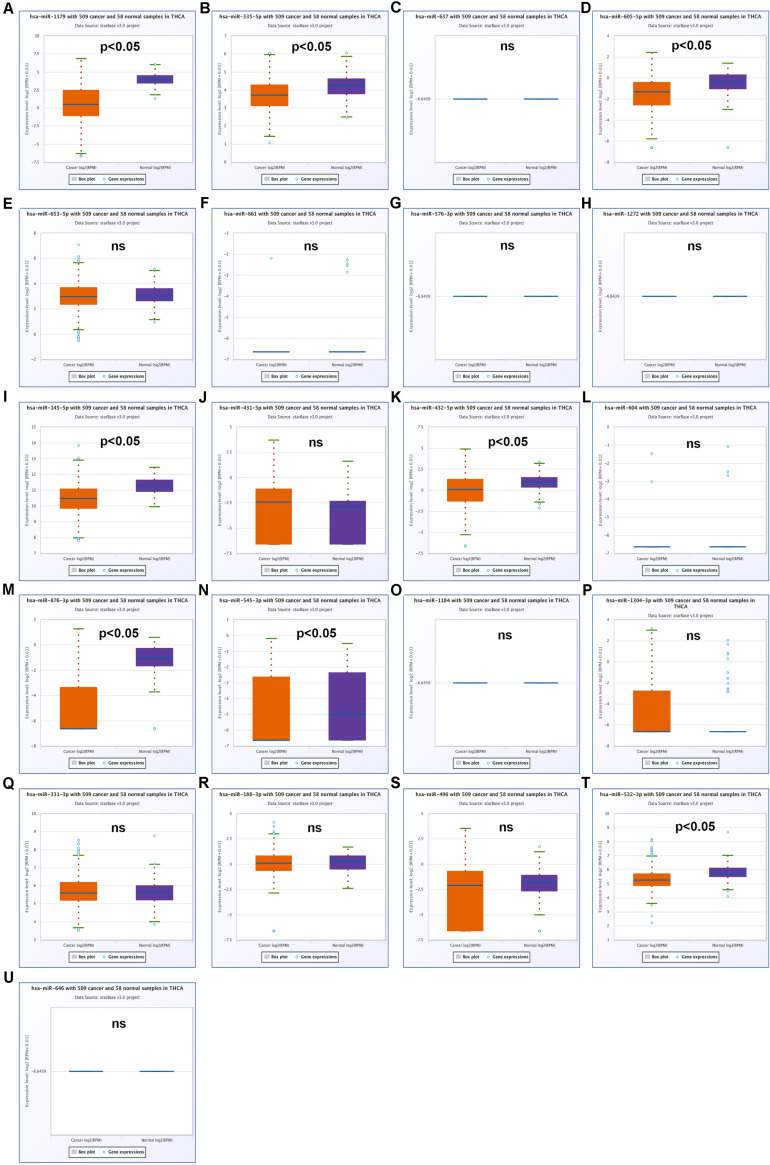
The expression levels of 21 predicted miRNAs that potentially bind to 9 candidate circRNAs in thyroid carcinoma. **(A)** The expression of miR-1179 in thyroid carcinoma. **(B)** The expression of miR-335-5p in thyroid carcinoma. **(C)** The expression of miR-637 in thyroid carcinoma. **(D)** The expression of miR-605-5p in thyroid carcinoma. **(E)** The expression of miR-653-5p in thyroid carcinoma. **(F)** The expression of miR-661 in thyroid carcinoma. **(G)** The expression of miR-576-3p in thyroid carcinoma. **(H)** The expression of miR-1272 in thyroid carcinoma. **(I)** The expression of miR-145-5p in thyroid carcinoma. **(J)** The expression of miR-431-5p in thyroid carcinoma. **(K)** The expression of miR-432-5p in thyroid carcinoma. **(L)** The expression of miR-604 in thyroid carcinoma. **(M)** The expression of miR-876-3p in thyroid carcinoma. **(N)** The expression of miR-545-3p in thyroid carcinoma. **(O)** The expression of miR-1184 in thyroid carcinoma. **(P)** The expression of miR-1304-3p in thyroid carcinoma. **(Q)** The expression of miR-331-3p in thyroid carcinoma. **(R)** The expression of miR-188-3p in thyroid carcinoma. **(S)** The expression of miR-496 in thyroid carcinoma. **(T)** The expression of miR-532-3p in thyroid carcinoma. **(U)** The expression of miR-646 in thyroid carcinoma. “*p* < 0.05” represents significant difference, and “ns” represents no significance.

**FIGURE 5:**
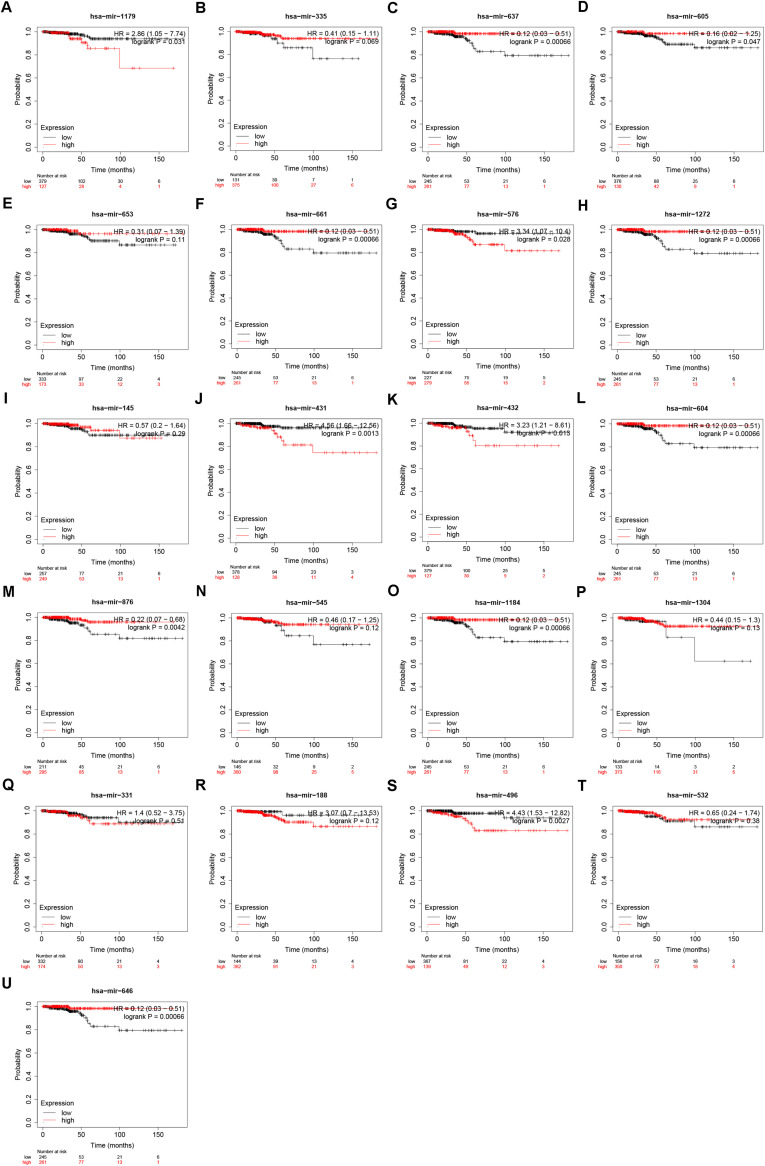
The prognostic values of 21 predicted miRNAs that potentially bind to 9 candidate circRNAs in thyroid carcinoma. **(A)** The prognostic value of miR-1179 in thyroid carcinoma. **(B)** The prognostic value of miR-335-5p in thyroid carcinoma. **(C)** The prognostic value of miR-637 in thyroid carcinoma. **(D)** The prognostic value of miR-605-5p in thyroid carcinoma. **(E)** The prognostic value of miR-653-5p in thyroid carcinoma. **(F)** The prognostic value of miR-661 in thyroid carcinoma. **(G)** The prognostic value of miR-576-3p in thyroid carcinoma. **(H)** The prognostic value of miR-1272 in thyroid carcinoma. **(I)** The prognostic value of miR-145-5p in thyroid carcinoma. **(J)** The prognostic value of miR-431-5p in thyroid carcinoma. **(K)** The prognostic value of miR-432-5p in thyroid carcinoma. **(L)** The prognostic value of miR-604 in thyroid carcinoma. **(M)** The prognostic value of miR-876-3p in thyroid carcinoma. **(N)** The prognostic value of miR-545-3p in thyroid carcinoma. **(O)** The prognostic value of miR-1184 in thyroid carcinoma. **(P)** The prognostic value of miR-1304-3p in thyroid carcinoma. **(Q)** The prognostic value of miR-331-3p in thyroid carcinoma. **(R)** The prognostic value of miR-188-3p in thyroid carcinoma. **(S)** The prognostic value of miR-496 in thyroid carcinoma. **(T)** The prognostic value of miR-532-3p in thyroid carcinoma. **(U)** The prognostic value of miR-646 in thyroid carcinoma. “logrank *P* < 0.05” represents significant difference.

**FIGURE 6:**
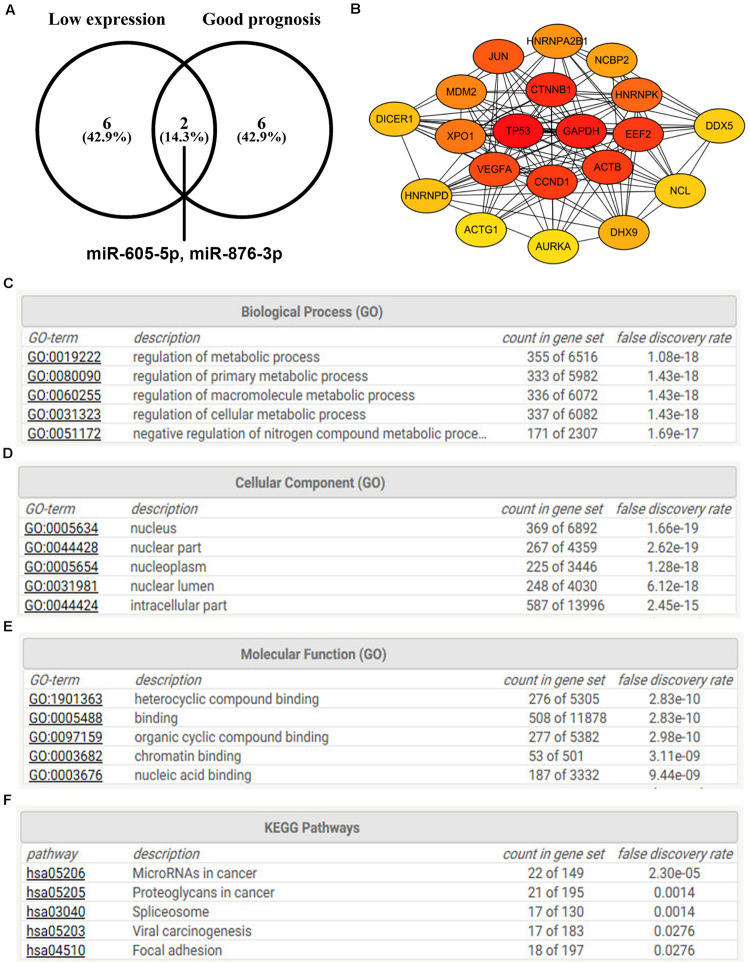
The protein–protein interaction (PPI), Gene Ontology (GO) functional annotation, and KEGG pathway enrichment analyses for target genes of miR-605-5p and miR-876-3p. **(A)** Identification of miR-605-5p and miR-876-3p as two most potential binding miRNAs of candidate circRNAs. **(B)** The PPI analysis for target genes of miR-605-5p and miR-876-3p. **(C)** The top 5 GO biological process items for target genes of miR-605-5p and miR-876-3p. **(D)** The top 5 GO cellular component items for target genes of miR-605-5p and miR-876-3p. **(E)** The top 5 GO molecular function items for target genes of miR-605-5p and miR-876-3p. **(F)** The top 5 KEGG pathways for target genes of miR-605-5p and miR-876-3p.

Our team and other groups have previously documented that circRNAs can act as miRNA sponges. Thus, we predicted the binding miRNAs of 11 candidate circRNAs. Two online prediction databases, containing CSCD and CRI, were employed to predict binding miRNAs of circRNAs (data were not shown). Only these miRNAs that were commonly appeared in both CSCD miRNA set and CRI miRNA set were selected for the following investigation, and these miRNAs were considered as candidate miRNAs. Finally, 9 of 11 circRNAs had candidate miRNAs. To better visualization, a potential circRNA-miRNA network, consisting of 9 circRNAs and 21 miRNAs, was established as presented in Figure 3. Based on the ceRNA hypothesis, the binding miRNAs of upregulated circRNAs should play opposite effects of miRNAs in PTC. Subsequently, the expression of 21 miRNAs in thyroid carcinoma was analyzed using starBase (Figure 4). The result suggested that 8 miRNAs (miR-1179, miR-335-5p, miR-605-5p, miR-145-5p, miR-432-5p, miR-876-3p, miR-545-3p, and miR-532-3p) were significantly downregulated in thyroid carcinoma compared with normal thyroid tissues. Moreover, the prognostic values of these 21 miRNAs in thyroid carcinoma were also assessed through the Kaplan–Meier plotter (Figure 5). Among the 21 miRNAs, only thyroid carcinoma patients with high expression of 8 miRNAs (miR-637, miR-605-5p, miR-661, miR-1272, miR-604, miR-876-3p, miR-1184, and miR-646) had favorable prognosis. As shown in Figure 6A, among these miRNAs, only two miRNAs (miR-605-5p and miR-876-3p) were significantly downregulated in thyroid carcinoma and were positively correlated with survival time of patients with thyroid carcinoma. Collectively, miR-605-5p and miR-876-3p might be two most potential miRNAs of candidate circRNAs in PTC.

### Prediction and Analysis for Target Genes of miRNAs in PTC

Next, the miRNet database was used to predict downstream target genes of miR-605-5p and miR-876-3p, and 687 targets were finally found as listed in Supplementary Table S1. To better understand the interaction relationship among all these targets, PPI network analysis was performed with the help of the STRING database. As presented in Supplementary Table S2, 3459 PPI pairs were obtained. According to node degree, the top 20 hub genes (TP53, GAPDH, CTNNB1, ACTB, EEF2, CCND1, VEGFA, JUN, HNRNPK, XPO1, MDM2, HNRNPA2B1, NCBP2, DHX9, DICER1, HNRNPD, NCL, DDX5, YBX1, and PCBP2) were screened, with TP53 being the highest node degree, which was 144 (Figure 6B). Then, GO functional annotation for the target genes of miR-605-5p and miR-876-3p was also conducted. As shown in Figures 6C–E, the enriched GO items for these target genes included regulation of the metabolic process, regulation of the primary metabolic process, and regulation of the macromolecule metabolic process in the biological process (BP) category; nucleus, nuclear part, and nucleoplasm in the cellular component (CC) category; and heterocyclic compound binding, binding, and organic cyclic compound in the molecular function (MF) category. Furthermore, KEGG pathway enrichment analysis was also conducted. The top 5 enriched KEGG pathways were microRNAs in cancer, proteoglycans in cancer, spliceosome, viral carcinogenesis, and focal adhesion (Figure 6F).

### Construction of a Potential hsa_circ_0088494-miR-876-3p-CTNNB1/CCND1 Axis in PTC

**FIGURE 7:**
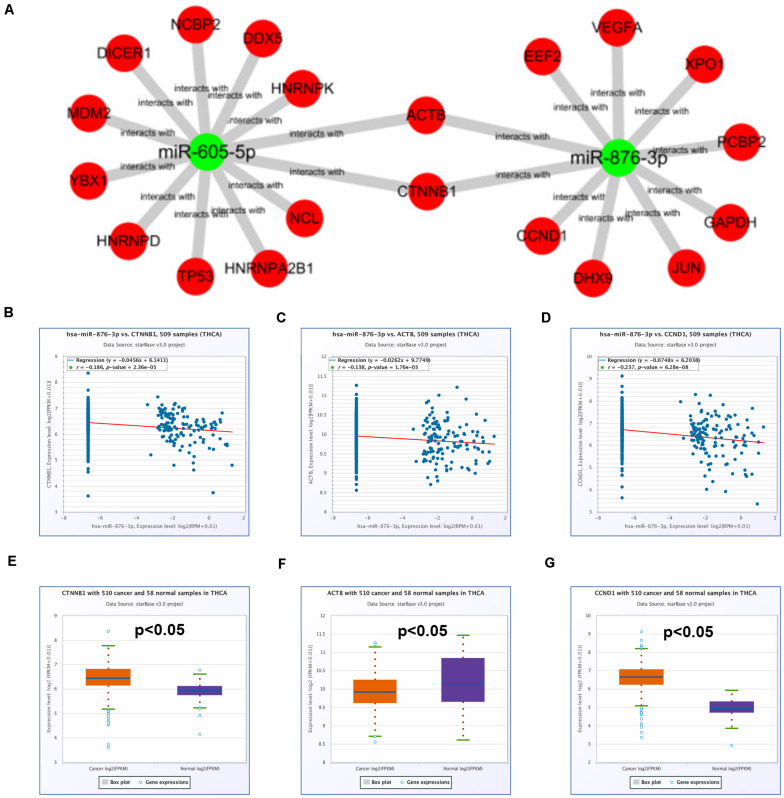
Identification of potential target genes of miR-605-5p and miR-876-3p in thyroid carcinoma. **(A)** The miRNA–hub gene network established by Cytoscape software. **(B)** The expression correlation of miR-876-3p with CTNNB1 in thyroid carcinoma. **(C)** The expression correlation of miR-876-3p with ACTB in thyroid carcinoma. **(D)** The expression correlation of miR-876-3p with CCND1 in thyroid carcinoma. **(E)** The mRNA expression level of CTNNB1 in thyroid carcinoma. **(F)** The mRNA expression level of ACTB in thyroid carcinoma. **(G)** The mRNA expression level of CCND1 in thyroid carcinoma. “*p* < 0.05” was considered as statistically significant.

**FIGURE 8:**
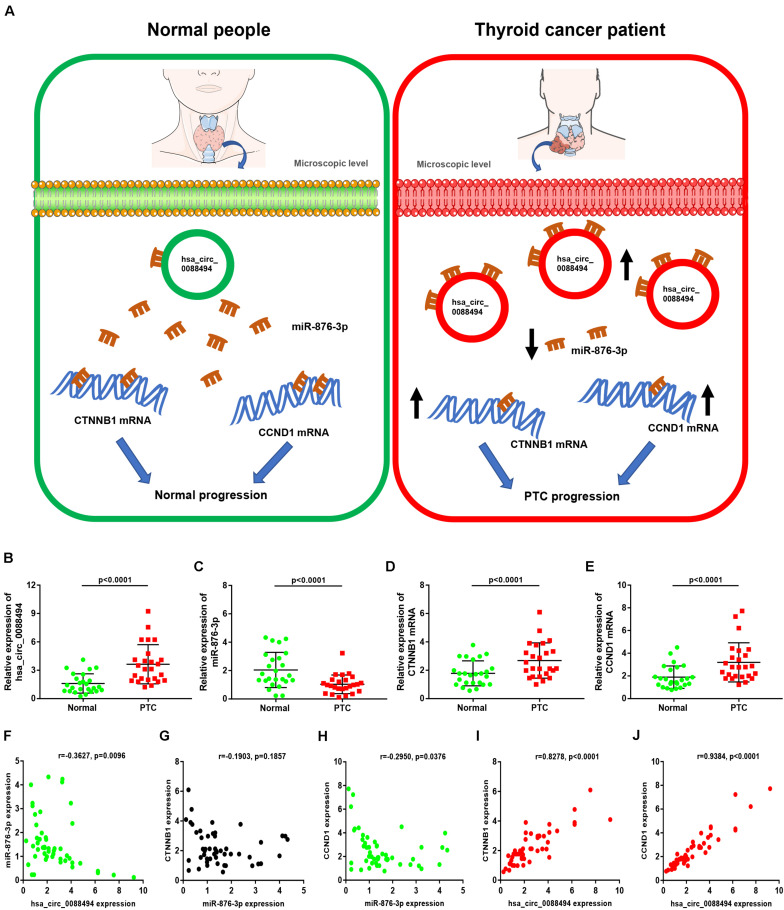
Construction and validation of a potential axis (hsa_circ_0088494-miR-876-3p-CTNNB1/CCND1) involved in progression of thyroid carcinoma. **(A)** The model of hsa_circ_0088494-miR-876-3p-CTNNB1/CCND1 axis in PTC. **(B)** Hsa_circ_0088494 expression in 25 pairs PTC and adjacent normal tissues. **(C)** MiR-876-3p expression in 25 pairs PTC and adjacent normal tissues. **(D)** CTNNB1 mRNA expression in 25 pairs PTC and adjacent normal tissues. **(E)** CCND1 mRNA expression in 25 pairs PTC and adjacent normal tissues. **(F)** Expression correlation of hsa_circ_0088494 with miR-876-3p in PTC. **(G)** Expression correlation of CTNNB1 with miR-876-3p in PTC. **(H)** Expression correlation of CCND1 with miR-876-3p in PTC. **(I)** Expression correlation of hsa_circ_0088494 with CTNNB1 in PTC. **(J)** Expression correlation of hsa_circ_0088494 with CCND1 in PTC. “*p* < 0.05” was considered as statistically significant.

Subsequently, a miRNA-hub gene network, consisting of miR-605-5p, miR-876-3p, and the top 20 hub genes, was established using Cytoscape (Figure 7A). The expression correlation between miRNAs and their respective target genes in thyroid carcinoma were evaluated by the starBase database (Table 4). As presented in Figures 7B–D, only three miRNA–gene pairs (miR-876-3p-CTNNB1, miR-876-3p-ACTB, and miR-876-3p-CCND1) revealed a statistically negative expression relationship in thyroid carcinoma. Next, the expression levels of CTNNB1, ACTB, and CCND1 in thyroid carcinoma were also determined (Figures 7E–G). Among the three genes, only CTNNB1 and CCND1 expression was remarkably increased in thyroid carcinoma tissues when compared with normal thyroid tissues. These findings suggested that CTNNB1 and CCND1 might be two downstream key targets of miR-876-3p in PTC. Taken together, dysregulation of a potential pathway, namely, hsa_circ_0088494-miR-876-3p-CTNNB1/CCND1, might play important roles in the process of PTC carcinogenesis and development (Figure 8A). Finally, 25 pairs of clinical PTC tissues and adjacent normal tissues were employed to preliminarily validate RNA expression and RNA-RNA correlation of the established potential pathway in PTC. As shown in Figures 8B–E, expression levels of hsa_circ_0088494, CTNNB1, and CCND1 were significantly increased but miR-876-3p was markedly downregulated in PTC tissues when compared with normal tissues. These findings were identical with our previous analytic results. Furthermore, despite that no statistical expression correlation was observed between the miR-876-3p/CTNNB1 RNA pair, miR-876-3p was negatively correlated with hsa_circ_0088494, CTNNB1, or CCND1 and hsa_circ_0088494 was positively linked to CTNNB1 or CCND1 in PTC (Figures 8F–J), which was in accordance with competing endogenous RNA (ceRNA) hypothesis (Salmena et al., 2011).

**TABLE 4:** The expression correlation between miRNA and hub gene in thyroid carcinoma determined by the starBase database.

Hub gene	miRNA	*R*	*P*-value
TP53	miR-605-5p	−0.095	3.15E-02
GAPDH	miR-876-3p	0.13	3.33E-03
CTNNB1	miR-605-5p	−0.05	2.58E-01
CTNNB1	**miR-876-3p**	−**0.186**	**2.36E-05**
ACTB	miR-605-5p	−0.053	2.33E-01
ACTB	**miR-876-3p**	−**0.138**	**1.76E-03**
EEF2	miR-876-3p	−0.053	2.37E-01
CCND1	**miR-876-3p**	−**0.237**	**6.28E-08**
VEGFA	miR-876-3p	0.305	2.13E-12
JUN	miR-876-3p	0.176	6.44E-05
HNRNPK	miR-605-5p	−0.027	5.40E-01
XPO1	miR-876-3p	0.046	2.97E-01
MDM2	miR-605-5p	0.035	4.37E-01
HNRNPA2B1	miR-605-5p	0.138	1.77E-03
NCBP2	miR-605-5p	−0.064	1.50E-01
DHX9	miR-876-3p	−0.053	2.36E-01
DICER1	miR-605-5p	0.269	6.61E-10
HNRNPD	miR-605-5p	0.122	6.03E-03
NCL	miR-605-5p	−0.025	5.79E-01
DDX5	miR-605-5p	0.184	3.04E-05
YBX1	miR-605-5p	0.033	4.59E-01
PCBP2	miR-876-3p	0.135	2.25E-03

*Bold values represent the pairs with statistical significance.*

## Discussion

Papillary thyroid carcinoma is one of the most common human cancer types worldwide. Despite that the overall outcome of patients with PTC is good, the detailed molecular mechanism of PTC pathogenesis is not fully elucidated and needs to be investigated to further improve prognosis of patients with PTC.

To uncover the expression and mechanism of circRNA in PTC, GEO2R was used to perform differential expression analysis for the GSE93522 dataset from the GEO database. A total of 14 candidate circRNAs were identified, which were only significantly dysregulated in PTC. Some of these circRNAs have been documented to be closely associated with initiation and progression of human cancers. For example, hsa_circ_0001955 was linked to development of colorectal cancer and breast cancer (Afzali and Salimi, 2019; Ding et al., 2020).

Salmena et al. (2011) proposed that ncRNAs, including circRNAs, could “talk” to messenger RNAs (mRNAs) using microRNA response elements (MREs). Recent studies have also experimentally validated that circRNAs could modulate gene expression through sponging shared miRNAs, thus influencing PTC progression. For example, Wei et al. (2018) indicated that circZFR facilitated cell proliferation and invasion of PTC by upregulating c8orf4 through sponging miR-1261; Li et al. (2018) suggested that circNUP214 sponged miR-145 to enhance ZEB2 expression in PTC cells.

Thus, the binding miRNAs of these candidate circRNAs were predicted by combination of two databases, namely, CSCD and CRI. Finally, 21 miRNAs targeting 9 circRNAs were found. The 9 circRNAs were all significantly upregulated in PTC when compared with normal thyroid tissues. In accordance with ceRNA hypothesis, there is a negative relationship between circRNA and its binding miRNA, indicating that miRNAs should be tumor-suppressive modulators in PTC. Therefore, expression analysis and survival analysis were performed to improve the analytic accuracy. Consequently, two miRNAs, miR-605-5p and miR-876-3p, were identified as the most potential binding miRNAs of candidate circRNAs. Despite that the roles of the two miRNAs in PTC have not been studied, miR-605-5p and miR-876-3p have been found to act as tumor-suppressor genes in human cancers. For example, miR-605-5p was markedly downregulated in melanoma tissues and cells and inhibited melanoma progression and glutamine catabolism through targeting GLS (Lu et al., 2020); miR-876-3p functioned as a tumor suppressor and correlated with cell metastasis in pancreatic adenocarcinoma via targeting JAG2 (Yang et al., 2018).

To understand the downstream action mechanism of miR-605-5p and miR-876-3p, their target genes were predicted using a comprehensive target gene prediction database, namely, miRNet. It has been widely acknowledged that genes usually exert their functions by interacting with each other. Thus, these predicted target genes were entered into the STRING database to conduct a PPI network analysis, and the top 20 hub genes in this PPI network were screened, after which a miRNA–hub gene network was established. Based on the action mechanism of miRNA, there should be a negative expression relationship between miRNAs and target genes, and target genes of tumor suppressive miRNAs should function as oncogenes. At the end, CTNNB1 and CCND1 were screened out, which were both the potential targets of miR-876-3p. CTNNB1 is part of a complex of proteins that constitute adherens junctions, which is correlated with cancer pathogenesis (Ilina et al., 2020; Ye et al., 2020; Zhang et al., 2020; Zhitnyak et al., 2020). CCND1 belongs to the highly conserved cyclin family, whose members are featured by a dramatic periodicity in protein abundance throughout the cell cycle. Recent studies have also suggested that CCND1 is involved in the process of cancer initiation and progression in multiple cancers, including breast cancer (Montaudon et al., 2020), lung cancer (Yang Y. et al., 2020), and bladder cancer (Chen et al., 2019; Ying et al., 2020). Besides, CCND1 and CTNNB1 were also reported to function as oncogenes in thyroid cancer (Jung et al., 2009; Guo et al., 2019; Wong et al., 2019; Li et al., 2020). The previous analytic results together with these reports indicated that hsa_circ_0088494-miR-876-3p might influence CTNNB1 and CCND1 expression and function, thereby exerting their roles in mediating cancer carcinogenesis and progression.

In conclusion, our study elucidated a potential pathway, namely, hsa_circ_0088494-miR-876-3p-CTNNB1/CCND1 axis, in PTC by combination of a series of bioinformatic analyses, which may provide key clues for developing effective therapeutic targets in treating patients with PTC. However, the current findings need to be further validated by basic experiments and clinical trials in the future.

## Materials and Methods

### Inclusion of Dataset

In this study, we aimed to identify some potential functional circRNAs in initiation and progression of PTC. The NCBI GEO database^1^ was used to screen possible datasets. Selection criteria are as follows: (1) The selected datasets should contain normal thyroid tissues, benign thyroid lesions, and PTC tissues; (2) the sample count of selected datasets should be more than 10; and (3) datasets about human PTC cells or animal PTC tissues should be excluded. Finally, we found that only GSE93522 met all these criteria. GSE93522 contained a total of 18 thyroid samples, consisting of six normal thyroid tissues, six benign thyroid lesions, and six PTC tumors. GSE93522, based on the platform of GPL19978 Agilent-069978 Arrarystar Human CircRNA microarray V1, studied the circRNA expression profile in different thyroid tissues.

### Differential Analysis

To obtain the differentially expressed circRNAs (DECs) associated with carcinogenesis and progression of PTC, differential analysis was performed using GEO2R^2^ which is an online tool provided by the NCBI GEO database as previously described (Ding et al., 2020). | log_2_FC| > 1 and *P* < 0.05 were set as the thresholds for identifying DECs.

### circBase Analysis

circBase^3^ is a database for investigating circRNA-associated studies, which provides scripts to identify known and novel circRNAs in sequencing data and where merged and unified data sets of circRNAs and the evidence supporting their expression can be accessed, downloaded, and browsed within the genomic context (Glažar et al., 2014). In our study, the circBase database was introduced to acquire location and parental genes of candidate circRNAs.

### Cancer-Specific circRNA Database (CSCD) Analysis

Cancer-specific circRNA database^4^ is a database for cancer-specific circular RNAs, contributing to the research for the function and regulation of cancer-associated circRNAs (Xia et al., 2018). The CSCD database was employed to obtain structural loop graphs of candidate circRNAs and predict the potential microRNA response element (MRE) sites for each candidate circRNAs.

### Circular RNA Interactome (CRI) Analysis

Circular RNA Interactome^5^, a web tool for exploring circular RNAs and their interacting proteins and microRNAs, was also introduced to predict binding miRNAs of candidate circRNAs (Dudekula et al., 2016). The predicted miRNAs that potentially bind to candidate circRNAs were directly exported from the CRI database.

### starBase Analysis

starBase database^6^ is an open-source platform for decoding miRNA–ceRNA, miRNA–ncRNA, and protein–RNA interaction networks from 108 CLIP-seq data sets generated from 37 independent studies (Yang et al., 2011; Li et al., 2014). In this study, starBase was utilized to analyze the expression levels of miRNAs and target genes in thyroid carcinoma. Moreover, this database was used to assess the expression correlation of miRNAs with their corresponding target genes in thyroid carcinoma. *R* < -0.1 and *P* < 0.05 were considered as statistically significant.

### Kaplan-Meier Plotter Analysis

As we previously described (Lou et al., 2019a,b), the prognostic values of miRNAs in thyroid carcinoma were evaluated by the Kaplan–Meier plotter database^7^, which is an online tool capable of accessing the effect of 54,000 genes on survival in 21 cancer types, and its miRNA subsystems include 11,000 samples from 20 distinct cancer types, containing thyroid carcinoma.

### miRNet Analysis

miRNet^8^ is an integrated open-source platform linking to miRNAs, targets, and their functions, which was used to predict potential target genes of miRNAs in this study (Fan and Xia, 2018; Chang et al., 2020).

### STRING Analysis

The protein–protein interaction (PPI) network analysis and enrichment analysis for these target genes of potential miRNAs were conducted by the STRING database^9^. The top 5 enriched GO items (including three categories, biological process, cellular component, and molecular function) and KEGG pathways were directly cut from the online webpage. Furthermore, the interaction gene pairs in the established PPI network were downloaded from the STRING database.

### Cytoscape Software

The interaction gene pairs downloaded from the STRING database were reentered into Cytoscape software, after which hub genes could be obtained after calculated by Cytohubb. circRNA–miRNA or miRNA–hub gene pairs were also reentered into Cytoscape software, and the circRNA–miRNA or miRNA–hub gene network was established and downloaded.

## Data Availability Statement

The original contributions presented in the study are included in the article/Supplementary Materials, further inquiries can be directed to the corresponding author/s.

## Author Contributions

WL designed this work, performed the *in silico* analyses and experiments, and wrote the manuscript. BD and JW performed some *in silico* analyses. YX polished the manuscript. All authors have read the final version of this manuscript.

## Conflict of Interest

The authors declare that the research was conducted in the absence of any commercial or financial relationships that could be construed as a potential conflict of interest.

## Footnotes

1 http://www.ncbi.nlm.nih.gov/geo/

2 http://www.ncbi.nlm.nih.gov/geo/geo2r/

3 http://www.circbase.org/

4 http://www.circbase.org/

5 https://circinteractome.nia.nih.gov/

6 http://starbase.sysu.edu.cn/

7 http://kmplot.com/analysis/

8 http://www.mirnet.ca/

9 http://string-db.org
