# Supplementary material for: The Involvement of the hsa_circ_0088494-miR-876-3p-CTNNB1/CCND1 Axis in Carcinogenesis and Progression of Papillary Thyroid Carcinoma
Source: Front Cell Dev Biol. 2020 Dec 9;8:605940. doi: 10.3389/fcell.2020.605940 (PMC7755655; doi:10.3389/fcell.2020.605940)
Supplement: Supplementary file 1 [file Table_1.DOCX]

**Supplemental information**

**Table S1**. The potential target genes predicted by miRNet database.

| miRNA name | Target |
| --- | --- |
| hsa-mir-605-5p | *ABCB7* |
| hsa-mir-605-5p | *ABL2* |
| hsa-mir-605-5p | *ACTB* |
| hsa-mir-605-5p | *ACTG1* |
| hsa-mir-605-5p | *ACTN4* |
| hsa-mir-605-5p | *ABCD2* |
| hsa-mir-605-5p | *BCL9* |
| hsa-mir-605-5p | *CACNA1A* |
| hsa-mir-605-5p | *CACNA1E* |
| hsa-mir-605-5p | *CAPZA2* |
| hsa-mir-605-5p | *RUNX1* |
| hsa-mir-605-5p | *CYLD* |
| hsa-mir-605-5p | *RCAN1* |
| hsa-mir-605-5p | *DUSP2* |
| hsa-mir-605-5p | *DUSP3* |
| hsa-mir-605-5p | *EDN3* |
| hsa-mir-605-5p | *EPHA4* |
| hsa-mir-605-5p | *GLO1* |
| hsa-mir-605-5p | *GPM6B* |
| hsa-mir-605-5p | *H3F3B* |
| hsa-mir-605-5p | *HOXA13* |
| hsa-mir-605-5p | *HOXB2* |
| hsa-mir-605-5p | *KCNJ13* |
| hsa-mir-605-5p | *LDLR* |
| hsa-mir-605-5p | *MDM2* |
| hsa-mir-605-5p | *DRG1* |
| hsa-mir-605-5p | *NFIB* |
| hsa-mir-605-5p | *YBX1* |
| hsa-mir-605-5p | *POU2F1* |
| hsa-mir-605-5p | *PSMD10* |
| hsa-mir-605-5p | *PTGIS* |
| hsa-mir-605-5p | *SCD* |
| hsa-mir-605-5p | *CX3CL1* |
| hsa-mir-605-5p | *SIX1* |
| hsa-mir-605-5p | *SLC16A1* |
| hsa-mir-605-5p | *STAT1* |
| hsa-mir-605-5p | *STK4* |
| hsa-mir-605-5p | *TP53* |
| hsa-mir-605-5p | *NR2C2* |
| hsa-mir-605-5p | *WEE1* |
| hsa-mir-605-5p | *ZNF12* |
| hsa-mir-605-5p | *PRDM2* |
| hsa-mir-605-5p | *DEK* |
| hsa-mir-605-5p | *PRRC2A* |
| hsa-mir-605-5p | *GAN* |
| hsa-mir-605-5p | *KDM5C* |
| hsa-mir-605-5p | *SMC1A* |
| hsa-mir-605-5p | *SPOP* |
| hsa-mir-605-5p | *LIMD1* |
| hsa-mir-605-5p | *BUB3* |
| hsa-mir-605-5p | *TCEAL1* |
| hsa-mir-605-5p | *TRAF4* |
| hsa-mir-605-5p | *MDC1* |
| hsa-mir-605-5p | *IP6K1* |
| hsa-mir-605-5p | *TSC22D2* |
| hsa-mir-605-5p | *SEC24D* |
| hsa-mir-605-5p | *SLC35E2* |
| hsa-mir-605-5p | *SLC25A13* |
| hsa-mir-605-5p | *ALYREF* |
| hsa-mir-605-5p | *DCAF7* |
| hsa-mir-605-5p | *CAP2* |
| hsa-mir-605-5p | *IVNS1ABP* |
| hsa-mir-605-5p | *SLC12A7* |
| hsa-mir-605-5p | *CPSF6* |
| hsa-mir-605-5p | *ZNF507* |
| hsa-mir-605-5p | *FBXO21* |
| hsa-mir-605-5p | *CAPN7* |
| hsa-mir-605-5p | *CHORDC1* |
| hsa-mir-605-5p | *GTPBP8* |
| hsa-mir-605-5p | *TMX2* |
| hsa-mir-605-5p | *SIX4* |
| hsa-mir-605-5p | *CEP55* |
| hsa-mir-605-5p | *CENPQ* |
| hsa-mir-605-5p | *MCOLN3* |
| hsa-mir-605-5p | *ST6GALNAC1* |
| hsa-mir-605-5p | *SULF2* |
| hsa-mir-605-5p | *TIGAR* |
| hsa-mir-605-5p | *RTN4* |
| hsa-mir-605-5p | *ZSWIM6* |
| hsa-mir-605-5p | *MANBAL* |
| hsa-mir-605-5p | *COPS7B* |
| hsa-mir-605-5p | *CENPH* |
| hsa-mir-605-5p | *LONRF3* |
| hsa-mir-605-5p | *DSN1* |
| hsa-mir-605-5p | *ZCCHC9* |
| hsa-mir-605-5p | *HES7* |
| hsa-mir-605-5p | *BTF3L4* |
| hsa-mir-605-5p | *SFXN5* |
| hsa-mir-605-5p | *ANAPC16* |
| hsa-mir-605-5p | *SPPL3* |
| hsa-mir-605-5p | *TTC8* |
| hsa-mir-605-5p | *GGT6* |
| hsa-mir-605-5p | *GJD3* |
| hsa-mir-605-5p | *ARL9* |
| hsa-mir-605-5p | *AASDH* |
| hsa-mir-605-5p | *SLC38A9* |
| hsa-mir-605-5p | *KIAA1958* |
| hsa-mir-605-5p | *TMEM64* |
| hsa-mir-605-5p | *NBPF11* |
| hsa-mir-605-5p | *ELMOD2* |
| hsa-mir-605-5p | *SGMS1* |
| hsa-mir-605-5p | *ZNF740* |
| hsa-mir-605-5p | *C17orf105* |
| hsa-mir-605-5p | *ZNF678* |
| hsa-mir-605-5p | *NANOS1* |
| hsa-mir-605-5p | *GEN1* |
| hsa-mir-605-5p | *H3F3C* |
| hsa-mir-605-5p | *ZFP62* |
| hsa-mir-605-5p | *ANKRD33B* |
| hsa-mir-605-5p | *GXYLT2* |
| hsa-mir-605-5p | *C16orf52* |
| hsa-mir-876-3p | *ACTC1* |
| hsa-mir-876-3p | *CRISP1* |
| hsa-mir-876-3p | *CCND1* |
| hsa-mir-876-3p | *CEBPD* |
| hsa-mir-876-3p | *CRKL* |
| hsa-mir-876-3p | *ELN* |
| hsa-mir-876-3p | *EPHB4* |
| hsa-mir-876-3p | *SLC37A4* |
| hsa-mir-876-3p | *TM4SF1* |
| hsa-mir-876-3p | *MCL1* |
| hsa-mir-876-3p | *MDM4* |
| hsa-mir-876-3p | *PPIA* |
| hsa-mir-876-3p | *PPIC* |
| hsa-mir-876-3p | *RBBP6* |
| hsa-mir-876-3p | *ROBO1* |
| hsa-mir-876-3p | *S100A11* |
| hsa-mir-876-3p | *TRIM21* |
| hsa-mir-876-3p | *YWHAE* |
| hsa-mir-876-3p | *DDX39B* |
| hsa-mir-876-3p | *ARID1A* |
| hsa-mir-876-3p | *HIST1H3D* |
| hsa-mir-876-3p | *SGPL1* |
| hsa-mir-876-3p | *RPL14* |
| hsa-mir-876-3p | *NDST3* |
| hsa-mir-876-3p | *ENTPD4* |
| hsa-mir-876-3p | *RUBCN* |
| hsa-mir-876-3p | *STARD8* |
| hsa-mir-876-3p | *TRIM38* |
| hsa-mir-876-3p | *DMRT2* |
| hsa-mir-876-3p | *ZNF609* |
| hsa-mir-876-3p | *EMC1* |
| hsa-mir-876-3p | *PEG10* |
| hsa-mir-876-3p | *TTLL12* |
| hsa-mir-876-3p | *HAUS5* |
| hsa-mir-876-3p | *VPS8* |
| hsa-mir-876-3p | *SF3B3* |
| hsa-mir-876-3p | *KPNA6* |
| hsa-mir-876-3p | *CNNM4* |
| hsa-mir-876-3p | *BBC3* |
| hsa-mir-876-3p | *PRICKLE4* |
| hsa-mir-876-3p | *PDE11A* |
| hsa-mir-876-3p | *PIGP* |
| hsa-mir-876-3p | *CRIM1* |
| hsa-mir-876-3p | *MTRF1L* |
| hsa-mir-876-3p | *USP53* |
| hsa-mir-876-3p | *SLC38A7* |
| hsa-mir-876-3p | *ZFP14* |
| hsa-mir-876-3p | *CDK15* |
| hsa-mir-876-3p | *ZNF552* |
| hsa-mir-876-3p | *TNIP3* |
| hsa-mir-876-3p | *ZNF394* |
| hsa-mir-876-3p | *FRMPD3* |
| hsa-mir-876-3p | *MIGA2* |
| hsa-mir-876-3p | *DPP9* |
| hsa-mir-876-3p | *SDR9C7* |
| hsa-mir-876-3p | *AMER1* |
| hsa-mir-876-3p | *UBE2F* |
| hsa-mir-876-3p | *SIRPA* |
| hsa-mir-876-3p | *C11orf84* |
| hsa-mir-876-3p | *CNKSR3* |
| hsa-mir-876-3p | *CRTC2* |
| hsa-mir-876-3p | *OR9Q1* |
| hsa-mir-876-3p | *SGMS1* |
| hsa-mir-876-3p | *GEN1* |
| hsa-mir-876-3p | *BLOC1S3* |
| hsa-mir-876-3p | *LIN28B* |
| hsa-mir-876-3p | *POTEG* |
| hsa-mir-876-3p | *ZNF724* |
| hsa-mir-876-3p | *POTEM* |
| hsa-mir-876-3p | *TMEM170B* |
| hsa-mir-605-5p | *ACTG1* |
| hsa-mir-605-5p | *BCL9* |
| hsa-mir-605-5p | *GLO1* |
| hsa-mir-605-5p | *MDM2* |
| hsa-mir-605-5p | *YBX1* |
| hsa-mir-605-5p | *POU2F1* |
| hsa-mir-605-5p | *SCD* |
| hsa-mir-605-5p | *TP53* |
| hsa-mir-605-5p | *DEK* |
| hsa-mir-605-5p | *GAN* |
| hsa-mir-605-5p | *CPSF6* |
| hsa-mir-605-5p | *ZNF507* |
| hsa-mir-605-5p | *SIX4* |
| hsa-mir-605-5p | *RTN4* |
| hsa-mir-605-5p | *BTF3L4* |
| hsa-mir-605-5p | *TMEM64* |
| hsa-mir-605-5p | *ELMOD2* |
| hsa-mir-876-3p | *CCND1* |
| hsa-mir-876-3p | *TM4SF1* |
| hsa-mir-876-3p | *MCL1* |
| hsa-mir-876-3p | *YWHAE* |
| hsa-mir-876-3p | *PEG10* |
| hsa-mir-876-3p | *CRIM1* |
| hsa-mir-876-3p | *ARF5* |
| hsa-mir-876-3p | *KDM1A* |
| hsa-mir-876-3p | *ITGA2B* |
| hsa-mir-876-3p | *SPATA20* |
| hsa-mir-605-5p | *PAFAH1B1* |
| hsa-mir-605-5p | *SYPL1* |
| hsa-mir-605-5p | *MAPK8IP2* |
| hsa-mir-605-5p | *ZNF207* |
| hsa-mir-605-5p | *CD9* |
| hsa-mir-876-3p | *NCAPD2* |
| hsa-mir-605-5p | *NISCH* |
| hsa-mir-876-3p | *HIVEP2* |
| hsa-mir-605-5p | *DNASE1L1* |
| hsa-mir-605-5p | *CCDC28A* |
| hsa-mir-605-5p | *SEC63* |
| hsa-mir-605-5p | *AGPAT4* |
| hsa-mir-876-3p | *INSRR* |
| hsa-mir-605-5p | *NUP160* |
| hsa-mir-605-5p | *NUP160* |
| hsa-mir-876-3p | *FBXO42* |
| hsa-mir-876-3p | *PHF23* |
| hsa-mir-605-5p | *TNC* |
| hsa-mir-876-3p | *TNC* |
| hsa-mir-605-5p | *DSG2* |
| hsa-mir-876-3p | *ARID1B* |
| hsa-mir-605-5p | *MPHOSPH9* |
| hsa-mir-876-3p | *THRAP3* |
| hsa-mir-605-5p | *PLEKHH1* |
| hsa-mir-876-3p | *TRAF1* |
| hsa-mir-605-5p | *USP13* |
| hsa-mir-605-5p | *CAMK2B* |
| hsa-mir-876-3p | *YBX3* |
| hsa-mir-876-3p | *WNK1* |
| hsa-mir-605-5p | *TNK2* |
| hsa-mir-876-3p | *RNF4* |
| hsa-mir-605-5p | *TAF2* |
| hsa-mir-605-5p | *HIPK2* |
| hsa-mir-876-3p | *SBNO2* |
| hsa-mir-876-3p | *SBNO2* |
| hsa-mir-605-5p | *SPEN* |
| hsa-mir-605-5p | *SNAP91* |
| hsa-mir-605-5p | *ASB1* |
| hsa-mir-876-3p | *ATXN3* |
| hsa-mir-605-5p | *FGFR2* |
| hsa-mir-876-3p | *THUMPD1* |
| hsa-mir-605-5p | *FECH* |
| hsa-mir-605-5p | *ACSL4* |
| hsa-mir-605-5p | *IP6K2* |
| hsa-mir-876-3p | *NUCKS1* |
| hsa-mir-605-5p | *VPS35* |
| hsa-mir-876-3p | *SLC44A1* |
| hsa-mir-605-5p | *RPS6KA2* |
| hsa-mir-876-3p | *EVC* |
| hsa-mir-605-5p | *PVR* |
| hsa-mir-605-5p | *KDM5A* |
| hsa-mir-605-5p | *PPP2R3A* |
| hsa-mir-876-3p | *IGF2BP2* |
| hsa-mir-876-3p | *NTN4* |
| hsa-mir-605-5p | *SLC25A3* |
| hsa-mir-876-3p | *ACTB* |
| hsa-mir-876-3p | *DLG1* |
| hsa-mir-605-5p | *TPD52* |
| hsa-mir-876-3p | *TRAF4* |
| hsa-mir-605-5p | *TM9SF3* |
| hsa-mir-605-5p | *UBE2A* |
| hsa-mir-605-5p | *PIK3C3* |
| hsa-mir-876-3p | *HOXA9* |
| hsa-mir-605-5p | *SESN1* |
| hsa-mir-876-3p | *SRCAP* |
| hsa-mir-876-3p | *XPO1* |
| hsa-mir-605-5p | *WBP11* |
| hsa-mir-876-3p | *WBP11* |
| hsa-mir-605-5p | *NME8* |
| hsa-mir-605-5p | *RBM22* |
| hsa-mir-876-3p | *SF3B2* |
| hsa-mir-605-5p | *KLHL42* |
| hsa-mir-605-5p | *AURKA* |
| hsa-mir-876-3p | *TPX2* |
| hsa-mir-876-3p | *XRN2* |
| hsa-mir-605-5p | *PXN* |
| hsa-mir-605-5p | *HEPH* |
| hsa-mir-605-5p | *RBM41* |
| hsa-mir-876-3p | *CBX5* |
| hsa-mir-605-5p | *MSH2* |
| hsa-mir-876-3p | *MYO9B* |
| hsa-mir-876-3p | *FBXL19* |
| hsa-mir-605-5p | *CIRBP* |
| hsa-mir-876-3p | *CECR2* |
| hsa-mir-876-3p | *EIF3L* |
| hsa-mir-876-3p | *RBFOX2* |
| hsa-mir-876-3p | *RBFOX2* |
| hsa-mir-876-3p | *MTMR3* |
| hsa-mir-876-3p | *SDR39U1* |
| hsa-mir-876-3p | *SOS2* |
| hsa-mir-605-5p | *CNIH1* |
| hsa-mir-605-5p | *DICER1* |
| hsa-mir-605-5p | *ACIN1* |
| hsa-mir-876-3p | *BRMS1L* |
| hsa-mir-876-3p | *DHX35* |
| hsa-mir-605-5p | *ADNP2* |
| hsa-mir-605-5p | *VAPA* |
| hsa-mir-876-3p | *EMD* |
| hsa-mir-876-3p | *MGRN1* |
| hsa-mir-876-3p | *PSMD7* |
| hsa-mir-605-5p | *MAZ* |
| hsa-mir-876-3p | *MAZ* |
| hsa-mir-605-5p | *TJP1* |
| hsa-mir-605-5p | *TJP1* |
| hsa-mir-605-5p | *IMPAD1* |
| hsa-mir-876-3p | *EEF1D* |
| hsa-mir-876-3p | *EEF1D* |
| hsa-mir-876-3p | *PTOV1* |
| hsa-mir-605-5p | *AKT2* |
| hsa-mir-876-3p | *AKT2* |
| hsa-mir-605-5p | *MEGF8* |
| hsa-mir-876-3p | *TNPO2* |
| hsa-mir-876-3p | *ZNF85* |
| hsa-mir-876-3p | *ZNF85* |
| hsa-mir-876-3p | *AVL9* |
| hsa-mir-605-5p | *CDK6* |
| hsa-mir-605-5p | *MTPN* |
| hsa-mir-876-3p | *WASL* |
| hsa-mir-605-5p | *RBM28* |
| hsa-mir-876-3p | *CLDN15* |
| hsa-mir-605-5p | *PLEKHA1* |
| hsa-mir-605-5p | *TNKS2* |
| hsa-mir-876-3p | *TFAM* |
| hsa-mir-876-3p | *UBTF* |
| hsa-mir-605-5p | *CPD* |
| hsa-mir-605-5p | *DDX5* |
| hsa-mir-876-3p | *EZH1* |
| hsa-mir-605-5p | *TBC1D9* |
| hsa-mir-605-5p | *TRIM2* |
| hsa-mir-876-3p | *CBL* |
| hsa-mir-605-5p | *MLEC* |
| hsa-mir-876-3p | *DUSP16* |
| hsa-mir-876-3p | *DUSP16* |
| hsa-mir-605-5p | *RAB5B* |
| hsa-mir-876-3p | *CPSF6* |
| hsa-mir-876-3p | *GAPDH* |
| hsa-mir-876-3p | *ATN1* |
| hsa-mir-605-5p | *SRSF9* |
| hsa-mir-605-5p | *CEP85L* |
| hsa-mir-605-5p | *KCTD20* |
| hsa-mir-876-3p | *STK38* |
| hsa-mir-605-5p | *SOD2* |
| hsa-mir-605-5p | *SOD2* |
| hsa-mir-605-5p | *ZNF451* |
| hsa-mir-876-3p | *C6orf62* |
| hsa-mir-605-5p | *PHACTR2* |
| hsa-mir-605-5p | *EXOC2* |
| hsa-mir-876-3p | *VEGFA* |
| hsa-mir-876-3p | *DROSHA* |
| hsa-mir-605-5p | *CSNK1A1* |
| hsa-mir-605-5p | *CPEB4* |
| hsa-mir-605-5p | *XRN1* |
| hsa-mir-605-5p | *NCBP2* |
| hsa-mir-605-5p | *ACVR2B* |
| hsa-mir-876-3p | *ZBTB47* |
| hsa-mir-876-3p | *EIF4G1* |
| hsa-mir-605-5p | *NCL* |
| hsa-mir-605-5p | *GALNT3* |
| hsa-mir-876-3p | *GORASP2* |
| hsa-mir-605-5p | *SRSF7* |
| hsa-mir-605-5p | *ATF2* |
| hsa-mir-605-5p | *PLEKHA3* |
| hsa-mir-876-3p | *PARD3B* |
| hsa-mir-605-5p | *DHCR24* |
| hsa-mir-876-3p | *CACYBP* |
| hsa-mir-605-5p | *ERRFI1* |
| hsa-mir-605-5p | *SLC19A2* |
| hsa-mir-876-3p | *KMT2A* |
| hsa-mir-876-3p | *SGK1* |
| hsa-mir-605-5p | *RAB14* |
| hsa-mir-605-5p | *RBM25* |
| hsa-mir-876-3p | *BCL11A* |
| hsa-mir-605-5p | *PANK3* |
| hsa-mir-605-5p | *MASTL* |
| hsa-mir-605-5p | *ETF1* |
| hsa-mir-605-5p | *SERP1* |
| hsa-mir-876-3p | *RNF2* |
| hsa-mir-605-5p | *NAA50* |
| hsa-mir-876-3p | *CLCC1* |
| hsa-mir-605-5p | *MTERF4* |
| hsa-mir-605-5p | *HNRNPA2B1* |
| hsa-mir-876-3p | *INHBA* |
| hsa-mir-605-5p | *SLC25A51* |
| hsa-mir-605-5p | *DCAF10* |
| hsa-mir-605-5p | *IFT81* |
| hsa-mir-605-5p | *DDX39A* |
| hsa-mir-605-5p | *CDK2* |
| hsa-mir-876-3p | *AMD1* |
| hsa-mir-605-5p | *MORF4L2* |
| hsa-mir-876-3p | *LPGAT1* |
| hsa-mir-605-5p | *PREX1* |
| hsa-mir-876-3p | *PREX1* |
| hsa-mir-876-3p | *ZNFX1* |
| hsa-mir-876-3p | *STX16* |
| hsa-mir-605-5p | *VAMP7* |
| hsa-mir-876-3p | *USP22* |
| hsa-mir-605-5p | *KLHDC3* |
| hsa-mir-876-3p | *SOX4* |
| hsa-mir-605-5p | *AHNAK* |
| hsa-mir-876-3p | *AHNAK* |
| hsa-mir-605-5p | *CNOT1* |
| hsa-mir-605-5p | *TM9SF2* |
| hsa-mir-605-5p | *RPL23* |
| hsa-mir-876-3p | *SNRPB* |
| hsa-mir-876-3p | *DYNLRB1* |
| hsa-mir-876-3p | *AMOT* |
| hsa-mir-605-5p | *UROD* |
| hsa-mir-605-5p | *CANX* |
| hsa-mir-605-5p | *CANX* |
| hsa-mir-605-5p | *DYRK2* |
| hsa-mir-876-3p | *FBXL16* |
| hsa-mir-876-3p | *ZFP36* |
| hsa-mir-876-3p | *TWSG1* |
| hsa-mir-876-3p | *CLN6* |
| hsa-mir-605-5p | *SPCS3* |
| hsa-mir-605-5p | *E2F8* |
| hsa-mir-605-5p | *PHF20L1* |
| hsa-mir-605-5p | *ILF3* |
| hsa-mir-605-5p | *TULP4* |
| hsa-mir-876-3p | *MLLT1* |
| hsa-mir-876-3p | *ACTN4* |
| hsa-mir-876-3p | *ACTN4* |
| hsa-mir-876-3p | *PRRC2B* |
| hsa-mir-605-5p | *LATS1* |
| hsa-mir-605-5p | *AAR2* |
| hsa-mir-876-3p | *TTLL9* |
| hsa-mir-605-5p | *SLC6A6* |
| hsa-mir-876-3p | *PODNL1* |
| hsa-mir-876-3p | *PODNL1* |
| hsa-mir-605-5p | *PRKAA1* |
| hsa-mir-876-3p | *MYBBP1A* |
| hsa-mir-605-5p | *PNISR* |
| hsa-mir-605-5p | *GRSF1* |
| hsa-mir-876-3p | *KDM6B* |
| hsa-mir-605-5p | *ALDH3B2* |
| hsa-mir-605-5p | *MPRIP* |
| hsa-mir-876-3p | *IRS4* |
| hsa-mir-876-3p | *IRS4* |
| hsa-mir-605-5p | *FOPNL* |
| hsa-mir-605-5p | *FOPNL* |
| hsa-mir-876-3p | *ADCK2* |
| hsa-mir-605-5p | *AGAP3* |
| hsa-mir-876-3p | *BTG1* |
| hsa-mir-605-5p | *ATP13A3* |
| hsa-mir-605-5p | *TTC9* |
| hsa-mir-605-5p | *KATNBL1* |
| hsa-mir-605-5p | *NOTCH2* |
| hsa-mir-876-3p | *NOTCH2* |
| hsa-mir-605-5p | *SLC38A2* |
| hsa-mir-605-5p | *TIMM17A* |
| hsa-mir-876-3p | *LRP4* |
| hsa-mir-876-3p | *PHC2* |
| hsa-mir-605-5p | *ELP2* |
| hsa-mir-876-3p | *DSC3* |
| hsa-mir-605-5p | *UBQLN1* |
| hsa-mir-605-5p | *TES* |
| hsa-mir-876-3p | *NT5E* |
| hsa-mir-605-5p | *EGLN1* |
| hsa-mir-876-3p | *GLUL* |
| hsa-mir-876-3p | *DHX9* |
| hsa-mir-876-3p | *GPR55* |
| hsa-mir-605-5p | *REV1* |
| hsa-mir-605-5p | *MRPS9* |
| hsa-mir-876-3p | *DRAM1* |
| hsa-mir-605-5p | *SCRN1* |
| hsa-mir-605-5p | *CALCOCO2* |
| hsa-mir-876-3p | *TLN1* |
| hsa-mir-605-5p | *CPEB2* |
| hsa-mir-605-5p | *CPEB2* |
| hsa-mir-605-5p | *NUMA1* |
| hsa-mir-605-5p | *HADHB* |
| hsa-mir-876-3p | *ATIC* |
| hsa-mir-876-3p | *NAB1* |
| hsa-mir-876-3p | *ABI2* |
| hsa-mir-876-3p | *SECISBP2L* |
| hsa-mir-605-5p | *HNRNPD* |
| hsa-mir-605-5p | *KIF21A* |
| hsa-mir-605-5p | *ETNK1* |
| hsa-mir-605-5p | *TMTC3* |
| hsa-mir-876-3p | *LUM* |
| hsa-mir-605-5p | *SLC7A1* |
| hsa-mir-876-3p | *SLC7A1* |
| hsa-mir-876-3p | *LMBR1L* |
| hsa-mir-876-3p | *ANKRD52* |
| hsa-mir-605-5p | *ANP32A* |
| hsa-mir-605-5p | *ST8SIA2* |
| hsa-mir-605-5p | *ZCCHC14* |
| hsa-mir-605-5p | *RANBP10* |
| hsa-mir-605-5p | *PCTP* |
| hsa-mir-605-5p | *ESCO1* |
| hsa-mir-876-3p | *RNF165* |
| hsa-mir-876-3p | *SAMD1* |
| hsa-mir-876-3p | *APP* |
| hsa-mir-605-5p | *RERE* |
| hsa-mir-876-3p | *SERBP1* |
| hsa-mir-876-3p | *PTPRF* |
| hsa-mir-876-3p | *TMCO1* |
| hsa-mir-876-3p | *USP21* |
| hsa-mir-876-3p | *ABL2* |
| hsa-mir-605-5p | *MCL1* |
| hsa-mir-876-3p | *PI4KB* |
| hsa-mir-876-3p | *DYRK3* |
| hsa-mir-876-3p | *UBAP2L* |
| hsa-mir-605-5p | *FBXO28* |
| hsa-mir-876-3p | *ARF1* |
| hsa-mir-605-5p | *GUK1* |
| hsa-mir-605-5p | *PARP1* |
| hsa-mir-876-3p | *PARP1* |
| hsa-mir-876-3p | *TMEM177* |
| hsa-mir-605-5p | *SPOPL* |
| hsa-mir-605-5p | *CTDSPL* |
| hsa-mir-605-5p | *TMF1* |
| hsa-mir-876-3p | *COL8A1* |
| hsa-mir-876-3p | *PHLDB2* |
| hsa-mir-605-5p | *SLIT2* |
| hsa-mir-876-3p | *MYO10* |
| hsa-mir-605-5p | *BDP1* |
| hsa-mir-605-5p | *BDP1* |
| hsa-mir-605-5p | *BDP1* |
| hsa-mir-605-5p | *DDX46* |
| hsa-mir-876-3p | *DAAM2* |
| hsa-mir-605-5p | *MDH2* |
| hsa-mir-876-3p | *DIAPH2* |
| hsa-mir-605-5p | *SEC16A* |
| hsa-mir-876-3p | *NACC2* |
| hsa-mir-605-5p | *PARD3* |
| hsa-mir-605-5p | *HERC4* |
| hsa-mir-876-3p | *EIF4EBP2* |
| hsa-mir-605-5p | *CELF1* |
| hsa-mir-876-3p | *CELF1* |
| hsa-mir-876-3p | *TM7SF2* |
| hsa-mir-876-3p | *HMGA2* |
| hsa-mir-605-5p | *ARID5B* |
| hsa-mir-605-5p | *UEVLD* |
| hsa-mir-605-5p | *DLG5* |
| hsa-mir-605-5p | *DLG5* |
| hsa-mir-605-5p | *TEX30* |
| hsa-mir-605-5p | *MBIP* |
| hsa-mir-605-5p | *GUF1* |
| hsa-mir-605-5p | *PDE3B* |
| hsa-mir-876-3p | *MBNL1* |
| hsa-mir-605-5p | *PANK1* |
| hsa-mir-876-3p | *BCL2L11* |
| hsa-mir-605-5p | *HNRNPU* |
| hsa-mir-605-5p | *ASAP1* |
| hsa-mir-876-3p | *ASAP1* |
| hsa-mir-605-5p | *LPCAT1* |
| hsa-mir-605-5p | *LPCAT1* |
| hsa-mir-605-5p | *RMND5A* |
| hsa-mir-876-3p | *TRIP12* |
| hsa-mir-876-3p | *MSI2* |
| hsa-mir-605-5p | *LONRF1* |
| hsa-mir-605-5p | *LONRF1* |
| hsa-mir-605-5p | *ADAMTS5* |
| hsa-mir-605-5p | *AGPAT5* |
| hsa-mir-605-5p | *AGPAT5* |
| hsa-mir-605-5p | *PTDSS1* |
| hsa-mir-876-3p | *HK1* |
| hsa-mir-605-5p | *KAT6B* |
| hsa-mir-605-5p | *KAT6B* |
| hsa-mir-876-3p | *FBRS* |
| hsa-mir-876-3p | *BRPF1* |
| hsa-mir-605-5p | *TSC22D3* |
| hsa-mir-605-5p | *DNAH3* |
| hsa-mir-605-5p | *F11R* |
| hsa-mir-605-5p | *EPB41* |
| hsa-mir-876-3p | *SON* |
| hsa-mir-605-5p | *IGF2BP1* |
| hsa-mir-876-3p | *ADIPOR1* |
| hsa-mir-605-5p | *EFCAB14* |
| hsa-mir-876-3p | *ARHGAP35* |
| hsa-mir-876-3p | *DFFA* |
| hsa-mir-605-5p | *VMA21* |
| hsa-mir-605-5p | *PDXK* |
| hsa-mir-605-5p | *C21orf58* |
| hsa-mir-605-5p | *TAOK1* |
| hsa-mir-876-3p | *FAM189B* |
| hsa-mir-876-3p | *FAM189B* |
| hsa-mir-876-3p | *ITGA5* |
| hsa-mir-605-5p | *EIF4A1* |
| hsa-mir-605-5p | *SHANK2* |
| hsa-mir-876-3p | *ZNF281* |
| hsa-mir-605-5p | *RNF149* |
| hsa-mir-605-5p | *DCAF16* |
| hsa-mir-876-3p | *HIPK1* |
| hsa-mir-876-3p | *ATP1A1* |
| hsa-mir-605-5p | *CCNL1* |
| hsa-mir-605-5p | *PBRM1* |
| hsa-mir-605-5p | *RAD54L2* |
| hsa-mir-876-3p | *WDR82* |
| hsa-mir-876-3p | *CITED2* |
| hsa-mir-876-3p | *FASTK* |
| hsa-mir-605-5p | *INTS8* |
| hsa-mir-605-5p | *TMEM65* |
| hsa-mir-605-5p | *HNRNPK* |
| hsa-mir-605-5p | *GTF2A1* |
| hsa-mir-876-3p | *GTF2A1* |
| hsa-mir-605-5p | *ZCCHC24* |
| hsa-mir-605-5p | *CCDC186* |
| hsa-mir-876-3p | *TPP1* |
| hsa-mir-876-3p | *NAV2* |
| hsa-mir-876-3p | *MBD6* |
| hsa-mir-876-3p | *CERCAM* |
| hsa-mir-876-3p | *RRM1* |
| hsa-mir-605-5p | *GATAD2A* |
| hsa-mir-605-5p | *ANKRD11* |
| hsa-mir-876-3p | *KMT2D* |
| hsa-mir-876-3p | *LENG8* |
| hsa-mir-876-3p | *LENG8* |
| hsa-mir-876-3p | *LENG8* |
| hsa-mir-876-3p | *LENG8* |
| hsa-mir-876-3p | *EEF2* |
| hsa-mir-876-3p | *SRRM2* |
| hsa-mir-605-5p | *CTNNB1* |
| hsa-mir-876-3p | *CTNNB1* |
| hsa-mir-605-5p | *NUDT16L1* |
| hsa-mir-605-5p | *SETD5* |
| hsa-mir-876-3p | *STIP1* |
| hsa-mir-876-3p | *CPLX1* |
| hsa-mir-605-5p | *MECP2* |
| hsa-mir-876-3p | *SIN3A* |
| hsa-mir-605-5p | *SDC2* |
| hsa-mir-876-3p | *CLIC4* |
| hsa-mir-605-5p | *ZNF778* |
| hsa-mir-876-3p | *NIPA1* |
| hsa-mir-605-5p | *SLC30A1* |
| hsa-mir-876-3p | *HOXB9* |
| hsa-mir-605-5p | *NPTX1* |
| hsa-mir-605-5p | *PLRG1* |
| hsa-mir-876-3p | *CTPS1* |
| hsa-mir-876-3p | *ZNF217* |
| hsa-mir-605-5p | *ORMDL3* |
| hsa-mir-876-3p | *MANEA* |
| hsa-mir-876-3p | *HCFC1* |
| hsa-mir-605-5p | *ZMAT3* |
| hsa-mir-605-5p | *RPL38* |
| hsa-mir-876-3p | *VANGL1* |
| hsa-mir-876-3p | *SMARCC1* |
| hsa-mir-876-3p | *CBX2* |
| hsa-mir-605-5p | *KLHL15* |
| hsa-mir-605-5p | *RGMB* |
| hsa-mir-876-3p | *ZHX3* |
| hsa-mir-605-5p | *NR1D2* |
| hsa-mir-605-5p | *FZD4* |
| hsa-mir-876-3p | *MARCKSL1* |
| hsa-mir-605-5p | *EIF1AD* |
| hsa-mir-605-5p | *CCDC14* |
| hsa-mir-605-5p | *ZDHHC21* |
| hsa-mir-876-3p | *RIMS2* |
| hsa-mir-876-3p | *DIRAS1* |
| hsa-mir-605-5p | *NFATC2IP* |
| hsa-mir-605-5p | *POLE* |
| hsa-mir-605-5p | *RPLP2* |
| hsa-mir-876-3p | *JUN* |
| hsa-mir-876-3p | *CD151* |
| hsa-mir-605-5p | *GRB2* |
| hsa-mir-605-5p | *ZBTB41* |
| hsa-mir-605-5p | *C2orf73* |
| hsa-mir-876-3p | *NDUFAF3* |
| hsa-mir-876-3p | *ZNF518B* |
| hsa-mir-605-5p | *ZNF354B* |
| hsa-mir-876-3p | *ZBTB7A* |
| hsa-mir-876-3p | *MAGED1* |
| hsa-mir-876-3p | *CDH4* |
| hsa-mir-876-3p | *CDH4* |
| hsa-mir-605-5p | *FJX1* |
| hsa-mir-876-3p | *MYADM* |
| hsa-mir-876-3p | *ZNF784* |
| hsa-mir-605-5p | *PLD5* |
| hsa-mir-605-5p | *SKIDA1* |
| hsa-mir-605-5p | *DHTKD1* |
| hsa-mir-605-5p | *C5orf30* |
| hsa-mir-876-3p | *RGMA* |
| hsa-mir-605-5p | *C1S* |
| hsa-mir-876-3p | *SATB1* |
| hsa-mir-876-3p | *NGRN* |
| hsa-mir-605-5p | *RPL35A* |
| hsa-mir-605-5p | *CADM1* |
| hsa-mir-605-5p | *LYSMD4* |
| hsa-mir-605-5p | *BCOR* |
| hsa-mir-605-5p | *PRR14L* |
| hsa-mir-876-3p | *ZNRF3* |
| hsa-mir-876-3p | *CBX6* |
| hsa-mir-876-3p | *ZNF703* |
| hsa-mir-876-3p | *ACTG1* |
| hsa-mir-876-3p | *NR2C2AP* |
| hsa-mir-876-3p | *SDR42E1* |
| hsa-mir-876-3p | *ROBO2* |
| hsa-mir-876-3p | *BRF1* |
| hsa-mir-605-5p | *ZBTB37* |
| hsa-mir-605-5p | *ARL15* |
| hsa-mir-876-3p | *ATP6V0A2* |
| hsa-mir-876-3p | *SP1* |
| hsa-mir-605-5p | *BRWD1* |
| hsa-mir-605-5p | *ANKFY1* |
| hsa-mir-876-3p | *DMWD* |
| hsa-mir-605-5p | *IKZF1* |
| hsa-mir-605-5p | *NAT8L* |
| hsa-mir-605-5p | *PPP1CC* |
| hsa-mir-876-3p | *PPP1CC* |
| hsa-mir-605-5p | *HEXIM1* |
| hsa-mir-605-5p | *TET3* |
| hsa-mir-605-5p | *FANCM* |
| hsa-mir-605-5p | *NCR3LG1* |
| hsa-mir-876-3p | *TUBB4B* |
| hsa-mir-876-3p | *COL25A1* |
| hsa-mir-605-5p | *NBR1* |
| hsa-mir-876-3p | *MTF1* |
| hsa-mir-605-5p | *PLEKHG4* |
| hsa-mir-605-5p | *GREB1* |
| hsa-mir-876-3p | *TUBB* |
| hsa-mir-876-3p | *TUBB* |
| hsa-mir-876-3p | *TUBB* |
| hsa-mir-876-3p | *TUBB* |
| hsa-mir-876-3p | *TUBB* |
| hsa-mir-876-3p | *TUBB* |
| hsa-mir-876-3p | *TUBB* |
| hsa-mir-876-3p | *TUBB* |
| hsa-mir-876-3p | *MME* |
| hsa-mir-605-5p | *NF1* |
| hsa-mir-605-5p | *VKORC1L1* |
| hsa-mir-876-3p | *PDLIM7* |
| hsa-mir-876-3p | *SLC39A10* |
| hsa-mir-876-3p | *MAFG* |
| hsa-mir-876-3p | *ARRDC1* |
| hsa-mir-876-3p | *IGF2R* |
| hsa-mir-876-3p | *PCBP2* |
| hsa-mir-876-3p | *SND1* |
| hsa-mir-605-5p | *MYO5A* |
| hsa-mir-605-5p | *PSAP* |
| hsa-mir-876-3p | *VPS13A* |
| hsa-mir-605-5p | *SIRPA* |
| hsa-mir-876-3p | *NRARP* |
| hsa-mir-605-5p | *CALM1* |
| hsa-mir-876-3p | *SMOC1* |
| hsa-mir-876-3p | *HMGN2* |
| hsa-mir-876-3p | *RBM20* |
| hsa-mir-876-3p | *BMPR2* |
| hsa-mir-876-3p | *BRD2* |
| hsa-mir-876-3p | *BRD2* |
| hsa-mir-876-3p | *BRD2* |
| hsa-mir-876-3p | *BRD2* |
| hsa-mir-876-3p | *BRD2* |
| hsa-mir-876-3p | *BRD2* |
| hsa-mir-876-3p | *BRD2* |
| hsa-mir-605-5p | *BAG6* |
| hsa-mir-605-5p | *BAG6* |
| hsa-mir-605-5p | *BAG6* |
| hsa-mir-605-5p | *BAG6* |
| hsa-mir-605-5p | *BAG6* |
| hsa-mir-605-5p | *BAG6* |
| hsa-mir-605-5p | *BAG6* |
| hsa-mir-876-3p | *PRRC2A* |
| hsa-mir-876-3p | *PRRC2A* |
| hsa-mir-876-3p | *PRRC2A* |
| hsa-mir-876-3p | *PRRC2A* |
| hsa-mir-876-3p | *PRRC2A* |
| hsa-mir-876-3p | *PRRC2A* |
| hsa-mir-876-3p | *PRRC2A* |
| hsa-mir-876-3p | *PPP1R10* |
| hsa-mir-876-3p | *PPP1R10* |
| hsa-mir-876-3p | *PPP1R10* |
| hsa-mir-876-3p | *PPP1R10* |
| hsa-mir-876-3p | *PPP1R10* |
| hsa-mir-876-3p | *PPP1R10* |
| hsa-mir-876-3p | *PPP1R10* |
| hsa-mir-605-5p | *IPO7* |
| hsa-mir-605-5p | *CNEP1R1* |
| hsa-mir-605-5p | *CRYZL1* |
| hsa-mir-876-3p | *VGLL3* |
| hsa-mir-605-5p | *TRIM71* |
| hsa-mir-605-5p | *SFT2D2* |
| hsa-mir-876-3p | *LCAT* |
| hsa-mir-605-5p | *DDX3X* |
| hsa-mir-876-3p | *FNIP1* |
| hsa-mir-876-3p | *PPP1R3E* |
| hsa-mir-876-3p | *PCDHGC3* |
| hsa-mir-605-5p | *ARPIN* |
| hsa-mir-605-5p | *ZBED6* |
| hsa-mir-605-5p | *PCDHGA4* |
| hsa-mir-605-5p | *BAHCC1* |
| hsa-mir-876-3p | *SYNRG* |
| hsa-mir-876-3p | *SYNRG* |
| hsa-mir-605-5p | *PIP4K2B* |
| hsa-mir-605-5p | *PIP4K2B* |
| hsa-mir-876-3p | *DACH1* |

**Table S2**. The node pairs of PPI network provided by STRING database.

| node1 | node2 |
| --- | --- |
| CDK2 | CCND1 |
| RPL23 | EEF2 |
| EIF4G1 | EIF4A1 |
| MDM4 | TP53 |
| SF3B2 | SF3B3 |
| RPL14 | RPL38 |
| RPLP2 | EEF2 |
| RPL35A | EEF2 |
| SLIT2 | ROBO1 |
| RPLP2 | RPL38 |
| GRB2 | CBL |
| CDK6 | CCND1 |
| RPL35A | RPLP2 |
| CTNNB1 | BCL9 |
| RPL35A | RPL14 |
| RPL23 | RPL14 |
| PLRG1 | RBM22 |
| RPL14 | RPLP2 |
| TPX2 | AURKA |
| RPL35A | RPL38 |
| TP53 | MDM2 |
| ALYREF | DDX39B |
| RPL23 | RPLP2 |
| RPL23 | RPL35A |
| SRSF7 | SRSF9 |
| RPL23 | RPL38 |
| RPL38 | EEF2 |
| RPL14 | EEF2 |
| TUBB4B | TUBB |
| MDM4 | MDM2 |
| BCL2L11 | MCL1 |
| HNRNPK | HNRNPA2B1 |
| TP53 | CDK2 |
| ARID1A | SMARCC1 |
| BRF1 | BDP1 |
| SNRPB | SF3B3 |
| RPL23 | ETF1 |
| ETF1 | RPL38 |
| PBRM1 | SMARCC1 |
| SRRM2 | RBM22 |
| PLRG1 | SF3B3 |
| PLRG1 | SRRM2 |
| GRB2 | SOS2 |
| ARID1B | SMARCC1 |
| ALYREF | HNRNPA2B1 |
| SNRPB | RBM22 |
| HIPK2 | TP53 |
| ALYREF | SRSF7 |
| SLIT2 | ROBO2 |
| ALYREF | HNRNPK |
| PSMD7 | PSMD10 |
| CRKL | CBL |
| CTNNB1 | CCND1 |
| HNRNPK | HNRNPU |
| TP53 | AURKA |
| ARID1B | ARID1A |
| PLRG1 | SNRPB |
| ALYREF | DDX39A |
| RNF2 | PHC2 |
| HNRNPA2B1 | HNRNPU |
| HNRNPK | SRSF7 |
| AKT2 | EGLN1 |
| HNRNPA2B1 | SRSF7 |
| KDM1A | TP53 |
| ACVR2B | INHBA |
| BRPF1 | KAT6B |
| RPL14 | ETF1 |
| JUN | ATF2 |
| SEC16A | SEC24D |
| HNRNPK | DHX9 |
| SNRPB | SRRM2 |
| CENPQ | CENPH |
| JUN | TP53 |
| DDX39B | SRSF7 |
| RPL35A | ETF1 |
| CSNK1A1 | CTNNB1 |
| CTNNB1 | AMER1 |
| RNF2 | CBX2 |
| HIST1H3D | H3F3B |
| DROSHA | DDX5 |
| DHX9 | HNRNPA2B1 |
| FGFR2 | GRB2 |
| PLRG1 | SF3B2 |
| ACTG1 | ACTB |
| HNRNPA2B1 | HNRNPD |
| SF3B3 | SRRM2 |
| GTF2A1 | TAF2 |
| CBX6 | RNF2 |
| SNRPB | SF3B2 |
| SP1 | TP53 |
| ARF1 | ARF5 |
| POLE | MSH2 |
| DYRK2 | TP53 |
| DHX9 | DDX5 |
| EIF4G1 | NCBP2 |
| HNRNPK | HNRNPD |
| PPP1CC | WDR82 |
| JUN | CCND1 |
| DDX46 | SF3B2 |
| DROSHA | TP53 |
| MCL1 | TP53 |
| ACTB | ACTC1 |
| F11R | TJP1 |
| BBC3 | MCL1 |
| SMC1A | ESCO1 |
| ALYREF | DHX9 |
| STX16 | VAMP7 |
| HNRNPD | HNRNPU |
| TP53 | C12orf5 |
| TLN1 | PXN |
| VEGFA | JUN |
| MECP2 | SIN3A |
| ANP32A | XPO1 |
| ARF1 | ASAP1 |
| NANOS1 | CNOT1 |
| DDX39B | DDX39A |
| HNRNPA2B1 | SRSF9 |
| SF3B2 | SRRM2 |
| JUN | CTNNB1 |
| SF3B3 | RBM22 |
| TP53 | MSH2 |
| TLN1 | ITGA5 |
| CRKL | PXN |
| ZFP36 | XRN1 |
| DICER1 | DHX9 |
| HNRNPK | DDX5 |
| VEGFA | CTNNB1 |
| DHX9 | HNRNPU |
| ZNF207 | BUB3 |
| TP53 | DDX5 |
| PCBP2 | HNRNPA2B1 |
| KMT2A | HCFC1 |
| BBC3 | TP53 |
| HNRNPK | PCBP2 |
| TSC22D3 | SGK1 |
| PARD3 | F11R |
| SRRM2 | RBM25 |
| CAMK2B | CALM1 |
| SF3B2 | RBM22 |
| YBX1 | HNRNPD |
| CBX2 | PHC2 |
| DDX39B | NCBP2 |
| TP53 | PSMD10 |
| PCBP2 | HNRNPD |
| CTNNB1 | TJP1 |
| EEF2 | EIF4A1 |
| JUN | SP1 |
| ABI2 | WASL |
| SIN3A | TP53 |
| PAFAH1B1 | YWHAE |
| ACTG1 | WASL |
| CBX6 | PHC2 |
| E2F8 | TP53 |
| CDK2 | CDK6 |
| SRSF7 | HNRNPU |
| ILF3 | HNRNPA2B1 |
| ALYREF | HNRNPU |
| KDM1A | HIST1H3D |
| PPP1R10 | PPP1CC |
| DSN1 | CENPH |
| AKT2 | TP53 |
| EZH1 | RNF2 |
| WEE1 | CDK2 |
| ALYREF | NCBP2 |
| SRRM2 | ACIN1 |
| HMGA2 | TP53 |
| DHCR24 | TM7SF2 |
| YBX1 | HNRNPA2B1 |
| RPL23 | SERBP1 |
| LDLR | FZD4 |
| MTRF1L | MRPS9 |
| WBP11 | DDX5 |
| ARF1 | PI4KB |
| RNF4 | MDC1 |
| DDX39B | HNRNPA2B1 |
| EIF4G1 | CNOT1 |
| NF1 | SDC2 |
| ILF3 | HNRNPK |
| CDK2 | MDM2 |
| RANBP10 | RMND5A |
| RPL35A | EIF4A1 |
| ALYREF | DDX5 |
| HNRNPK | YBX1 |
| ARF1 | PLEKHA3 |
| HCFC1 | WDR82 |
| EZH1 | CBX6 |
| RPL23 | MDM2 |
| APP | GAPDH |
| YBX1 | PCBP2 |
| NCBP2 | SF3B2 |
| SIN3A | BRMS1L |
| HAUS5 | NUMA1 |
| HNRNPA2B1 | DDX5 |
| HNRNPK | SRSF9 |
| PCBP2 | SF3B2 |
| YBX1 | DHX9 |
| NUMA1 | TPX2 |
| MDC1 | SMC1A |
| TP53 | CCND1 |
| SIN3A | RUNX1 |
| RUNX1 | CDK6 |
| NCBP2 | SF3B3 |
| YBX1 | HNRNPU |
| TRIP12 | UBE2A |
| HIPK2 | RUNX1 |
| PCBP2 | SRSF7 |
| LATS1 | STK4 |
| ATF2 | CCND1 |
| RPL23 | MRPS9 |
| EZH1 | CBX2 |
| YBX1 | SRSF9 |
| CTNNB1 | SOX4 |
| PLRG1 | DDX46 |
| HNRNPU | DDX5 |
| KDM1A | H3F3B |
| RPL14 | EIF4A1 |
| DDX39B | HNRNPK |
| NUMA1 | AURKA |
| ALYREF | SNRPB |
| DDX46 | NCBP2 |
| ACTB | WASL |
| DDX46 | SF3B3 |
| TRIP12 | FBXL19 |
| PIP4K2B | PI4KB |
| DHX9 | SRSF7 |
| TUBB | YWHAE |
| ABL2 | CRKL |
| CTNNB1 | KMT2D |
| RPL23 | EIF4A1 |
| CYLD | STAT1 |
| DSN1 | BUB3 |
| TLN1 | ITGA2B |
| HNRNPA2B1 | SF3B3 |
| TLN1 | ACTN4 |
| HNRNPD | SRSF9 |
| RNF2 | TP53 |
| YBX1 | IGF2BP1 |
| ALYREF | PCBP2 |
| SF3B2 | RBM25 |
| DDX39B | SRSF9 |
| ALYREF | YBX1 |
| CNOT1 | EIF4A1 |
| HNRNPK | SF3B3 |
| SF3B2 | SRSF9 |
| UBE2A | MDM2 |
| PXN | ACTN4 |
| DHX9 | SF3B3 |
| XPO1 | NUP160 |
| H3F3B | BCL9 |
| UBE2A | TP53 |
| EIF3L | EIF4G1 |
| SRSF7 | HNRNPD |
| ETF1 | RPLP2 |
| H3F3C | BCL9 |
| JUN | ACTB |
| SNRPB | DHX9 |
| SEC24D | CANX |
| RPLP2 | EIF4A1 |
| BUB3 | CENPH |
| CSNK1A1 | TP53 |
| HIPK1 | TP53 |
| SNRPB | HNRNPA2B1 |
| TP53 | YWHAE |
| TUBB4B | YWHAE |
| SRSF7 | SF3B3 |
| DDX46 | RBM25 |
| BRD2 | CCND1 |
| POU2F1 | SP1 |
| ACTB | GAPDH |
| ALYREF | SRSF9 |
| NF1 | ARID1A |
| PLRG1 | DHX35 |
| SIN3A | GATAD2A |
| KDM1A | H3F3C |
| AKT2 | CTNNB1 |
| FGFR2 | CBL |
| HEPH | FECH |
| RNF4 | UBE2A |
| BUB3 | SMC1A |
| DSN1 | CENPQ |
| UBE2F | TRIP12 |
| AKT2 | MDM2 |
| EGLN1 | LIMD1 |
| VEGFA | PXN |
| SNRPB | NCBP2 |
| JUN | ATN1 |
| SNRPB | HNRNPD |
| PCBP2 | HNRNPU |
| NCBP2 | DDX39A |
| IGF2BP1 | HNRNPU |
| EIF3L | EIF4A1 |
| SRRM2 | DDX5 |
| ACTB | TUBB4B |
| DYRK2 | MDM2 |
| KDM1A | RNF2 |
| DHX9 | SF3B2 |
| RPL35A | SERBP1 |
| KMT2A | RUNX1 |
| BRPF1 | HIST1H3D |
| HNRNPA2B1 | SRRM2 |
| CACYBP | CTNNB1 |
| DHX9 | PCBP2 |
| ANAPC16 | BUB3 |
| VEGFA | GRB2 |
| EIF3L | RPL35A |
| ZNRF3 | FZD4 |
| YBX1 | DDX5 |
| DHX9 | SRRM2 |
| DDX46 | SRRM2 |
| LATS1 | LIMD1 |
| SDC2 | NDST3 |
| HNRNPU | SRSF9 |
| FECH | UROD |
| SNRPB | SRSF9 |
| FECH | ABCB7 |
| IGF2R | CPD |
| KDM1A | ATN1 |
| SRSF7 | DDX39A |
| SNAP91 | WASL |
| YBX1 | SF3B2 |
| DHX9 | IGF2BP1 |
| ARID1A | KMT2D |
| PLRG1 | ALYREF |
| KMT2A | KMT2D |
| SERP1 | SEC63 |
| ALYREF | SRRM2 |
| KIAA0226 | PIK3C3 |
| GRB2 | IRS4 |
| RUNX1 | CCND1 |
| DDX46 | RBM22 |
| SEC24D | CNIH1 |
| GRB2 | CRKL |
| SNRPB | HNRNPU |
| SF3B2 | WBP11 |
| CELF1 | MBNL1 |
| PLRG1 | HNRNPA2B1 |
| RNF2 | KDM6B |
| DLG1 | CTNNB1 |
| SRRM2 | SRSF9 |
| PCBP2 | SRSF9 |
| POU2F1 | NF1 |
| HIST1H3D | BCL9 |
| VEGFA | APP |
| DDX46 | DHX9 |
| DHX9 | HNRNPD |
| MYO5A | EXOC2 |
| MDC1 | CDK2 |
| BUB3 | PPP1CC |
| NCBP2 | RBM22 |
| PLRG1 | NCBP2 |
| NCBP2 | DDX5 |
| ROBO1 | ROBO2 |
| PAFAH1B1 | DNAH3 |
| HNRNPD | SF3B3 |
| SERBP1 | RPL38 |
| EZH1 | PHC2 |
| ABL2 | ROBO1 |
| HNRNPD | DDX5 |
| BUB3 | TAOK1 |
| ARID1A | CCND1 |
| MBD6 | HCFC1 |
| PLRG1 | HNRNPD |
| EIF3L | RPL14 |
| EIF4G1 | ETF1 |
| KMT2A | SIN3A |
| DDX46 | SRSF7 |
| CBX2 | KDM6B |
| XPO1 | PPP1CC |
| XPO1 | BUB3 |
| PCBP2 | DDX5 |
| CDH4 | CTNNB1 |
| ALYREF | HNRNPD |
| SF3B3 | RBM25 |
| PAFAH1B1 | NUMA1 |
| ABL2 | CAP2 |
| HNRNPA2B1 | SF3B2 |
| ARF1 | IGF2R |
| SF3B3 | DDX5 |
| DDX39A | SRSF9 |
| TP53 | ATF2 |
| PI4KB | PIK3C3 |
| COPS7B | ASB1 |
| HNRNPA2B1 | RBM22 |
| ACTG1 | ACTN4 |
| RPL38 | EIF4A1 |
| MYO5A | RAB14 |
| SNRPB | SRSF7 |
| BBC3 | BCL2L11 |
| APP | CX3CL1 |
| HNRNPD | RBM22 |
| SF3B2 | HNRNPU |
| CAMK2B | DLG1 |
| DHX9 | RBM22 |
| PAFAH1B1 | AURKA |
| ENTPD4 | ATIC |
| BMPR2 | RGMB |
| HNRNPD | SRRM2 |
| KMT2A | RNF2 |
| ARF1 | VAMP7 |
| SRRM2 | HNRNPU |
| TPX2 | TP53 |
| JUN | POU2F1 |
| PLRG1 | DHX9 |
| DICER1 | TRIM71 |
| KDM1A | GATAD2A |
| RAB14 | EXOC2 |
| DHX9 | SRSF9 |
| ACTB | SMARCC1 |
| NCL | S100A11 |
| PRKAA1 | PIK3C3 |
| NF1 | ATF2 |
| CCNL1 | CDK6 |
| SNRPB | DDX5 |
| PAFAH1B1 | TPX2 |
| RNF4 | TRIP12 |
| RUNX1 | ITGA2B |
| ALYREF | SF3B3 |
| LDLR | IGF2R |
| CDK2 | AURKA |
| STX16 | IGF2R |
| FGFR2 | JUN |
| DDX5 | RBM22 |
| SNRPB | HNRNPK |
| SF3B3 | SRSF9 |
| NCBP2 | SRRM2 |
| NF1 | ACTB |
| DDX46 | HNRNPA2B1 |
| SNAP91 | VAMP7 |
| HNRNPK | SF3B2 |
| SGK1 | MDM2 |
| DDX46 | SNRPB |
| NCBP2 | HNRNPD |
| SF3B2 | HNRNPD |
| CBX6 | KDM1A |
| FBXL16 | FBXL19 |
| RPL23 | EIF4G1 |
| ALYREF | DDX46 |
| POU2F1 | ATF2 |
| CDK2 | PSMD7 |
| POLE | CDK2 |
| RUNX1 | SMARCC1 |
| TRIP12 | FBXO21 |
| CCND1 | PSMD7 |
| HNRNPK | SRRM2 |
| SF3B2 | DDX5 |
| ACTB | ACTN4 |
| SIN3A | ZNF217 |
| DHX9 | NCBP2 |
| CCND1 | PSMD10 |
| TUBB | AURKA |
| TMF1 | STX16 |
| PAFAH1B1 | BUB3 |
| RAB5B | VAMP7 |
| XPO1 | CTNNB1 |
| EPHA4 | CBL |
| FBXL19 | UBE2A |
| EPB41 | SDC2 |
| PRKAA1 | TP53 |
| DDX46 | DDX5 |
| EIF4G1 | RPL38 |
| PAFAH1B1 | PPP1CC |
| SRSF7 | DDX5 |
| SNRPB | WBP11 |
| SF3B3 | HNRNPU |
| ALYREF | DDX3X |
| CBX6 | CBX2 |
| NCBP2 | SRSF7 |
| HNRNPA2B1 | NCBP2 |
| PAFAH1B1 | TUBB |
| DDX46 | PCBP2 |
| TP53 | GAPDH |
| SIN3A | KMT2D |
| ABL2 | SLIT2 |
| RNF4 | NFATC2IP |
| SRSF7 | SRRM2 |
| ACTG1 | MYO10 |
| EIF4G1 | DDX3X |
| HAUS5 | AURKA |
| LPGAT1 | LPCAT1 |
| XPO1 | YWHAE |
| NCBP2 | WBP11 |
| EIF3L | RPLP2 |
| PBRM1 | CCND1 |
| DSC3 | DSG2 |
| JUN | MSH2 |
| CDK2 | PSMD10 |
| SNRPB | RBM25 |
| PBRM1 | ARID1A |
| YBX1 | SF3B3 |
| GRB2 | TNK2 |
| MDM4 | DYRK2 |
| CBX6 | KDM6B |
| SRSF9 | DDX5 |
| JUN | SMARCC1 |
| HERC4 | TRIP12 |
| NUP160 | CENPQ |
| AKT2 | PDE3B |
| SLIT2 | CAP2 |
| ROBO1 | CTNNB1 |
| CTNNB1 | YWHAE |
| RGMA | BMPR2 |
| GREB1 | ZNF217 |
| VEGFA | ACTN4 |
| DDX46 | HNRNPD |
| IGF2R | VAMP7 |
| MYO10 | ACTB |
| SNRPB | YBX1 |
| CNOT1 | TP53 |
| ROBO1 | SOS2 |
| UBE2A | FBXO21 |
| MORF4L2 | ACTB |
| NCBP2 | SRSF9 |
| KDM1A | MSH2 |
| DSN1 | PPP1CC |
| FGFR2 | STAT1 |
| TRIP12 | ASB1 |
| PPP1R3E | PPP1CC |
| FGFR2 | SDC2 |
| PXN | ITGA2B |
| NT5E | ATIC |
| DACH1 | SIX1 |
| RBBP6 | MDM2 |
| ALYREF | SF3B2 |
| ZFP36 | CNOT1 |
| ACTB | ARID1A |
| KDM1A | JUN |
| RAB14 | RAB5B |
| MYO9B | MYO5A |
| JUN | KDM6B |
| YBX1 | SRRM2 |
| TUBB | TPX2 |
| RAB14 | YWHAE |
| NUP160 | CENPH |
| APP | TNC |
| VEGFA | CBL |
| PTDSS1 | LCAT |
| DDX39B | NUP160 |
| BUB3 | PSMD10 |
| PPP1CC | SMC1A |
| BLOC1S3 | VAMP7 |
| ACTB | TJP1 |
| DLG1 | MDM2 |
| GUK1 | RRM1 |
| KMT2D | BCL9 |
| SLIT2 | SOS2 |
| HNRNPK | RNF2 |
| ALYREF | ACIN1 |
| GAN | KLHL42 |
| TRIM71 | UBE2A |
| PCBP2 | WBP11 |
| RRM1 | MSH2 |
| ARID1B | CCND1 |
| EDN3 | APP |
| CACNA1E | CALM1 |
| PCBP2 | SF3B3 |
| BUB3 | CENPQ |
| FBXL16 | UBE2A |
| CBX2 | TP53 |
| CRKL | TLN1 |
| UBAP2L | PHC2 |
| DEK | MYBBP1A |
| HERC4 | UBE2A |
| HNRNPK | NCBP2 |
| ILF3 | DDX39B |
| EIF3L | RPL23 |
| VEGFA | TP53 |
| POLE | RRM1 |
| RNF2 | HCFC1 |
| ATN1 | TP53 |
| ETF1 | NCBP2 |
| TUBB4B | AURKA |
| COL25A1 | COL8A1 |
| MYO5A | AKT2 |
| UBE2F | UBE2A |
| VAMP7 | WASL |
| TP53 | PHC2 |
| EIF4G1 | RPLP2 |
| KDM1A | CBX2 |
| DDX46 | YBX1 |
| CRTC2 | YWHAE |
| UBE2A | ASB1 |
| PAFAH1B1 | CAPZA2 |
| CTNNB1 | PSMD10 |
| HNRNPK | WBP11 |
| YBX1 | SRSF7 |
| PLRG1 | SRSF7 |
| XPO1 | DDX3X |
| PCBP2 | NCBP2 |
| HNRNPK | CPSF6 |
| ALYREF | NUP160 |
| NUP160 | NCBP2 |
| TPD52 | VAMP7 |
| DDX46 | HNRNPK |
| SMARCC1 | CCND1 |
| KDM1A | SPEN |
| ARID1A | RUNX1 |
| LDLR | SNAP91 |
| BRPF1 | TP53 |
| CSNK1A1 | YWHAE |
| ARF1 | KIF21A |
| ARID1B | RUNX1 |
| ABL2 | ABI2 |
| NCBP2 | HNRNPU |
| SNRPB | PCBP2 |
| PSMD10 | AURKA |
| LIMD1 | PSMD10 |
| TNKS2 | PSMD7 |
| PLRG1 | DDX5 |
| CEP55 | AURKA |
| BMPR2 | ACVR2B |
| SMC1A | CENPH |
| ARF1 | CAPZA2 |
| NUP160 | SRSF7 |
| EIF3L | RPL38 |
| CSNK1A1 | AMER1 |
| PAFAH1B1 | TUBB4B |
| ILF3 | DHX9 |
| PHC2 | KDM6B |
| JUN | ARID1A |
| EIF4G1 | RPL14 |
| ATN1 | PHC2 |
| HOXB2 | KMT2D |
| CBL | WASL |
| UBE2F | ASB1 |
| GATAD2A | UBTF |
| PLRG1 | HNRNPK |
| RAB5B | WASL |
| SNAP91 | IGF2R |
| PCBP2 | SRRM2 |
| DDX46 | SRSF9 |
| RPL35A | EIF4G1 |
| TUBB4B | TPX2 |
| PLRG1 | HNRNPU |
| NUP160 | BUB3 |
| VAPA | LPCAT1 |
| ACTB | ATF2 |
| XPO1 | SMC1A |
| CRKL | ACTN4 |
| DDX46 | HNRNPU |
| NUP160 | SMC1A |
| FZD4 | RAB5B |
| SRSF7 | SF3B2 |
| GATAD2A | CBX2 |
| MPRIP | TLN1 |
| DYNLRB1 | IFT81 |
| TAF2 | TP53 |
| POU2F1 | CCND1 |
| PLRG1 | WBP11 |
| SNAP91 | RAB5B |
| TRIP12 | RBBP6 |
| MYBBP1A | ACTB |
| DSN1 | NUP160 |
| ZNF217 | GATAD2A |
| FBXL16 | TRIP12 |
| RNF2 | RUNX1 |
| PARP1 | POLE |
| SDC2 | APP |
| ACTB | ABI2 |
| JUN | KMT2D |
| ACTG1 | ABI2 |
| ABI2 | PXN |
| STAT1 | SDC2 |
| RAB5B | PIK3C3 |
| PSAP | APP |
| KIF21A | ARF5 |
| UBE2F | FBXL19 |
| TAF2 | USP22 |
| HERC4 | FBXL19 |
| RGMA | RGMB |
| TRIM71 | TRIM21 |
| GATAD2A | ATN1 |
| MYO9B | SLIT2 |
| TP53 | PSMD7 |
| SIN3A | HCFC1 |
| UBE2F | FBXL16 |
| GTF2A1 | SP1 |
| PAFAH1B1 | TAOK1 |
| RNF4 | TRIM21 |
| NUP160 | DDX39A |
| RAB14 | FRMPD3 |
| DCAF7 | DYRK2 |
| GAN | UBE2A |
| CSNK1A1 | PSMD10 |
| APP | ACTN4 |
| NUP160 | PPP1CC |
| JUN | GATAD2A |
| DSN1 | SMC1A |
| NCBP2 | RBM25 |
| DDX46 | WBP11 |
| WBP11 | SRSF9 |
| DHX9 | RPL38 |
| GATAD2A | TP53 |
| SRRM2 | WBP11 |
| FBXL19 | RBBP6 |
| SDC2 | TNC |
| IGF2R | WASL |
| ALYREF | ILF3 |
| IP6K1 | IP6K2 |
| NF1 | PSMD10 |
| CBX6 | TP53 |
| UBE2F | HERC4 |
| GAN | TRIM21 |
| INTS8 | NCBP2 |
| TRIP12 | TRIM21 |
| NCBP2 | RPL38 |
| GTF2A1 | NCBP2 |
| RBFOX2 | NCBP2 |
| PLRG1 | PCBP2 |
| ROBO1 | CAP2 |
| TRIP12 | TRIM71 |
| GRB2 | SDC2 |
| DSN1 | XPO1 |
| JUN | SDC2 |
| PLRG1 | SRSF9 |
| PTPRF | CTNNB1 |
| PAFAH1B1 | SMC1A |
| GTF2A1 | ACTB |
| GTF2A1 | SMARCC1 |
| BMPR2 | ATF2 |
| SRSF7 | WBP11 |
| XPO1 | TAOK1 |
| HERC4 | ASB1 |
| PSMD7 | AURKA |
| XPO1 | PAFAH1B1 |
| RPL14 | NCBP2 |
| NUP160 | SRSF9 |
| HNRNPK | NCL |
| NF1 | SMARCC1 |
| UBE2A | MGRN1 |
| BCL2L11 | ITGA2B |
| KMT2A | ITGA2B |
| UBE2A | TRIM21 |
| FBXL16 | HERC4 |
| CAPZA2 | ARF5 |
| TRIM71 | ASB1 |
| FBXL19 | MGRN1 |
| VAMP7 | CPD |
| RPL23 | NCBP2 |
| PPP1CC | CENPH |
| NUMA1 | YWHAE |
| SRSF7 | RBM22 |
| ASB1 | TRIM21 |
| CNEP1R1 | PPP1CC |
| FBXL19 | ASB1 |
| PLRG1 | YBX1 |
| KLHL42 | UBE2A |
| NUMA1 | TUBB4B |
| INTS8 | SP1 |
| BUB3 | PSMD7 |
| KMT2D | RUNX1 |
| CENPQ | SMC1A |
| TRIM71 | FBXL19 |
| SF3B3 | WBP11 |
| SEC16A | CNIH1 |
| XPO1 | CENPH |
| YWHAE | AURKA |
| RNF4 | FBXL19 |
| PSAP | VAPA |
| PTDSS1 | LPCAT1 |
| ALYREF | WBP11 |
| DHX9 | WBP11 |
| CEBPD | THRAP3 |
| CBX6 | JUN |
| VEGFA | ARF1 |
| YBX1 | NCBP2 |
| HERC4 | RBBP6 |
| XPO1 | CENPQ |
| FBXL19 | TRIM21 |
| GAN | TRIP12 |
| HAUS5 | TPX2 |
| ARF1 | TPD52 |
| EPHB4 | EPHA4 |
| SP1 | NCBP2 |
| GTF2A1 | POU2F1 |
| PSAP | CX3CL1 |
| SNRPB | NUP160 |
| ARF1 | CPD |
| SRSF9 | RBM22 |
| GTF2A1 | DDX5 |
| LDLR | VAMP7 |
| NUMA1 | TUBB |
| HNRNPK | RBM22 |
| JUN | CBX2 |
| SNAP91 | CBL |
| GAN | FBXL19 |
| GAN | HERC4 |
| HNRNPU | RBM22 |
| POU2F1 | BDP1 |
| FZD4 | CBL |
| CBX6 | ATN1 |
| JUN | RNF2 |
| DEK | ACTB |
| ORMDL3 | MLEC |
| ZNFX1 | SIN3A |
| BRD2 | RUNX1 |
| LDLR | WASL |
| CAMK2B | STAT1 |
| RUNX1 | PHC2 |
| HNRNPU | WBP11 |
| GTF2A1 | INTS8 |
| DSN1 | PAFAH1B1 |
| RAB5B | CBL |
| GAN | COPS7B |
| TNKS2 | PSMD10 |
| RNF2 | GATAD2A |
| PPP1CC | TAOK1 |
| LONRF1 | FBXL16 |
| PPP1CC | CENPQ |
| RNF4 | TRIM71 |
| TULP4 | ASB1 |
| LONRF1 | ASB1 |
| UBE2F | RNF4 |
| HAUS5 | TUBB |
| LONRF1 | TRIM21 |
| TPD52 | IGF2R |
| FBXL16 | ASB1 |
| NCBP2 | RPLP2 |
| SPCS3 | RPL23 |
| SMC1A | TAOK1 |
| LONRF1 | TRIP12 |
| SPOP | PSMD7 |
| YBX1 | WBP11 |
| CSNK1A1 | PSMD7 |
| RNF4 | LONRF1 |
| HERC4 | TRIM21 |
| SND1 | TLN1 |
| GATAD2A | ATF2 |
| WBP11 | RBM22 |
| KDM1A | ATF2 |
| FBXL19 | TULP4 |
| DCAF10 | COPS7B |
| CAMK2B | MDM2 |
| SPOP | PSMD10 |
| LONRF1 | HERC4 |
| CTNNB1 | PSMD7 |
| GAN | LONRF1 |
| RPL35A | NCBP2 |
| YBX1 | RBM22 |
| RBBP6 | MGRN1 |
| HERC4 | TRIM71 |
| FBXL16 | FBXO21 |
| RNF4 | HERC4 |
| FBXL19 | FBXO21 |
| ANAPC16 | PSMD7 |
| TRIP12 | MGRN1 |
| ATF2 | SMARCC1 |
| HERC4 | MGRN1 |
| TPX2 | YWHAE |
| SLC38A9 | PRKAA1 |
| RNF2 | ATF2 |
| DNASE1L1 | PDXK |
| FZD4 | SNAP91 |
| GUK1 | NT5E |
| BRPF1 | GATAD2A |
| LONRF1 | TRIM71 |
| ASAP1 | EXOC2 |
| ANAPC16 | PSMD10 |
| RBBP6 | ASB1 |
| SPOPL | PSMD7 |
| GAN | RBBP6 |
| FBXL16 | TULP4 |
| NUP160 | TAOK1 |
| UBE2A | RBBP6 |
| RBBP6 | TRIM21 |
| LONRF1 | FBXL19 |
| ARID1A | ATF2 |
| GAN | FBXL16 |
| PBRM1 | RUNX1 |
| TULP4 | FBXO21 |
| FBXO21 | MGRN1 |
| LDLR | CBL |
| TPD52 | CPD |
| RNF4 | KLHL42 |
| KLHL42 | FBXL19 |
| SPCS3 | RPL14 |
| NUP160 | MDM2 |
| GTF2A1 | ATF2 |
| GAN | RNF4 |
| HNRNPK | CBX2 |
| GTF2A1 | ARID1A |
| SPCS3 | RPL35A |
| MPRIP | ITGA2B |
| TRIM71 | RBBP6 |
| SPOPL | PSMD10 |
| DSN1 | TUBB |
| ANAPC16 | AURKA |
| PXN | TNC |
| NF1 | PSMD7 |
| FBXL16 | TRIM71 |
| RNF4 | FBXL16 |
| AKT2 | BRPF1 |
| FBXL16 | MGRN1 |
| FBXL16 | TRIM21 |
| HNRNPD | WBP11 |
| IGF2R | CBL |
| COPS7B | FBXO21 |
| LONRF1 | FBXO21 |
| GAN | MGRN1 |
| BCL2L11 | TLN1 |
| JUN | PHC2 |
| ALYREF | RBM22 |
| ANAPC16 | CDK2 |
| SPOP | SPOPL |
| HNRNPA2B1 | WBP11 |
| LONRF1 | KLHL42 |
| POU2F1 | NCBP2 |
| EGLN1 | PSMD10 |
| MGRN1 | MEGF8 |
| RNF4 | RBBP6 |
| LDLR | RAB5B |
| INTS8 | POU2F1 |
| GTF2A1 | JUN |
| GTF2A1 | NF1 |
| FBXL16 | RBBP6 |
| COPS7B | TULP4 |
| CENPQ | TAOK1 |
| PSAP | LPCAT1 |
| UBE2F | GAN |
| UBAP2L | CBX2 |
| KLHL42 | FBXO21 |
| HAUS5 | PAFAH1B1 |
| UBAP2L | RNF2 |
| DCAF16 | DCAF10 |
| RNF2 | ATN1 |
| GAN | TRIM71 |
| ATF2 | MSH2 |
| ATN1 | ATF2 |
| RNF4 | FBXO21 |
| BCL2L11 | SND1 |
| MGRN1 | TRIM21 |
| NCBP2 | MLLT1 |
| TPD52 | BLOC1S3 |
| TRIP12 | KLHL42 |
| KDM1A | PHC2 |
| LONRF1 | MGRN1 |
| LIMD1 | PSMD7 |
| LONRF1 | UBE2A |
| HERC4 | KLHL42 |
| UBE2F | RBBP6 |
| LONRF1 | RBBP6 |
| GAN | TULP4 |
| KLHL42 | ASB1 |
| KLHL42 | MGRN1 |
| PAFAH1B1 | CENPQ |
| GATAD2A | PHC2 |
| SND1 | ITGA2B |
| ATN1 | CBX2 |
| FZD4 | VAMP7 |
| UBE2F | MGRN1 |
| BLOC1S3 | IGF2R |
| RAB5B | IGF2R |
| FZD4 | WASL |
| CBX2 | ATF2 |
| CENPH | TAOK1 |
| DCAF16 | COPS7B |
| ATF2 | PHC2 |
| UBE2F | TRIM71 |
| RNF4 | ASB1 |
| EZH1 | UBAP2L |
| GRB2 | SGMS1 |
| KLHL42 | RBBP6 |
| SIN3A | ITGA2B |
| FBXO21 | TRIM21 |
| FZD4 | IGF2R |
| DSN1 | TAOK1 |
| FBXO21 | RBBP6 |
| UBE2F | TRIM21 |
| ANAPC16 | CBX2 |
| ANAPC16 | PHC2 |
| FBXL16 | KLHL42 |
| CNEP1R1 | WDR82 |
| FGFR2 | NCBP2 |
| GPR55 | CX3CL1 |
| KLHL42 | TRIM21 |
| KLHL42 | TULP4 |
| TRIM71 | FBXO21 |
| FBXO21 | ASB1 |
| SPCS3 | RPL38 |
| UBE2F | LONRF1 |
| GAN | ASB1 |
| MPRIP | SND1 |
| DCAF7 | COPS7B |
| HAUS5 | YWHAE |
| DCAF7 | DCAF16 |
| TRIM71 | MGRN1 |
| SIRPA | FRMPD3 |
| HAUS5 | TUBB4B |
| UBAP2L | CBX6 |
| STAT1 | CPSF6 |
| FBXL19 | COPS7B |
| DCAF7 | HIPK2 |
| TRIM71 | KLHL42 |
| BCL2L11 | MAGED1 |
| PCBP2 | RBM22 |
| HERC4 | FBXO21 |
| COPS7B | ACTB |
| SIRPA | RAB14 |
| PSAP | GPR55 |
| KMT2D | ITGA2B |
| SPCS3 | RPLP2 |
| AMER1 | PSMD10 |
| MPRIP | BCL2L11 |
| MYO9B | ROBO1 |
| ANAPC16 | RNF2 |
| DSN1 | TUBB4B |
| KLHL42 | COPS7B |
| CBX2 | RUNX1 |
| BLOC1S3 | CPD |
| CAPZA2 | APP |
| CBX6 | ATF2 |
| SDC2 | CBL |
| CBL | VAMP7 |
| AMER1 | PSMD7 |
| GAN | FBXO21 |
| HNRNPK | PHC2 |
| GRB2 | COPS7B |
| ASB1 | MGRN1 |
| FBXL16 | COPS7B |
| PAFAH1B1 | NUP160 |
| EGLN1 | PSMD7 |
| CSNK1A1 | EVC |
| UBE2F | TULP4 |
| DCAF7 | DCAF10 |
| PAFAH1B1 | CENPH |
| RUNX1 | TJP1 |
| GPR55 | APP |
| UBE2F | KLHL42 |
| UBE2F | FBXO21 |
| CBX6 | GATAD2A |
| RNF4 | MGRN1 |
| CBX6 | RUNX1 |
| CEP55 | TPX2 |
| ACTG1 | GAPDH |
| KMT2D | SRCAP |
| C1S | NDUFAF3 |
| ARID1A | TP53 |
| ATXN3 | CACNA1A |
| CRTC2 | PRKAA1 |
| HIST1H3D | H3F3C |
| ARID1A | SRCAP |
| TNKS2 | MCL1 |
| TJP1 | YBX3 |
| RPL14 | SERBP1 |
| PPP1R10 | WDR82 |
| JUN | STAT1 |
| XRN2 | XRN1 |
| SMC1A | MSH2 |
| GUF1 | MRPS9 |
| PHACTR2 | ACTC1 |
| PARP1 | TP53 |
| ACTB | PXN |
| MDC1 | SMARCC1 |
| ARF1 | RAB5B |
| CACNA1A | CALM1 |
| ACTG1 | TJP1 |
| TRIM71 | LIN28B |
| VEGFA | RCAN1 |
| TJP1 | NOTCH2 |
| ACTG1 | PXN |
| GJD3 | TJP1 |
| CLIC4 | CRKL |
| SOD2 | TP53 |
| H3F3B | CBX5 |
| SERBP1 | RPLP2 |
| AKT2 | CRTC2 |
| SEC63 | CANX |
| HK1 | C12orf5 |
| XPO1 | TP53 |
| HCFC1 | SRCAP |
| TBC1D9 | ELMOD2 |
| TSC22D3 | TSC22D2 |
| EIF3L | PSMD7 |
| DICER1 | DROSHA |
| HNRNPU | ACIN1 |
| HNRNPA2B1 | MBNL1 |
| GRB2 | INSRR |
| PBRM1 | ACTB |
| DDX39B | DHX9 |
| HIST1H3D | CBX5 |
| RPL23 | EEF1D |
| KMT2A | SETD5 |
| TRIP12 | SRCAP |
| SERBP1 | EEF2 |
| ASAP1 | ARF5 |
| CNOT1 | XRN1 |
| HIST1H3D | MLLT1 |
| PBRM1 | H3F3B |
| ACTG1 | PBRM1 |
| NCAPD2 | TPX2 |
| IRS4 | PTPRF |
| AGPAT4 | AGPAT5 |
| PBRM1 | HIST1H3D |
| MDC1 | TP53 |
| PPP1CC | PPP2R3A |
| ACTB | DYNLRB1 |
| GRB2 | WASL |
| NBR1 | CALCOCO2 |
| NCL | TP53 |
| MYO5A | CALM1 |
| MYBBP1A | RBM28 |
| KMT2A | KDM5A |
| GGT6 | GLUL |
| DUSP2 | TP53 |
| DCAF7 | DYRK3 |
| PBRM1 | H3F3C |
| SRRM2 | KMT2D |
| EIF4G1 | HNRNPD |
| CACNA1E | CACNA1A |
| DHX35 | RBM22 |
| LDLR | LCAT |
| HNRNPD | NAA50 |
| VEGFA | FGFR2 |
| ACTB | ITGA2B |
| DSN1 | CBX5 |
| MTF1 | SLC30A1 |
| LATS1 | AMOT |
| DLG5 | GUK1 |
| PARD3 | TJP1 |
| EEF1D | RPL38 |
| TFAM | TP53 |
| SON | RBBP6 |
| FANCM | REV1 |
| MTMR3 | PIK3C3 |
| ACTB | ARID1B |
| NUMA1 | EPB41 |
| DROSHA | XRN2 |
| ILF3 | HNRNPU |
| ACTG1 | ARID1A |
| SMC1A | POLE |
| AAR2 | RBM25 |
| YBX1 | TP53 |
| HIPK2 | MDM2 |
| EPB41 | CADM1 |
| ACTG1 | ARID1B |
| PBRM1 | ARID1B |
| CELF1 | CEBPD |
| GALNT3 | ST6GALNAC1 |
| DROSHA | DHX9 |
| HNRNPD | AURKA |
| PHF20L1 | BCL11A |
| FANCM | MSH2 |
| RPL23 | GUF1 |
| BCL2L11 | JUN |
| BUB3 | AURKA |
| ACTG1 | ITGA2B |
| KMT2A | MLLT1 |
| DICER1 | XRN2 |
| HNRNPK | MBNL1 |
| SON | SRSF7 |
| BCOR | RNF2 |
| DDX46 | RTN4 |
| ACTC1 | WASL |
| KDM1A | KDM5A |
| SP1 | MDM2 |
| MDM2 | CCND1 |
| EEF1D | RPLP2 |
| TMCO1 | ATP1A1 |
| EIF3L | NUMA1 |
| NCAPD2 | AURKA |
| ARID1A | HCFC1 |
| ATP1A1 | HADHB |
| CAMK2B | PIK3C3 |
| HMGA2 | LIN28B |
| GEN1 | FANCM |
| CPEB4 | AURKA |
| MBNL1 | DDX5 |
| CALM1 | PPP1CC |
| FBXL19 | H3F3C |
| FBXL19 | H3F3B |
| FBXL19 | HIST1H3D |
| LDLR | APP |
| RBBP6 | WDR82 |
| ETF1 | EEF2 |
| SLC39A10 | SLC30A1 |
| DDX39B | CPSF6 |
| CPEB2 | AURKA |
| CALM1 | ACTC1 |
| H3F3C | H3F3B |
| TMCO1 | SLC25A3 |
| SP1 | CCND1 |
| EIF3L | PRRC2B |
| PAFAH1B1 | LPCAT1 |
| CAMK2B | PPP1CC |
| LUM | COL8A1 |
| MME | CD9 |
| DHX9 | NCL |
| PHF20L1 | GATAD2A |
| HCFC1 | KMT2D |
| DROSHA | XRN1 |
| CCNL1 | CDK2 |
| NCAPD2 | RRM1 |
| DDX39B | HNRNPU |
| WEE1 | TP53 |
| DICER1 | TP53 |
| VEGFA | EGLN1 |
| TNPO2 | XPO1 |
| GRB2 | PXN |
| CRTC2 | ATF2 |
| EIF4G1 | XRN1 |
| LIN28B | IGF2BP1 |
| LUM | MDM2 |
| NBR1 | PIK3C3 |
| EIF4G1 | EEF2 |
| PLRG1 | PAFAH1B1 |
| TP53 | SOX4 |
| DICER1 | LIN28B |
| TNPO2 | HNRNPD |
| EEF2 | MRPS9 |
| ITGA5 | TNC |
| CPLX1 | VAMP7 |
| RPL14 | BTF3L4 |
| NFATC2IP | SRCAP |
| SOD2 | GAPDH |
| GEN1 | MSH2 |
| CTNNB1 | TP53 |
| EIF3L | EEF2 |
| PNISR | RBM25 |
| CELF1 | XRN1 |
| UBQLN1 | APP |
| TPP1 | CLN6 |
| KMT2A | PTOV1 |
| MCL1 | CDK2 |
| SND1 | RBM28 |
| ITGA5 | PXN |
| DICER1 | XRN1 |
| VEGFA | CCND1 |
| DROSHA | LIN28B |
| MDC1 | TPX2 |
| XPO1 | IPO7 |
| RTN4 | TMEM65 |
| KDM1A | KDM5C |
| DYNLRB1 | DNAH3 |
| NCL | MDM2 |
| MECP2 | YBX1 |
| ACTB | CAP2 |
| STAT1 | TP53 |
| TP53 | CDK6 |
| CACYBP | STIP1 |
| PPIA | GAPDH |
| EIF4G1 | EIF4EBP2 |
| VEGFA | MCL1 |
| VEGFA | TJP1 |
| ILF3 | NCL |
| SNRPB | DDX39A |
| BUB3 | RRM1 |
| NCAPD2 | SMC1A |
| KDM1A | SMARCC1 |
| WEE1 | CDK6 |
| PARD3 | AMOT |
| RBFOX2 | MBNL1 |
| ZBTB7A | TP53 |
| GAPDH | CCND1 |
| ALYREF | CPSF6 |
| SPEN | KMT2D |
| DDX3X | DHX9 |
| MCL1 | GAPDH |
| EEF2 | ATIC |
| MME | APP |
| CTNNB1 | GAPDH |
| ZFP36 | HNRNPD |
| HOXA9 | MLLT1 |
| DEK | PPP1CC |
| DDX39B | EEF2 |
| NCL | HNRNPU |
| GUK1 | TJP1 |
| FGFR2 | CRKL |
| DDX3X | SRSF9 |
| E2F8 | CDK2 |
| APP | LCAT |
| KAT6B | H3F3B |
| ATXN3 | USP13 |
| HNRNPU | CPSF6 |
| NFIB | NF1 |
| PARD3 | CTNNB1 |
| DCAF7 | HIPK1 |
| GTPBP8 | NAA50 |
| WNK1 | NAA50 |
| HNRNPK | MDM2 |
| IGF2R | VPS35 |
| UBQLN1 | STIP1 |
| AKT2 | GAPDH |
| H3F3C | CBX5 |
| HK1 | GAPDH |
| ATXN3 | TP53 |
| EEF2 | GAPDH |
| HIPK2 | MDM4 |
| HNRNPA2B1 | CPSF6 |
| VMA21 | ATP6V0A2 |
| TP53 | PRDM2 |
| PPP1CC | ACTC1 |
| MCL1 | CCND1 |
| BUB3 | PARP1 |
| KMT2D | KDM6B |
| FNIP1 | PRKAA1 |
| VEGFA | STAT1 |
| HOXA9 | MSI2 |
| DICER1 | HNRNPD |
| H3F3C | KAT6B |
| SGPL1 | SGMS1 |
| CSNK1A1 | CACYBP |
| DICER1 | SND1 |
| ETF1 | EIF4A1 |
| VEGFA | CDK6 |
| PBRM1 | KDM5C |
| SESN1 | TP53 |
| VEGFA | GAPDH |
| ALYREF | NCL |
| FGFR2 | MDM2 |
| ACTC1 | ACTN4 |
| EEF1D | EEF2 |
| RCAN1 | DYRK2 |
| SYNRG | SEC24D |
| ATXN3 | UBQLN1 |
| SEC63 | SEC24D |
| PPP1R10 | PPP2R3A |
| FZD4 | CTNNB1 |
| SNRPB | DHX35 |
| NANOS1 | TRIM2 |
| CYLD | TP53 |
| MTPN | CAPZA2 |
| DHX9 | EIF4A1 |
| XPO1 | DDX39B |
| NF1 | TP53 |
| EIF4EBP2 | EIF4A1 |
| VAPA | VAMP7 |
| MORF4L2 | PSMD10 |
| CTNNB1 | GLUL |
| KMT2D | WDR82 |
| DDX3X | EIF4A1 |
| RNF4 | HIPK2 |
| EIF4G1 | PCBP2 |
| ACTG1 | SMARCC1 |
| TP53 | DRAM1 |
| SOD2 | TFAM |
| BMPR2 | NIPA1 |
| FBXO42 | FBXO28 |
| ITGA5 | ACTN4 |
| DDX39B | MBNL1 |
| HNRNPK | TP53 |
| CITED2 | TP53 |
| TNPO2 | H3F3C |
| CEBPD | MBNL1 |
| TULP4 | RIMS2 |
| PARP1 | SP1 |
| VEGFA | SP1 |
| TNPO2 | H3F3B |
| JUN | GAPDH |
| PLRG1 | DCAF10 |
| NCBP2 | XRN1 |
| PRR14L | TMTC3 |
| GRB2 | APP |
| XRN2 | PCBP2 |
| BCL2L11 | TP53 |
| DICER1 | GAPDH |
| ILF3 | CPSF6 |
| MDH2 | GAPDH |
| WDR82 | PPP2R3A |
| RPS6KA2 | YBX1 |
| TUBB | GAPDH |
| DACH1 | TP53 |
| TNK2 | TP53 |
| NCL | HNRNPD |
| MBIP | ACTB |
| CNEP1R1 | PPP2R3A |
| DICER1 | CCND1 |
| WDR82 | MSI2 |
| STX16 | CPLX1 |
| INTS8 | ZNF609 |
| CNEP1R1 | PPP1R10 |
| PAFAH1B1 | RMND5A |
| MDC1 | ARID1B |
| APP | TP53 |
| VEGFA | HNRNPD |
| XPO1 | STAT1 |
| E2F8 | CDK6 |
| ACTB | ITGA5 |
| MECP2 | HIPK2 |
| NUCKS1 | DYRK3 |
| INSRR | SOS2 |
| SIN3A | PHF23 |
| CLIC4 | HIVEP2 |
| DDX3X | YBX1 |
| SLC44A1 | RNF165 |
| SOD2 | JUN |
| BCOR | ZBTB7A |
| JUN | APP |
| VEGFA | MDM2 |
| CTNNB1 | CDK6 |
| RBFOX2 | EPB41 |
| EIF4G1 | NCL |
| VPS13A | VPS35 |
| ATXN3 | PLEKHG4 |
| ZNF207 | SF3B3 |
| RPL23 | CTPS1 |
| NISCH | ITGA5 |
| EEF1D | RPL14 |
| KDM5A | PHF23 |
| MYBBP1A | TP53 |
| UBQLN1 | PSMD7 |
| VPS8 | VPS35 |
| XPO1 | PXN |
| CANX | GAPDH |
| MDC1 | CBX2 |
| SRRM2 | SRCAP |
| GLUL | GAPDH |
| ALYREF | EIF4A1 |
| KDM1A | KDM6B |
| CELF1 | NME8 |
| VEGFA | DICER1 |
| DSN1 | MDC1 |
| YBX1 | CCND1 |
| AKT2 | ATF2 |
| ACTB | TP53 |
| NCAPD2 | POLE |
| DLG1 | EPB41 |
| RBM25 | SRSF9 |
| XPO1 | DHX9 |
| TP53 | KDM6B |
| EZH1 | KDM6B |
| DHCR24 | TP53 |
| EEF2 | SF3B3 |
| KMT2A | SPEN |
| DROSHA | SND1 |
| KDM1A | SIN3A |
| PRRC2B | KMT2D |
| DACH1 | JUN |
| PLRG1 | RBM25 |
| PHACTR2 | ACTB |
| ALYREF | XRN1 |
| RBFOX2 | RBM22 |
| NF1 | SOS2 |
| FGFR2 | SOS2 |
| PARD3 | YWHAE |
| MDM2 | PSMD10 |
| GEN1 | RRM1 |
| ACTG1 | TUBB |
| RBBP6 | TP53 |
| PCBP2 | EIF4A1 |
| ACTG1 | ITGA5 |
| CTNNB1 | TRAF4 |
| CDK2 | GAPDH |
| DDX3X | XRN1 |
| HNRNPA2B1 | EEF2 |
| DROSHA | RBM28 |
| YBX1 | EIF4A1 |
| ANAPC16 | MSH2 |
| DSC3 | CTNNB1 |
| TNKS2 | PARP1 |
| DDX39B | MDC1 |
| EIF4A1 | XRN1 |
| CACYBP | DRG1 |
| DDX3X | TP53 |
| KMT2D | ANKRD11 |
| JUN | CDK2 |
| SEC16A | CANX |
| DNAH3 | NME8 |
| PPIA | ACTB |
| KDM5A | KDM6B |
| KDM6B | H3F3B |
| RRM1 | AURKA |
| SIN3A | MDC1 |
| KDM5A | RBBP6 |
| CCND1 | AURKA |
| SPEN | NOTCH2 |
| HEXIM1 | MDM2 |
| DDX39B | PCBP2 |
| PDE3B | NT5E |
| MDM2 | AURKA |
| E2F8 | CEP55 |
| KMT2A | HOXA9 |
| DACH1 | SIX4 |
| VPS35 | APP |
| NCL | UBTF |
| SRSF7 | CPSF6 |
| HIST1H3D | KDM6B |
| ENTPD4 | RRM1 |
| ACTB | TUBB |
| VEGFA | BCL2L11 |
| DHX35 | DDX39A |
| PCBP2 | EEF2 |
| CPEB2 | EEF2 |
| MYO9B | CALM1 |
| DICER1 | XPO1 |
| MDC1 | NCL |
| AHNAK | S100A11 |
| LONRF3 | RNF149 |
| BRF1 | TRAF4 |
| MTMR3 | PI4KB |
| PIP4K2B | MTMR3 |
| IKZF1 | ARID5B |
| CNKSR3 | NISCH |
| KDM5C | KDM6B |
| BDP1 | SMARCC1 |
| ARHGAP35 | PXN |
| EIF4G1 | VPS35 |
| HNRNPD | RRM1 |
| ACTB | CTNNB1 |
| ARL15 | SKIDA1 |
| ZNF778 | ANKRD11 |
| TET3 | SIN3A |
| ATXN3 | ATN1 |
| FBXL19 | RNF2 |
| PLEKHG4 | CACNA1A |
| TJP1 | ACTN4 |
| HNRNPK | KPNA6 |
| ENTPD4 | NT5E |
| ALYREF | XPO1 |
| PDLIM7 | LIMD1 |
| ACTG1 | CAP2 |
| BDP1 | BRWD1 |
| CPSF6 | SMARCC1 |
| ANP32A | DEK |
| PBRM1 | TP53 |
| AMER1 | TP53 |
| MME | CCND1 |
| VAPA | RTN4 |
| AKT2 | PPP2R3A |
| XPO1 | XRN1 |
| BRPF1 | AURKA |
| HMGA2 | ATN1 |
| RRM1 | CDK2 |
| HNRNPK | RPLP2 |
| RBFOX2 | HNRNPK |
| BTF3L4 | RPL38 |
| DSC3 | SDR42E1 |
| RPL35A | BTF3L4 |
| PXN | CBL |
| HIPK1 | MDM2 |
| GUK1 | PDE11A |
| TPX2 | CENPH |
| ITGA2B | ACTN4 |
| EIF4EBP2 | EEF2 |
| CELF1 | RBFOX2 |
| IPO7 | NUP160 |
| SIN3A | SMARCC1 |
| CCDC14 | FOPNL |
| PPP1CC | AURKA |
| PARP1 | NCL |
| TNC | ITGA2B |
| PIGP | ZMAT3 |
| ANKFY1 | PARP1 |
| POLE | FANCM |
| UROD | AASDH |
| DHX9 | EEF2 |
| TNPO2 | IPO7 |
| PARP1 | GAPDH |
| SPOP | TTLL12 |
| SECISBP2L | ETF1 |
| DEK | KAT6B |
| KDM1A | ZNF217 |
| HNRNPA2B1 | NCL |
| CELF1 | RRM1 |
| SLC37A4 | CRIM1 |
| IP6K2 | DUSP16 |
| HNRNPA2B1 | EIF4A1 |
| ZNFX1 | BRMS1L |
| STAT1 | GAPDH |
| ENTPD4 | PDE11A |
| CALM1 | GAPDH |
| RPL23 | BTF3L4 |
| DICER1 | CALCOCO2 |
| VPS35 | PIK3C3 |
| CANX | SLC25A3 |
| TM9SF2 | IGF2R |
| PDE11A | NT5E |
| EIF3L | EEF1D |
| RNF4 | MAZ |
| MDM2 | GAPDH |
| DROSHA | XPO1 |
| DDX5 | RBM28 |
| AGPAT5 | LPCAT1 |
| XPO1 | NCBP2 |
| JUN | PARP1 |
| SIN3A | IKZF1 |
| DHX9 | MBNL1 |
| SMOC1 | TES |
| DDX39B | BUB3 |
| GRB2 | ACTN4 |
| SND1 | ARF5 |
| CIRBP | HNRNPK |
| VEGFA | AKT2 |
| EIF4G1 | HNRNPK |
| SLC39A10 | MTF1 |
| VEGFA | NCL |
| POLE | REV1 |
| PRRC2A | SRCAP |
| PXN | CCND1 |
| KMT2A | HIPK1 |
| MYO10 | CALM1 |
| CTNNB1 | ACTN4 |
| ILF3 | HNRNPD |
| GUK1 | PDE3B |
| ZNF703 | CCND1 |
| EIF4A1 | SLC25A3 |
| MDC1 | FANCM |
| ENTPD4 | PDE3B |
| ASAP1 | PXN |
| CDK6 | MDM2 |
| SCRN1 | GUF1 |
| VEGFA | ELN |
| TMCO1 | SIX1 |
| KMT2D | TP53 |
| RPS6KA2 | JUN |
| RPL35A | RBM28 |
| IMPAD1 | CAPN7 |
| TJP1 | GAPDH |
| MDM4 | USP22 |
| CTNNB1 | ARID1A |
| DDX39B | DHX35 |
| BCL2L11 | CCND1 |
| ATP1A1 | TMF1 |
| ILF3 | PCBP2 |
| TJP1 | PPP2R3A |
| MPHOSPH9 | USP53 |
| RPL23 | MTRF1L |
| TFAM | TIMM17A |
| VEGFA | DEK |
| ZDHHC21 | PRR14L |
| SLC7A1 | SLC38A2 |
| EIF4EBP2 | SGPL1 |
| STIP1 | GAPDH |
| REV1 | MSH2 |
| DCAF7 | ZNF703 |
| DDX3X | TRIM21 |
| DDX39B | RPLP2 |
| DEK | SPOP |
| PRKAA1 | HADHB |
| FBXL19 | FBXO28 |
| DICER1 | SOX4 |
| CTNNB1 | CDK2 |
| HOXB9 | BTG1 |
| ETF1 | DRG1 |
| SIN3A | CBX5 |
| GUK1 | PDXK |
| CALM1 | YWHAE |
| SGK1 | TP53 |
| HNRNPA2B1 | CTNNB1 |
| SLC6A6 | SLC38A2 |
| FBXL19 | WDR82 |
| DSC3 | NCAPD2 |
| GUK1 | EPB41 |
| FZD4 | VANGL1 |
| HNRNPA2B1 | RBM25 |
| DDX39B | NAA50 |
| CD9 | GAPDH |
| CD9 | ITGA5 |
| DDX39B | ACIN1 |
| SCD | ACSL4 |
| CCNL1 | CCND1 |
| NUDT16L1 | PXN |
| HMGA2 | IGF2BP1 |
| SESN1 | PRKAA1 |
| E2F8 | CCND1 |
| XPO1 | KPNA6 |
| DROSHA | TRIM71 |
| FBXL19 | BCOR |
| PTOV1 | SPEN |
| MDC1 | ARID1A |
| MYO10 | TM4SF1 |
| HNRNPK | EEF2 |
| ACVR2B | RGMB |
| EEF2 | RBM22 |
| CRKL | ITGA5 |
| SLC6A6 | TP53 |
| EIF4G1 | YBX1 |
| FGFR2 | ARID1B |
| CYLD | CCND1 |
| SIN3A | SP1 |
| ALYREF | MBNL1 |
| BRPF1 | H3F3B |
| PPP1CC | ATF2 |
| ELN | ATP6V0A2 |
| WEE1 | CCND1 |
| HNRNPD | RBM25 |
| PCBP2 | STIP1 |
| STAT1 | TRIM21 |
| PPIA | CPSF6 |
| KDM1A | ESCO1 |
| PCBP2 | RPLP2 |
| CTNNB1 | PXN |
| CAMK2B | ATF2 |
| RPS6KA2 | PPP1CC |
| IMPAD1 | PRDM2 |
| UBE2A | WDR82 |
| PDLIM7 | TP53 |
| CALCOCO2 | TJP1 |
| UBAP2L | BCL9 |
| PXN | WASL |
| WDR82 | MSH2 |
| VEGFA | ZFP36 |
| ACTC1 | GAPDH |
| HMGA2 | IGF2BP2 |
| CD9 | F11R |
| KMT2A | KDM5C |
| MAPK8IP2 | APP |
| ABL2 | ASB1 |
| RBFOX2 | DDX5 |
| EEF2 | DDX39A |
| CAMK2B | GAPDH |
| PNISR | SRRM2 |
| CRKL | ITGA2B |
| BUB3 | DDX39A |
| GTF2A1 | CNOT1 |
| MECP2 | CBX5 |
| CTNNB1 | CBL |
| AHNAK | ACTN4 |
| SP1 | CDK2 |
| DHX9 | SND1 |
| RBBP6 | RBM25 |
| AGAP3 | TSC22D3 |
| CLIC4 | CLCC1 |
| DCAF7 | FBRS |
| MCL1 | PRKAA1 |
| MORF4L2 | KAT6B |
| POLE | GEN1 |
| NCBP2 | EIF4A1 |
| CD9 | NT5E |
| ACTG1 | RBFOX2 |
| KDM1A | HCFC1 |
| RBM25 | RBM22 |
| KDM1A | KMT2D |
| HNRNPU | DDX39A |
| CACYBP | CHORDC1 |
| VPS8 | RAB5B |
| JUN | MCL1 |
| NCL | EIF4A1 |
| XPO1 | CCND1 |
| JUN | CDK6 |
| EIF4A1 | GAPDH |
| LIN28B | CCND1 |
| STARD8 | PCTP |
| BCL2L11 | TRIM2 |
| KDM1A | CCND1 |
| TES | NME8 |
| SOD2 | MRPS9 |
| HK1 | MDH2 |
| NCL | EEF2 |
| BCL2L11 | TRAF1 |
| EMC1 | CANX |
| SOD2 | MDH2 |
| ACTB | EEF2 |
| SOD2 | DICER1 |
| SESN1 | SLC38A9 |
| PPP1R3E | CALM1 |
| COL25A1 | APP |
| SYNRG | IGF2R |
| HNRNPA2B1 | CNOT1 |
| VEGFA | ACTB |
| ARID1B | KMT2D |
| XPO1 | CNOT1 |
| ATIC | GAPDH |
| SLC6A6 | SLC39A10 |
| HIPK2 | CTNNB1 |
| DICER1 | JUN |
| YBX1 | NCL |
| MTRF1L | GUF1 |
| SIX1 | CCND1 |
| HNRNPA2B1 | RPLP2 |
| KPNA6 | STAT1 |
| STIP1 | EEF2 |
| FASTK | GRSF1 |
| VEGFA | NT5E |
| PEG10 | IGF2R |
| RPS6KA2 | PPP1R3E |
| TUBB | EEF2 |
| CAMK2B | PDE3B |
| PIP4K2B | ACTN4 |
| PCBP2 | XRN1 |
| TNPO2 | MSI2 |
| DDX3X | PPP1CC |
| TMF1 | CCDC186 |
| ZNF281 | SOX4 |
| PBRM1 | KMT2D |
| DICER1 | DDX5 |
| SNRPB | AAR2 |
| MDC1 | USP13 |
| SEC16A | GORASP2 |
| EIF4G1 | POLE |
| BCL2L11 | TSC22D3 |
| VPS8 | PIK3C3 |
| DACH1 | CCND1 |
| TP53 | REV1 |
| CADM1 | TP53 |
| DICER1 | ZNFX1 |
| H3F3C | KDM6B |
| SLC38A9 | SLC38A2 |
| UBAP2L | PRRC2A |
| MCL1 | MDM2 |
| ITGA5 | NT5E |
| CPEB2 | CPEB4 |
| SOD2 | SP1 |
| FGFR2 | CTNNB1 |
| ARF1 | PXN |
| BCL2L11 | GAPDH |
| DDX3X | CTNNB1 |
| PCBP2 | NCL |
| ARF1 | GORASP2 |
| GATAD2A | IKZF1 |
| ARHGAP35 | GRB2 |
| USP13 | USP22 |
| ACTB | CALM1 |
| CALM1 | DLG1 |
| VEGFA | EIF4G1 |
| DDX39B | NCL |
| LDLR | JUN |
| JUN | MDM2 |
| PRRC2A | HCFC1 |
| ABCB7 | SLC19A2 |
| UBE2A | PSMD10 |
| SRSF7 | MBNL1 |
| UBE2A | REV1 |
| CHORDC1 | BRMS1L |
| DROSHA | GAPDH |
| MCL1 | PARP1 |
| CDK6 | GAPDH |
| DDX3X | KDM5C |
| KDM5A | SON |
| VEGFA | CX3CL1 |
| DYNLRB1 | NME8 |
| RBM41 | SRSF9 |
| FGFR2 | TP53 |
| RCAN1 | APP |
| RPL38 | RBM28 |
| LATS1 | PARD3 |
| BBC3 | MDM2 |
| STK4 | TP53 |
| SGMS1 | CNIH1 |
| PPP1CC | WBP11 |
| SIN3A | ZBTB7A |
| LRP4 | APP |
| RNF2 | MDM2 |
| IVNS1ABP | BRMS1L |
| ACTG1 | CALM1 |
| KDM5C | SMC1A |
| MYO9B | PARD3 |
| PTOV1 | NT5E |
| POU2F1 | HCFC1 |
| XPO1 | CDK2 |
| TRIP12 | THRAP3 |
| PPIA | KAT6B |
| ETF1 | DDX39A |
| CPSF6 | DDX5 |
| MECP2 | SP1 |
| VEGFA | ATIC |
| GRB2 | AKT2 |
| CLIC4 | TP53 |
| STX16 | VPS35 |
| DICER1 | HNRNPK |
| CADM1 | MGRN1 |
| DHX9 | DDX39A |
| PRRC2A | BAG6 |
| MDC1 | MDM2 |
| FBXL19 | KMT2D |
| ELN | LUM |
| XPO1 | HNRNPK |
| MCL1 | ACTB |
| HNRNPK | TJP1 |
| UBQLN1 | UBE2A |
| VEGFA | CDK2 |
| SOD2 | DROSHA |
| MME | TP53 |
| PVR | NCR3LG1 |
| LDLR | SCD |
| EEF2 | TP53 |
| SNRPB | NCL |
| CTPS1 | RRM1 |
| ACTG1 | ACTC1 |
| TP53 | SMARCC1 |
| ILF3 | XPO1 |
| DDX3X | NCBP2 |
| DSG2 | NME8 |
| DDX39B | ETF1 |
| DHX9 | HEXIM1 |
| CLDN15 | TJP1 |
| ACTB | CCND1 |
| HIPK1 | RUNX1 |
| RPL35A | EEF1D |
| SCD | SLC25A3 |
| BAG6 | GAPDH |
| CNOT1 | IGF2BP1 |
| SERBP1 | SND1 |
| MME | NT5E |
| MYO5A | ACTB |
| AHNAK | CERCAM |
| MDC1 | CPSF6 |
| VEGFA | SLC16A1 |
| BUB3 | CDK2 |
| GAPDH | C12orf5 |
| CNEP1R1 | BRWD1 |
| DAAM2 | VANGL1 |
| GATAD2A | CBX5 |
| DLG5 | CTNNB1 |
| NR2C2 | ETF1 |
| DROSHA | SP1 |
| HNRNPA2B1 | DDX39A |
| AGPAT4 | LPCAT1 |
| XPO1 | XRN2 |
| CD9 | ITGA2B |
| VEGFA | EPHB4 |
| ACTG1 | EEF2 |
| ZNF217 | AURKA |
| POLE | TP53 |
| TMEM65 | TTC9 |
| GUK1 | CALM1 |
| SERBP1 | RBM25 |
| SMC1A | SF3B3 |
| SLC30A1 | NIPA1 |
| PIGP | CRYZL1 |
| TP53 | NOTCH2 |
| ARF1 | UBE2A |
| ARF1 | CANX |
| MYBBP1A | NCL |
| POLE | ARID1A |
| GAPDH | NME8 |
| DCAF10 | BUB3 |
| VPS13A | PANK1 |
| PARP1 | MSH2 |
| HNRNPA2B1 | NAA50 |
| SESN1 | DRAM1 |
| ROBO2 | EPHA4 |
| WNK1 | SLC12A7 |
| EIF4G1 | DDX5 |
| EZH1 | KDM1A |
| EGLN1 | PRKAA1 |
| FBXL19 | KDM6B |
| EIF3L | BTG1 |
| ZFP36 | JUN |
| LDLR | CTNNB1 |
| NBR1 | KIAA0226 |
| RTN4 | NACC2 |
| RAB5B | ARF5 |
| CLIC4 | YWHAE |
| WEE1 | RRM1 |
| PHC2 | DUSP3 |
| TRIP12 | CNOT1 |
| CENPH | AURKA |
| CRIM1 | TAOK1 |
| NBR1 | CEP55 |
| EEF1D | HNRNPK |
| SERBP1 | NCL |
| DCAF7 | EGLN1 |
| PARP1 | AURKA |
| IGF2BP2 | NOTCH2 |
| YWHAE | GAPDH |
| CELF1 | RBM20 |
| XPO1 | NCL |
| NF1 | MDM2 |
| SNRPB | DDX39B |
| PARP1 | EEF2 |
| CD9 | TM4SF1 |
| SATB1 | TP53 |
| CPSF6 | DDX39A |
| ZNF217 | BRMS1L |
| ACTG1 | MYO5A |
| CALCOCO2 | COL8A1 |
| AGPAT5 | PDE3B |
| PLEKHA1 | PLEKHA3 |
| ZFP36 | NCL |
| BAHCC1 | PHF23 |
| HNRNPD | EIF4A1 |
| SLIT2 | LUM |
| BCL2L11 | MDM2 |
| CALM1 | PIK3C3 |
| CTPS1 | ATIC |
| SPOP | FBXL19 |
| FBXL19 | SPOPL |
| MECP2 | TET3 |
| BRPF1 | H3F3C |
| SPCS3 | SEC63 |
| ZNF217 | CBX5 |
| GRB2 | TP53 |
| XRN2 | NCBP2 |
| DDX3X | DHX35 |
| HIPK2 | DDX39A |
| SGK1 | WNK1 |
| HIPK2 | DDX39B |
| ZNRF3 | CTNNB1 |
| BTF3L4 | CNOT1 |
| BRD2 | TP53 |
| ILF3 | YBX1 |
| ACTG1 | TP53 |
| ILF3 | SRSF7 |
| NBR1 | UBQLN1 |
| E2F8 | TPX2 |
| KDM5C | ARID5B |
| NF1 | APP |
| HNRNPD | IGF2BP1 |
| CTNNB1 | DSG2 |
| TP53 | SIX1 |
| HNRNPK | EIF4A1 |
| DSC3 | TP53 |
| LATS1 | CDK2 |
| EIF4G1 | SGK1 |
| IGF2R | TPP1 |
| EPHB4 | TP53 |
| GRB2 | JUN |
| CEP55 | NCAPD2 |
| PXN | GAPDH |
| DDX3X | HNRNPA2B1 |
| ACTC1 | CAP2 |
| SMC1A | USP13 |
| MYO5A | CAPZA2 |
| GRB2 | PRRC2A |
| ATXN3 | MBNL1 |
| MORF4L2 | SIN3A |
| KMT2A | FBXL19 |
| NCL | APP |
| GRB2 | PPP1CC |
| ATXN3 | PSMD7 |
| HES7 | NRARP |
| ACTG1 | PPP1CC |
| TNPO2 | SRSF9 |
| DDX3X | EEF2 |
| SRCAP | H3F3B |
| BRPF1 | HIVEP2 |
| CRKL | SOS2 |
| MASTL | ITGA2B |
| ACTG1 | PPIA |
| XPO1 | SF3B3 |
| NRARP | NOTCH2 |
| ZMAT3 | TP53 |
| HMGA2 | DICER1 |
| CRKL | TP53 |
| HNRNPA2B1 | IGF2BP1 |
| DCAF7 | PLRG1 |
| STAT1 | CCND1 |
| RBM20 | RBM25 |
| ARID1A | MSH2 |
| THRAP3 | HNRNPU |
| NCL | GAPDH |
| DICER1 | LIMD1 |
| NT5E | GAPDH |
| CTNNB1 | NOTCH2 |
| HNRNPD | XRN1 |
| CTPS1 | RBM28 |
| BUB3 | GEN1 |
| CALCOCO2 | CEP55 |
| SP1 | GAPDH |
| TFAM | GAPDH |
| RRM1 | TPX2 |
| ANKFY1 | RAB5B |
| PRKAA1 | ATIC |
| BRMS1L | CBX5 |
| BRD2 | SPOP |
| DDX46 | DHX35 |
| BAHCC1 | GATAD2A |
| ARL15 | BTG1 |
| SLC37A4 | TAOK1 |
| ZNF207 | MDC1 |
| TET3 | HCFC1 |
| UBTF | TP53 |
| ARF1 | WASL |
| KDM1A | CTNNB1 |
| CEP55 | TP53 |
| SPOP | TRAF1 |
| DYRK3 | ZNF609 |
| MCL1 | CDK6 |
| CTNNB1 | IGF2BP1 |
| BAG6 | USP13 |
| RPLP2 | NAA50 |
| RBM20 | RRM1 |
| SRSF7 | ACIN1 |
| SMC1A | CDK2 |
| SERBP1 | HNRNPA2B1 |
| ALYREF | EEF2 |
| SRSF7 | IGF2BP1 |
| BRD2 | HEXIM1 |
| GATAD2A | BRMS1L |
| SDR42E1 | TM7SF2 |
| VEGFA | CD151 |
| SESN1 | C12orf5 |
| POU2F1 | TP53 |
| AKT2 | EIF4EBP2 |
| YBX1 | RBM41 |
| ILF3 | IGF2BP1 |
| ACTG1 | TUBB4B |
| TP53 | TWSG1 |
| MYO9B | PPP1CC |
| PPIA | EEF2 |
| HNRNPK | NAA50 |
| CACNA1A | ATN1 |
| BMPR2 | IRS4 |
| ACTB | NT5E |
| KDM5C | CNOT1 |
| ATN1 | RERE |
| PRRC2A | SFXN5 |
| DDX46 | SEC63 |
| UBTF | MDM2 |
| SATB1 | HNRNPU |
| PLRG1 | DCAF16 |
| MBIP | MORF4L2 |
| RBM41 | SF3B3 |
| JUN | HOXA9 |
| TSC22D3 | JUN |
| SNRPB | RBM41 |
| XPO1 | GAPDH |
| RBM41 | SF3B2 |
| IGF2BP2 | VAPA |
| RNF4 | SP1 |
| DICER1 | EEF2 |
| MDC1 | PARP1 |
| ZCCHC24 | ANKRD33B |
| KMT2A | KDM6B |
| ESCO1 | REV1 |
| CAPZA2 | CAP2 |
| HAUS5 | KPNA6 |
| DHX9 | CPSF6 |
| XPO1 | DDX39A |
| PEG10 | ZNF451 |
| HEXIM1 | MLLT1 |
| CEP55 | TMTC3 |
| AAR2 | EEF2 |
| ROBO1 | EPHA4 |
| UBE2A | FANCM |
| ZNF12 | GPM6B |
| ELP2 | ANKRD52 |
| CD9 | CANX |
| UBE2A | USP22 |
| RBM20 | ANKRD52 |
| DYRK2 | SRSF7 |
| SHANK2 | MECP2 |
| ILF3 | DDX5 |
| ZFP36 | DICER1 |
| MDM2 | C12orf5 |
| DROSHA | HNRNPK |
| IRS4 | CRKL |
| RGMA | HIPK2 |
| RPL14 | RBM28 |
| CNOT1 | PXN |
| ARL9 | AASDH |
| SP1 | HADHB |
| CRKL | CCND1 |
| EZH1 | EPB41 |
| KMT2A | KDM1A |
| GATAD2A | RERE |
| SLC38A7 | COPS7B |
| ACTB | S100A11 |
| ZFP36 | HNRNPA2B1 |
| HOXA9 | SOX4 |
| TP53 | H3F3B |
| HMGA2 | DROSHA |
| RBFOX2 | CEBPD |
| CTNNB1 | MSH2 |
| LIN28B | AURKA |
| SCD | GAPDH |
| PPP1R10 | HNRNPK |
| PARP1 | CCND1 |
| CRKL | CTNNB1 |
| TUBB4B | GAPDH |
| PTOV1 | POLE |
| ABL2 | WASL |
| TJP1 | DSG2 |
| AKT2 | CCND1 |
| VAPA | EPHA4 |
| NBPF11 | BCL9 |
| EMD | VMA21 |
| SERBP1 | FBRS |
| SOD2 | NME8 |
| PI4KB | PLEKHA3 |
| CRYZL1 | AMER1 |
| VEGFA | MAZ |
| PARP1 | CTNNB1 |
| FECH | MAFG |
| MYBBP1A | UBTF |
| SIRPA | TRIM2 |
| NR2C2 | ZNF740 |
| VGLL3 | ENTPD4 |
| MDH2 | HADHB |
| RNF2 | MLLT1 |
| CYLD | JUN |
| YBX1 | STIP1 |
| ARF1 | ETF1 |
| ABL2 | ARHGAP35 |
| SATB1 | CTNNB1 |
| DIAPH2 | S100A11 |
| SRCAP | SMARCC1 |
| GAN | CBX6 |
| UBE2A | CDK2 |
| XPO1 | DEK |
| RBFOX2 | DMWD |
| CANX | MLEC |
| RAB14 | NISCH |
| SGK1 | GAPDH |
| NUMA1 | BRMS1L |
| EIF4G1 | XPO1 |
| NUMA1 | TNKS2 |
| CYLD | TRAF1 |
| DICER1 | IGF2BP1 |
| AGPAT4 | TMEM65 |
| HNRNPK | AURKA |
| HNRNPK | CDK2 |
| YWHAE | ATIC |
| MAGED1 | TUBB |
| IP6K2 | TP53 |
| HK1 | TP53 |
| LRP4 | CTNNB1 |
| RPL35A | UBQLN1 |
| MYO5A | WASL |
| HOXA9 | WDR82 |
| SOD2 | ACTB |
| PARD3 | GUK1 |
| PLRG1 | EEF2 |
| MDH2 | SLC25A3 |
| EIF4G1 | RRM1 |
| HEXIM1 | CCND1 |
| DCAF7 | RAD54L2 |
| SYNRG | RBM28 |
| MECP2 | DEK |
| DDX3X | PCBP2 |
| MECP2 | KDM1A |
| LIMD1 | CTDSPL |
| SHANK2 | MTF1 |
| PPIA | DDX46 |
| ROBO2 | CALM1 |
| ZNF217 | ZNF703 |
| PARP1 | ARF5 |
| HOXA9 | KMT2D |
| KMT2A | WDR82 |
| FBXO42 | TP53 |
| FGFR2 | CCND1 |
| TRIP12 | LENG8 |
| EZH1 | KDM5C |
| SOD2 | FECH |
| ARL15 | GXYLT2 |
| SF3B2 | EEF2 |
| RAB5B | VPS35 |
| SOD2 | GLO1 |
| FBXO28 | CDK2 |
| BCL2L11 | ZBTB7A |
| ELN | GAPDH |
| MPHOSPH9 | KLHL42 |
| TNIP3 | EZH1 |
| JUN | MAZ |
| PTOV1 | GAPDH |
| KMT2A | GAPDH |
| HNRNPK | CTNNB1 |
| TJP1 | PXN |
| SNRPB | CPSF6 |
| RRM1 | GAPDH |
| ACTG1 | KDM1A |
| MYADM | CPEB4 |
| ARF1 | CALM1 |
| PCBP2 | IGF2BP1 |
| GUK1 | EEF2 |
| MYO10 | DHX35 |
| USP53 | CRKL |
| ETF1 | NAA50 |
| NOTCH2 | CCND1 |
| PTDSS1 | ACSL4 |
| ILF3 | EIF4A1 |
| HOXA9 | CDK2 |
| MECP2 | KDM5C |
| KMT2A | CTNNB1 |
| GTPBP8 | GUF1 |
| DICER1 | RBFOX2 |
| MPHOSPH9 | ARL15 |
| SULF2 | TP53 |
| ACTG1 | PDLIM7 |
| CALM1 | EEF2 |
| AMER1 | ARID1A |
| CRYZL1 | BRWD1 |
| HNRNPK | IVNS1ABP |
| SEC63 | SPPL3 |
| VEGFA | SOD2 |
| PI4KB | VAPA |
| POLE | MDM2 |
| EPB41 | TJP1 |
| EEF2 | RBM25 |
| BAG6 | NCR3LG1 |
| ALYREF | ETF1 |
| DLG5 | ANKRD52 |
| KMT2A | BRPF1 |
| CTPS1 | GLUL |
| CALCOCO2 | PIK3C3 |
| DRAM1 | C12orf5 |
| STK4 | TAOK1 |
| CSNK1A1 | SEC16A |
| SDR42E1 | NCAPD2 |
| NF1 | MSH2 |
| HNRNPK | WASL |
| CELF1 | HNRNPK |
| TAF2 | SF3B2 |
| SP1 | ZBTB7A |
| YBX1 | GAPDH |
| IPO7 | TP53 |
| EIF3L | NCL |
| VEGFA | MECP2 |
| MCL1 | STAT1 |
| UBTF | CDK2 |
| CBL | GAPDH |
| CTNNB1 | DDX5 |
| PANK1 | TP53 |
| ACTB | CANX |
| DACH1 | CPEB2 |
| RTN4 | APP |
| HMGA2 | GAPDH |
| DHX9 | SON |
| SOD2 | GLUL |
| LDLR | GAPDH |
| NANOS1 | SLC12A7 |
| VEGFA | YBX1 |
| RPL23 | DRG1 |
| ILF3 | DDX3X |
| EEF2 | DDX5 |
| PDLIM7 | ACTN4 |
| HMGA2 | CCND1 |
| SGK1 | PIK3C3 |
| CRYZL1 | SON |
| NF1 | EPHA4 |
| SIN3A | MAZ |
| HK1 | ATIC |
| HMGA2 | CDK6 |
| SOD2 | HK1 |
| PLEKHG4 | SRSF9 |
| ACSL4 | HADHB |
| ZFP36 | DUSP2 |
| PEG10 | MECP2 |
| CLDN15 | F11R |
| XPO1 | HNRNPA2B1 |
| KDM6B | GAPDH |
| DCAF7 | SGPL1 |
| BCL2L11 | PARP1 |
| TPX2 | CDK2 |
| HEXIM1 | TP53 |
| PPP1CC | ARF5 |
| EEF1D | MTPN |
| EPHB4 | VAPA |
| JUN | PXN |
| DICER1 | MBNL1 |
| SHANK2 | GUK1 |
| CRKL | WASL |
| SETD5 | PRDM2 |
| EZH1 | FBXL19 |
| VEGFA | HIPK2 |
| TM9SF3 | GORASP2 |
| RBFOX2 | FGFR2 |
| PRRC2A | ELN |
| BCL2L11 | AURKA |
| MECP2 | GPM6B |
| MBNL1 | DMWD |
| HOXA9 | TP53 |
| DDX3X | HNRNPK |
| STAT1 | CX3CL1 |
| PPP1CC | YWHAE |
| SPCS3 | ZDHHC21 |
| NANOS1 | ARL9 |
| MAGED1 | TRAF4 |
| DICER1 | CBX5 |
| GAPDH | SLC25A3 |
| SIRPA | GRB2 |
| DHCR24 | SCD |
| KDM6B | SMARCC1 |
| VEGFA | ILF3 |
| DDX39B | SF3B3 |
| OEBT | VANGL1 |
| ACTG1 | CCND1 |
| ANKFY1 | VPS35 |
| ANKFY1 | ZCCHC14 |
| NOTCH2 | GAPDH |
| CRTC2 | MDM2 |
| DDX3X | KMT2D |
| TRIP12 | UBQLN1 |
| EIF4G1 | GAPDH |
| CAMK2B | LONRF3 |
| MYO5A | ACTC1 |
| HMGA2 | MDM2 |
| SP1 | SMARCC1 |
| BRF1 | ELP2 |
| SP1 | NCL |
| VPS13A | ACTB |
| HIST1H3D | AURKA |
| CDK6 | AURKA |
| IKZF1 | RMND5A |
| KDM5C | ARID1A |
| TP53 | HOXA13 |
| XPO1 | EIF4A1 |
| ANP32A | DDX39B |
| SETD5 | UBTF |
| BRD2 | SFXN5 |
| KCTD20 | SLC38A2 |
| ARRDC1 | VAPA |
| HK1 | GLUL |
| GRB2 | GAPDH |
| SULF2 | NDST3 |
| TJP1 | WASL |
| ASAP1 | GRB2 |
| VPS8 | STX16 |
| CNOT1 | GAPDH |
| HNRNPD | EEF2 |
| SND1 | RBM25 |
| PSAP | IGF2R |
| HK1 | ACTB |
| DDX46 | CNOT1 |
| ELP2 | KCTD20 |
| RPL23 | TP53 |
| NF1 | CCND1 |
| ESCO1 | CDK2 |
| HNRNPA2B1 | GAPDH |
| BCOR | TP53 |
| RPL35A | DDX39B |
| EZH1 | KDM5A |
| CCDC14 | GEN1 |
| IGF2BP2 | IGF2BP1 |
| EEF2 | RRM1 |
| LDLR | HNRNPD |
| MECP2 | GATAD2A |
| KIAA0226 | EXOC2 |
| GUK1 | VANGL1 |
| LIN28B | CDK6 |
| SERP1 | CANX |
| FBXL16 | CPLX1 |
| UBQLN1 | PSMD10 |
| DNASE1L1 | ETNK1 |
| CALM1 | ARF5 |
| F11R | DSG2 |
| EEF2 | EXOC2 |
| PARP1 | CDK2 |
| RPL23 | UBQLN1 |
| ALYREF | TRIP12 |
| RAB5B | VAPA |
| STAT1 | PCBP2 |
| ROBO1 | CALM1 |
| E2F8 | MSH2 |
| YBX1 | SERBP1 |
| JUN | MAFG |
| SEC63 | CTDSPL |
| SLC16A1 | HK1 |
| GLO1 | ATIC |
| UBQLN1 | RPL38 |
| XPO1 | EEF2 |
| DDX3X | BCOR |
| CSNK1A1 | MDM4 |
| RPL14 | UBQLN1 |
| CTPS1 | GUF1 |
| SLC7A1 | HADHB |
| TFAM | GRSF1 |
| CTPS1 | DRG1 |
| BUB3 | WDR82 |
| XPO1 | MDM2 |
| ACTG1 | CTNNB1 |
| KMT2A | HIST1H3D |
| ARF1 | PARP1 |
| CAMK2B | SON |
| SESN1 | MDM2 |
| RPLP2 | HNRNPU |
| CBX6 | MLLT1 |
| GEN1 | REV1 |
| RBM25 | MSH2 |
| PPP1CC | NCL |
| ALYREF | EIF4G1 |
| ACSL4 | AGPAT5 |
| DDX39B | HNRNPD |
| EZH1 | DCAF10 |
| SCRN1 | WASL |
| DRG1 | TLN1 |
| DROSHA | HNRNPA2B1 |
| HIST1H3D | KMT2D |
| E2F8 | WEE1 |
| RPS6KA2 | CCND1 |
| RPLP2 | GAPDH |
| NF1 | CDK6 |
| TMEM64 | HIPK1 |
| NF1 | KMT2D |
| BCL2L11 | CDK2 |
| DDX46 | SIN3A |
| GAPDH | AURKA |
| BBC3 | SESN1 |
| NF1 | AURKA |
| TRIM38 | TRIM2 |
| SESN1 | ZMAT3 |
| SIN3A | PBRM1 |
| RPL14 | NCL |
| VEGFA | ACTG1 |
| THRAP3 | ACIN1 |
| UBQLN1 | BAG6 |
| ARID1A | ARID5B |
| KMT2D | H3F3B |
| TJP1 | CCND1 |
| AAR2 | RBM22 |
| TIMM17A | SLC25A3 |
| CITED2 | CECR2 |
| MYO10 | TP53 |
| MDM4 | NF1 |
| DACH1 | YBX1 |
| USP53 | SEC24D |
| ATXN3 | MGRN1 |
| DICER1 | BCL2L11 |
| NR2C2 | THRAP3 |
| HNRNPD | DDX39A |
| EIF4EBP2 | SGK1 |
| RAB14 | PIK3C3 |
| SON | PPP1CC |
| UBQLN1 | ETF1 |
| SHANK2 | MYO5A |
| CALM1 | IP6K2 |
| DYRK2 | CPSF6 |
| STAT1 | EIF1AD |
| DICER1 | CDK6 |
| PBRM1 | SRCAP |
| IRS4 | INSRR |
| USP13 | PIK3C3 |
| ARF1 | RAB14 |
| SAMD1 | BRPF1 |
| XRN2 | RBM28 |
| IKZF1 | TP53 |
| MASTL | EEF2 |
| CNOT1 | DDX5 |
| PCBP2 | YBX3 |
| STAT1 | CDK2 |
| CEBPD | RRM1 |
| ILF3 | MBNL1 |
| BCOR | KMT2D |
| MSI2 | MDM2 |
| KDM5C | RNF2 |
| KMT2D | PRDM2 |
| SMARCC1 | MSH2 |
| SERBP1 | SRRM2 |
| ZNF207 | ZBTB47 |
| PPIA | UBE2A |
| ATN1 | GAPDH |
| GUK1 | YBX3 |
| TMF1 | WDR82 |
| DCAF10 | TTC9 |
| KMT2A | TP53 |
| UBAP2L | CPSF6 |
| LPGAT1 | AGPAT4 |
| CTNNB1 | MDM2 |
| ALYREF | DEK |
| PIK3C3 | GAPDH |
| ANP32A | STAT1 |
| JUN | DUSP16 |
| UBE2A | PSMD7 |
| CELF1 | DSG2 |
| PRKAA1 | AASDH |
| XRN2 | DDX5 |
| CDK2 | MSH2 |
| DEK | MLLT1 |
| CAMK2B | CACNA1A |
| PTOV1 | JUN |
| FBXL19 | KDM5C |
| DCAF10 | SLC25A51 |
| TP53 | ATIC |
| HMGA2 | NFIB |
| GUF1 | AASDH |
| SPOP | TP53 |
| ARID1B | SRCAP |
| RBM25 | DDX39A |
| EMD | ACTB |
| LDLR | DHCR24 |
| EIF1AD | GAPDH |
| SIN3A | ARID1A |
| PARP1 | ATIC |
| RBFOX2 | MSI2 |
| COPS7B | PSMD7 |
| UBAP2L | YBX1 |
| NAT8L | NAA50 |
| DICER1 | ZMAT3 |
| PAFAH1B1 | MGRN1 |
| ZBTB41 | BRWD1 |
| HNRNPD | GAPDH |
| JUN | CX3CL1 |
| MTERF4 | GRSF1 |
| AKT2 | JUN |
| FGFR2 | ARID1A |
| SLC38A7 | CNOT1 |
| WEE1 | MDM2 |
| DACH1 | SIN3A |
| CSNK1A1 | MDM2 |
| BCOR | YWHAE |
| DDX46 | AAR2 |
| GUK1 | SRSF9 |
| GRSF1 | CNIH1 |
| SERBP1 | SRSF7 |
| HK1 | BAG6 |
| EMD | ST8SIA2 |
| DDX46 | RBM28 |
| MDC1 | RNF2 |
| TM4SF1 | ITGA5 |
| CIRBP | RBM25 |
| IGF2BP2 | LIN28B |
| ILF3 | SON |
| DHX35 | DDX5 |
| XPO1 | VPS35 |
| GRB2 | ITGA2B |
| RNF2 | DDX5 |
| PAFAH1B1 | DYNLRB1 |
| CACNA1A | RIMS2 |
| TMCO1 | UBTF |
| CECR2 | KAT6B |
| LDLR | CANX |
| PIK3C3 | EXOC2 |
| HNRNPD | MSH2 |
| ACTB | CDK2 |
| TNK2 | AMOT |
| DEK | SERBP1 |
| DDX3X | CNOT1 |
| CEBPD | JUN |
| RRM1 | TP53 |
| CEBPD | SOX4 |
| ZNF609 | ANKRD52 |
| KDM5A | SIN3A |
| CALCOCO2 | F11R |
| RBM20 | DSG2 |
| LATS1 | EZH1 |
| ZFP36 | GAPDH |
| SHANK2 | DLG1 |
| MDC1 | MSH2 |
| MDC1 | GEN1 |
| SLIT2 | EPHA4 |
| SNRPB | SPOP |
| LATS1 | MDM2 |
| DHCR24 | EVC |
| HOXA9 | PHF23 |
| STAT1 | NCL |
| CELF1 | NCL |
| ANAPC16 | DCAF10 |
| PCBP2 | ZHX3 |
| STK4 | LIMD1 |
| BRPF1 | CECR2 |
| KDM1A | GAPDH |
| SCD | ADIPOR1 |
| CTNNB1 | BRMS1L |
| TFAM | ATIC |
| ACTB | EPB41 |
| CTNNB1 | SP1 |
| GLUL | SLC38A2 |
| SEC63 | SEC16A |
| MDM2 | SOX4 |
| TSC22D3 | CCND1 |
| TRIP12 | GAPDH |
| SLC6A6 | JUN |
| VEGFA | HEXIM1 |
| DRG1 | EEF2 |
| PPP1CC | RBBP6 |
| SULF2 | CCND1 |
| UBQLN1 | SRSF7 |
| HNRNPD | CCND1 |
| MDM2 | PRDM2 |
| GLO1 | STIP1 |
| SCD | LPCAT1 |
| CDK2 | BRMS1L |
| CLIC4 | ACTB |
| SPCS3 | PHACTR2 |
| TM4SF1 | CCND1 |
| EIF4G1 | DHX9 |
| MTERF4 | TFAM |
| TNKS2 | CTNNB1 |
| HK1 | XPO1 |
| FGFR2 | ITGA5 |
| TMF1 | GORASP2 |
| KMT2D | NOTCH2 |
| RPL23 | ILF3 |
| ATP1A1 | CLCC1 |
| E2F8 | AURKA |
| ACTB | APP |
| GRB2 | ERRFI1 |
| MDC1 | REV1 |
| CRKL | ABI2 |
| ACTB | IGF2BP1 |
| EIF4EBP2 | HNRNPA2B1 |
| DHX9 | ACTB |
| MYADM | FRMPD3 |
| BRD2 | SRCAP |
| DHX9 | AURKA |
| STAT1 | ACTB |
| TIMM17A | PSMD7 |
| DDX3X | SP1 |
| KMT2A | PBRM1 |
| BCOR | SP1 |
| EEF2 | SRRM2 |
| VEGFA | PSMD10 |
| AMD1 | ATIC |
| ACSL4 | ABCD2 |
| SPEN | RERE |
| PDLIM7 | MDM2 |
| SF3B3 | DHX35 |
| H3F3B | AURKA |
| EEF2 | PPIC |
| RCAN1 | INHBA |
| HES7 | CTNNB1 |
| UROD | ATIC |
| SATB1 | IKZF1 |
| SRCAP | MLLT1 |
| GRB2 | CCND1 |
| HES7 | NOTCH2 |
| ATP6V0A2 | APP |
| TM7SF2 | PTGIS |
| BRF1 | TP53 |
| ACTB | PPP1CC |
| DCAF10 | RRM1 |
| RRM1 | MDM2 |
| HIST1H3D | KAT6B |
| GRB2 | STAT1 |
| CELF1 | ILF3 |
| AMOT | DIAPH2 |
| NCBP2 | NCL |
| SIN3A | KDM5C |
| CITED2 | ROBO2 |
| ZMAT3 | MDM2 |
| BMPR2 | INHBA |
| MDH2 | ST8SIA2 |
| EMD | CTNNB1 |
| STX16 | ST8SIA2 |
| ACTG1 | CAPZA2 |
| NR2C2 | TRIP12 |
| RUNX1 | KAT6B |
| HERC4 | KAT6B |
| EIF4G1 | CCND1 |
| ZBED6 | ZFP14 |
| ADAMTS5 | GAPDH |
| PI4KB | ARF5 |
| PPP1CC | CDK2 |
| PRKAA1 | PPP1CC |
| EIF4G1 | RBM25 |
| MDH2 | NME8 |
| PVR | TJP1 |
| HNRNPK | IGF2BP1 |
| GRB2 | YWHAE |
| TMF1 | TM9SF3 |
| XPO1 | TUBB |
| XPO1 | TUBB4B |
| AAR2 | NCBP2 |
| PIP4K2B | TLN1 |
| EIF4A1 | YBX3 |
| IRS4 | NISCH |
| CITED2 | PDLIM7 |
| XPO1 | SRSF7 |
| EIF4G1 | HNRNPA2B1 |
| MSH2 | CCND1 |
| TES | VANGL1 |
| POTEG | MTPN |
| CD151 | ITGA5 |
| GREB1 | STAT1 |
| ZFP14 | SLC35E2 |
| STIP1 | MSI2 |
| TM9SF2 | GORASP2 |
| CELF1 | HNRNPA2B1 |
| UBE2A | NFATC2IP |
| INTS8 | UBAP2L |
| SATB1 | ZNF217 |
| BMPR2 | TWSG1 |
| GRB2 | SPEN |
| MDM4 | CDK6 |
| CAPZA2 | C12orf5 |
| EIF4A1 | CCND1 |
| KDM5C | KMT2D |
| SIN3A | CECR2 |
| SAMD1 | PODNL1 |
| ILF3 | SRRM2 |
| BAG6 | ZNF507 |
| IPO7 | KPNA6 |
| PIP4K2B | PIK3C3 |
| SRSF7 | RBM25 |
| ILF3 | GLUL |
| RNF4 | PARP1 |
| DROSHA | CDK6 |
| XPO1 | CHORDC1 |
| TFAM | PARP1 |
| RBM20 | ACTC1 |
| DDX3X | DDX5 |
| NCBP2 | CAPN7 |
| REV1 | GAPDH |
| TRIP12 | USP21 |
| TM7SF2 | TMTC3 |
| PARD3B | TJP1 |
| TULP4 | ZNF609 |
| BUB3 | HNRNPA2B1 |
| MORF4L2 | TCEAL1 |
| TNK2 | STAT1 |
| XRN2 | UBE2A |
| REV1 | AASDH |
| NACC2 | CBX5 |
| CYLD | USP21 |
| BDP1 | NCL |
| VAPA | TJP1 |
| CACYBP | EXOC2 |
| KIAA0226 | DRAM1 |
| TP53 | PXN |
| PHC2 | SLC25A3 |
| BCL2L11 | CDK6 |
| IGF2R | FBRS |
| CDK2 | DSG2 |
| ILF3 | SERBP1 |
| GEN1 | SIX1 |
| SGPL1 | ADIPOR1 |
| NRARP | ZBTB7A |
| KDM5A | H3F3B |
| TRAF1 | TP53 |
| FZD4 | DLG1 |
| CDK6 | NOTCH2 |
| MBNL1 | CPSF6 |
| VEGFA | KDM5A |
| HNRNPK | BUB3 |
| DDX39B | XRN1 |
| BUB3 | HNRNPU |
| ACTG1 | JUN |
| MECP2 | MBD6 |
| TRAF1 | JUN |
| EMD | TM7SF2 |
| FBXO28 | RMND5A |
| EIF4A1 | RBM25 |
| HNRNPA2B1 | ZMAT3 |
| ETF1 | PCBP2 |
| ILF3 | DDX39A |
| CECR2 | SRCAP |
| STK4 | STK38 |
| SGMS1 | VAPA |
| HOXA9 | RUNX1 |
| VEGFA | YBX3 |
| STAT1 | USP13 |
| KDM5C | H3F3B |
| SIN3A | KDM6B |
| SPOPL | CPSF6 |
| STX16 | EXOC2 |
| NCL | WDR82 |
| SCD | SGMS1 |
| MYO10 | EPB41 |
| EIF4G1 | DDX39B |
| IGF2BP2 | HNRNPD |
| KMT2A | PRDM2 |
| CRISP1 | DUSP2 |
| XPO1 | ATF2 |
| PRDM2 | DUSP3 |
| PARD3B | TAOK1 |
| SIN3A | SRCAP |
| PTDSS1 | LPGAT1 |
| MANBAL | ZCCHC9 |
| ACTG1 | YWHAE |
| SIN3A | VAPA |
| ARF1 | PIK3C3 |
| ARF1 | PPP1CC |
| PIGP | BRWD1 |
| CBX2 | MLLT1 |
| MME | CTNNB1 |
| ARID1A | MDM2 |
| ZNF217 | CCND1 |
| ARID1B | TP53 |
| DROSHA | MRPS9 |
| PARP1 | MDM2 |
| VEGFA | NOTCH2 |
| RERE | RIMS2 |
| APP | CANX |
| DFFA | TP53 |
| ZNF740 | PCBP2 |
| ATXN3 | MDC1 |
| MME | EDN3 |
| VEGFA | HK1 |
| UBQLN1 | RPLP2 |
| POLE | CBX5 |
| NFIB | ELN |
| TRIP12 | FANCM |
| LATS1 | PPP1CC |
| DROSHA | CCND1 |
| HNRNPK | GAPDH |
| RBM25 | WBP11 |
| RTN4 | CANX |
| XRN2 | CNOT1 |
| THRAP3 | RBM25 |
| SATB1 | SIN3A |
| SP1 | HEXIM1 |
| RNF2 | IVNS1ABP |
| MECP2 | GAPDH |
| KDM5A | FBXL19 |
| SOD2 | BCL2L11 |
| LPGAT1 | PRKAA1 |
| KMT2A | CBX6 |
| STAT1 | DUSP3 |
| VEGFA | NF1 |
| EMC1 | TMEM65 |
| TFAM | SLC7A1 |
| HNRNPK | PPP1CC |
| SOD2 | AKT2 |
| ARF1 | GAPDH |
| CALM1 | CTNNB1 |
| ZNF217 | CBX2 |
| RPL35A | HNRNPK |
| SERBP1 | DDX39A |
| SYNRG | ARF1 |
| NCAPD2 | ESCO1 |
| GAPDH | AASDH |
| ARF1 | XPO1 |
| NCL | DDX5 |
| MCL1 | USP13 |
| CITED2 | INSRR |
| GUK1 | GAPDH |
| PLRG1 | BUB3 |
| GLO1 | GAPDH |
| IPO7 | SLC25A3 |
| PARD3 | AURKA |
| RPL35A | NUMA1 |
| RGMA | RTN4 |
| TRAF1 | NFATC2IP |
| CNKSR3 | ILF3 |
| SLC39A10 | SLC19A2 |
| PARD3B | YWHAE |
| BRD2 | KDM6B |
| RPL23 | ATIC |
| ACTG1 | CANX |
| BTF3L4 | RPLP2 |
| BRWD1 | ADCK2 |
| EPB41 | PXN |
| DEK | THRAP3 |
| POTEM | CAP2 |
| TET3 | CCND1 |
| DICER1 | ILF3 |
| PIK3C3 | DRAM1 |
| POTEG | CAP2 |
| VEGFA | LDLR |
| CELF1 | DDX5 |
| APP | CAPN7 |
| EEF2 | GUF1 |
| NF1 | CTNNB1 |
| MORF4L2 | KPNA6 |
| FNIP1 | SLC38A9 |
| HCFC1 | EEF2 |
| SRRM2 | WDR82 |
| ITGA2B | GAPDH |
| SF3B2 | DHX35 |
| PRKAA1 | YWHAE |
| JUN | NF1 |
| TP53 | CAPN7 |
| THRAP3 | SF3B2 |
| ADIPOR1 | ATIC |
| EEF1D | YBX1 |
| PRKAA1 | TLN1 |
| ATP1A1 | ITGA5 |
| CIRBP | EEF2 |
| KMT2A | PIK3C3 |
| ALYREF | THRAP3 |
| MDH2 | GLUL |
| RBM25 | DDX5 |
| ATP1A1 | PSAP |
| BRD2 | BRPF1 |
| EIF4G1 | ILF3 |
| CEP55 | CENPH |
| SFXN5 | WBP11 |
| KAT6B | ESCO1 |
| ROBO1 | HOXB2 |
| MYBBP1A | IPO7 |
| HNRNPU | MBNL1 |
| EEF1D | EIF4A1 |
| SNRPB | HCFC1 |
| EEF2 | AURKA |
| BCL11A | SMARCC1 |
| FGFR2 | GAPDH |
| PREX1 | C21orf58 |
| CPSF6 | WBP11 |
| PHLDB2 | KIF21A |
| SPCS3 | EMC1 |
| XPO1 | SLC30A1 |
| ARF1 | SLC38A9 |
| MECP2 | HCFC1 |
| ASAP1 | CBL |
| SMC1A | SMARCC1 |
| ITGA5 | YWHAE |
| ARHGAP35 | WASL |
| THRAP3 | DDX5 |
| JUN | DHX9 |
| DLG5 | PARD3B |
| MSI2 | TP53 |
| KCTD20 | ZCCHC14 |
| SRSF7 | MSH2 |
| SETD5 | ARID1B |
| TCEAL1 | TP53 |
| SNRPB | EEF2 |
| ACIN1 | DDX39A |
| FBXL19 | PPP1R10 |
| IPO7 | UBTF |
| JUN | DUSP2 |
| STX16 | ATP6V0A2 |
| BMPR2 | GAPDH |
| PAFAH1B1 | FOPNL |
| PRKAA1 | ZCCHC9 |
| EIF4A1 | DHX35 |
| MME | ACTB |
| CNOT1 | DDX39A |
| NR2C2 | PDLIM7 |
| NF1 | H3F3B |
| DHCR24 | RTN4 |
| KAT6B | MLLT1 |
| SCD | LCAT |
| MDC1 | HNRNPU |
| ATN1 | ZNF609 |
| AMOT | MBNL1 |
| SIN3A | NOTCH2 |
| CSNK1A1 | CPSF6 |
| FJX1 | VANGL1 |
| XPO1 | USP21 |
| GATAD2A | SMARCC1 |
| CALM1 | NME8 |
| BDP1 | VPS35 |
| MECP2 | SMC1A |
| ACSL4 | CANX |
| DHX9 | CTNNB1 |
| LMBR1L | LUM |
| ABI2 | SOS2 |
| UBE2A | CBX5 |
| ARF1 | ABI2 |
| PCBP2 | NAA50 |
| FANCM | SRCAP |
| UBTF | TRIM21 |
| AHNAK | SEC16A |
| YBX1 | BUB3 |
| STK4 | AMOT |
| CITED2 | SLIT2 |
| PANK1 | ACTC1 |
| ACTN4 | WASL |
| SOX4 | CCND1 |
| CYLD | TRIM38 |
| TES | SP1 |
| DDX46 | EEF1D |
| BRD2 | SMARCC1 |
| BBC3 | DRAM1 |
| UEVLD | UBQLN1 |
| CPSF6 | RBM25 |
| HNRNPK | DDX39A |
| DICER1 | ACTB |
| UBTF | CCND1 |
| DHX9 | RBM25 |
| SOD2 | APP |
| GLO1 | CALM1 |
| GUF1 | UROD |
| LDLR | SP1 |
| EEF2 | ACTC1 |
| DICER1 | HIST1H3D |
| RRM1 | NME8 |
| MDM4 | YWHAE |
| HIST1H3D | PPP1R10 |
| SMC1A | FANCM |
| DSN1 | AURKA |
| SOD2 | HEPH |
| NFIB | CADM1 |
| PARP1 | CAPN7 |
| MANBAL | CERCAM |
| BBC3 | ZMAT3 |
| MCL1 | PIK3C3 |
| FBXL19 | SRCAP |
| TP53 | PIK3C3 |
| SOD2 | VAPA |
| EEF1D | AHNAK |
| VAPA | CPLX1 |
| ATP6V0A2 | SLC25A3 |
| ILF3 | IVNS1ABP |
| YBX1 | EEF2 |
| PCBP2 | RBM25 |
| ACIN1 | RBM25 |
| MBIP | PPIC |
| CAPZA2 | ACTB |
| TNK2 | KIAA0226 |
| ZNFX1 | ZNF507 |
| IGF2R | TP53 |
| ASAP1 | WASL |
| VKORC1L1 | CACYBP |
| ZNF217 | ARID1A |
| AMOT | TJP1 |
| KDM5A | HIST1H3D |
| AAR2 | SEC16A |
| CELF1 | SRSF9 |
| VEGFA | CEP55 |
| CSNK1A1 | NR1D2 |
| HIST1H3D | TP53 |
| AKT2 | SGK1 |
| NFIB | APP |
| XPO1 | MSI2 |
| CD151 | THRAP3 |
| DEK | TP53 |
| POU2F1 | GAPDH |
| LIN28B | TP53 |
| NCBP2 | DHX35 |
| FGFR2 | EPHA4 |
| RNF4 | MECP2 |
| DDX39B | PRRC2A |
| MDM2 | CANX |
| EIF4G1 | INSRR |
| E2F8 | MDM2 |
| RNF4 | HIPK1 |
| DDX39B | EIF4A1 |
| PPP1CC | MSI2 |
| SOD2 | GUF1 |
| NFIB | ZDHHC21 |
| MYBBP1A | SND1 |
| DICER1 | KDM6B |
| CEBPD | BCL2L11 |
| SERBP1 | PPP1CC |
| SRCAP | RBM25 |
| XRN2 | GATAD2A |
| MANEA | TTC9 |
| DCAF7 | MDH2 |
| TSC22D3 | CX3CL1 |
| VEGFA | BMPR2 |
| VEGFA | ITGA2B |
| DICER1 | FBXL19 |
| GAN | CBX5 |
| SMC1A | ACIN1 |
| SNRPB | EIF4A1 |
| FGFR2 | NOTCH2 |
| CIRBP | PLRG1 |
| VANGL1 | DLG1 |
| CELF1 | PCBP2 |
| CALM1 | STK38 |
| POLE | ESCO1 |
| DDX3X | CALM1 |
| DROSHA | ILF3 |
| ABL2 | GRB2 |
| DDX3X | DDX39B |
| CDK15 | AURKA |
| PHACTR2 | ZFP14 |
| MASTL | TCEAL1 |
| ATP1A1 | KCTD20 |
| KDM6B | AASDH |
| DICER1 | MECP2 |
| GTF2A1 | BRF1 |
| BCOR | ARID1A |
| CECR2 | H3F3B |
| PAFAH1B1 | GRB2 |
| MASTL | PPP2R3A |
| SOD2 | ATIC |
| CALM1 | TUBB |
| RPL14 | SCD |
| MYO9B | PREX1 |
| RNF2 | USP22 |
| SOD2 | SGK1 |
| EEF1D | HCFC1 |
| KDM5A | USP22 |
| PPIA | TP53 |
| MDM4 | CCND1 |
| GLUL | NME8 |
| ATP1A1 | SLC25A3 |
| CTNNB1 | DDX39A |
| TMX2 | CANX |
| NCL | XRN1 |
| CECR2 | ABCD2 |
| SP1 | RUNX1 |
| RPL35A | PCBP2 |
| CALM1 | DDX5 |
| ZNF217 | TP53 |
| SOD2 | PARP1 |
| PARP1 | NCAPD2 |
| DSC3 | TJP1 |
| KDM6B | UROD |
| BRPF1 | KDM5C |
| KLHL42 | STK38 |
| SGK1 | PRKAA1 |
| SHANK2 | TJP1 |
| SDR39U1 | FECH |
| LDLR | BDP1 |
| PCBP2 | ARF5 |
| CYLD | USP13 |
| SP1 | HCFC1 |
| DROSHA | BCL2L11 |
| SPEN | TP53 |
| DDX46 | VAPA |
| BRD2 | SIN3A |
| DDX39B | CNOT1 |
| PTPRF | NAA50 |
| ACTB | PIK3C3 |
| XPO1 | HNRNPU |
| RCAN1 | USP22 |
| AHNAK | EEF2 |
| IKZF1 | RUNX1 |
| SPOPL | ANKRD52 |
| ZNFX1 | TRIM21 |
| PRR14L | SRCAP |
| MME | ITGA2B |
| MECP2 | NF1 |
| EEF2 | YWHAE |
| ABL2 | ROBO2 |
| SNAP91 | CPLX1 |
| NBR1 | CALM1 |
| MECP2 | TP53 |
| TM9SF2 | SLC25A3 |
| DDX3X | HNRNPU |
| SYNRG | SNAP91 |
| DCAF10 | KMT2D |
| DHX9 | KAT6B |
| ARID5B | NACC2 |
| HMGA2 | TRIM71 |
| GEN1 | HNRNPD |
| HCFC1 | PSMD7 |
| KDM1A | SRCAP |
| STAT1 | NF1 |
| DAAM2 | ACTB |
| VEGFA | HMGA2 |
| MME | PDLIM7 |
| AMD1 | ATP6V0A2 |
| TNPO2 | HIST1H3D |
| XPO1 | DDX5 |
| CTNNB1 | POLE |
| GLO1 | GLUL |
| ARF1 | SEC16A |
| SPCS3 | CANX |
| AHNAK | HNRNPD |
| NUMA1 | UBTF |
| DDX3X | SND1 |
| BAHCC1 | ZNF507 |
| RPLP2 | PSMD7 |
| KMT2A | NCL |
| RTN4 | TJP1 |
| CTPS1 | MRPS9 |
| HNRNPA2B1 | RRM1 |
| ABL2 | PPP2R3A |
| DEK | SMC1A |
| HIST1H3D | KDM5C |
| CTNNB1 | AURKA |
| EMD | SEC16A |
| ALYREF | RPLP2 |
| ACTG1 | HK1 |
| ORMDL3 | SGPL1 |
| ZBED6 | SLC35E2 |
| CBX6 | FECH |
| DHCR24 | APP |
| F11R | GUK1 |
| TNC | SOX4 |
| KDM5C | HCFC1 |
| JUN | RUNX1 |
| GRB2 | ACTB |
| PVR | F11R |
| HK1 | IGF2R |
| MCL1 | MDM4 |
| EEF1D | ETF1 |
| PHF23 | KDM6B |
| BCL2L11 | DYNLRB1 |
| SPOPL | TTLL12 |
| RRM1 | GUF1 |
| CELF1 | RBM25 |
| IGF2BP2 | BCL11A |
| ZNF609 | RIMS2 |
| MBNL1 | ZSWIM6 |
| ANKFY1 | NISCH |
| SCD | ACTB |
| ZFP36 | ILF3 |
| CEBPD | RUNX1 |
| FBXL19 | PHF23 |
| DEK | TRIP12 |
| SETD5 | TRIP12 |
| IPO7 | NCL |
| EEF2 | CANX |
| GREB1 | CCND1 |
| ZFP36 | TSC22D3 |
| PIP4K2B | SPOP |
| SPCS3 | EXOC2 |
| RNF4 | RNF2 |
| DEK | MDM4 |
| SMC1A | WDR82 |
| BRPF1 | FBXL19 |
| ROBO2 | EPHB4 |
| TTLL9 | TTLL12 |
| DICER1 | CNOT1 |
| VEGFA | CD9 |
| EPB41 | APP |
| LATS1 | GAPDH |
| KDM1A | ACTB |
| PHF20L1 | AMD1 |
| CAPZA2 | ACTC1 |
| MASTL | CEP55 |
| KMT2A | NT5E |
| BDP1 | UBTF |
| SAMD1 | CECR2 |
| TFAM | HK1 |
| MYO5A | TRIM2 |
| ZBTB7A | SRCAP |
| ILF3 | NUCKS1 |
| POTEM | WASL |
| RPL35A | HNRNPA2B1 |
| VAPA | PLEKHA3 |
| SND1 | EEF2 |
| DEK | BUB3 |
| POTEG | WASL |
| RTN4 | ATIC |
| SCD | CAP2 |
| AHNAK | GORASP2 |
| DICER1 | MRPS9 |
| PAFAH1B1 | MEGF8 |
| TUBB4B | EEF2 |
| VANGL1 | CTNNB1 |
| YBX1 | GEN1 |
| ANP32A | TNPO2 |
| DEK | BRD2 |
| SLC37A4 | KMT2A |
| TPD52 | CLIC4 |
| CRTC2 | ATIC |
| AMOT | ACTB |
| INTS8 | MBNL1 |
| STK4 | MDH2 |
| DDX46 | YBX3 |
| SIN3A | TTLL12 |
| EEF2 | NOTCH2 |
| STIP1 | TRIM21 |
| LDLR | ELN |
| ACTG1 | PHACTR2 |
| ATXN3 | TUBB |
| STAT1 | NAB1 |
| TULP4 | TMEM65 |
| RPL23 | YBX1 |
| ZNF207 | XRN1 |
| NF1 | IKZF1 |
| ROBO1 | EPHB4 |
| GAPDH | CX3CL1 |
| ATP6V0A2 | GAPDH |
| KDM5A | KMT2D |
| ARF1 | MCOLN3 |
| MYBBP1A | JUN |
| CAMK2B | ACTN4 |
| BUB3 | MSH2 |
| BCOR | SMC1A |
| WEE1 | PARP1 |
| BUB3 | TP53 |
| PTGIS | HOXA13 |
| NF1 | ARID1B |
| MGRN1 | GAPDH |
| MDH2 | DHTKD1 |
| FGFR2 | RRM1 |
| BUB3 | CDK6 |
| TAOK1 | ZBTB47 |
| VEGFA | DROSHA |
| SBNO2 | ST6GALNAC1 |
| CD9 | XRN1 |
| EEF1D | RTN4 |
| POU2F1 | ARID5B |
| TMCO1 | DDX39A |
| IRS4 | ACSL4 |
| POU2F1 | MAZ |
| CDK15 | WEE1 |
| MDM4 | ZNF609 |
| NUP160 | TAF2 |
| ZNF740 | PPP2R3A |
| PHACTR2 | IMPAD1 |
| ACTG1 | CDK2 |
| VPS35 | VAMP7 |
| RPL14 | CTPS1 |
| ACTG1 | DAAM2 |
| BRD2 | GAPDH |
| DICER1 | HNRNPA2B1 |
| DAAM2 | ACTC1 |
| ACTG1 | STAT1 |
| RBFOX2 | ILF3 |
| MAPK8IP2 | DUSP16 |
| NBR1 | DSC3 |
| SULF2 | SDC2 |
| ATXN3 | APP |
| KPNA6 | RBM25 |
| ARID1A | NOTCH2 |
| DCAF7 | ACIN1 |
| SF3B2 | ZCCHC9 |
| PPP1R10 | RUNX1 |
| ROBO1 | PTPRF |
| TSC22D3 | TP53 |
| VPS35 | XRN1 |
| TSC22D3 | SIX1 |
| HOXB2 | HOXB9 |
| ALYREF | NAA50 |
| PHC2 | ZBTB47 |
| TM9SF2 | TTC9 |
| BCOR | MLLT1 |
| DEK | HNRNPA2B1 |
| XRN2 | LIN28B |
| DICER1 | GUF1 |
| ZFP14 | STK38 |
| EEF1D | NAA50 |
| GUF1 | KDM6B |
| AKT2 | PIK3C3 |
| RPS6KA2 | GRB2 |
| MASTL | TPX2 |
| DROSHA | GUF1 |
| RNF4 | ZNF451 |
| NCAPD2 | ADNP2 |
| TPX2 | MSH2 |
| DROSHA | IGF2BP1 |
| CNKSR3 | MANEA |
| FBRS | CPD |
| ACTB | SRCAP |
| ACTB | HNRNPU |
| KPNA6 | NCBP2 |
| DICER1 | ALYREF |
| NCL | MBNL1 |
| KDM1A | BCL11A |
| DLG1 | APP |
| SPEN | CTNNB1 |
| RPS6KA2 | TP53 |
| GTPBP8 | WBP11 |
| ZFP62 | DIRAS1 |
| SPOP | KMT2D |
| ADAMTS5 | LUM |
| ARF1 | VPS35 |
| JUN | HCFC1 |
| SOD2 | RPL23 |
| PPIA | CPLX1 |
| APP | NME8 |
| VEGFA | ITGA5 |
| FBXO42 | STIP1 |
| UBE2F | UBQLN1 |
| MTPN | CADM1 |
| MYO9B | ACTG1 |
| SKIDA1 | UBTF |
| PHC2 | PRDM2 |
| SON | PDXK |
| KDM5C | CTDSPL |
| ANP32A | NCL |
| HNRNPA2B1 | PPP1CC |
| SIN3A | BCL11A |
| SLC38A7 | SLC7A1 |
| SOD2 | UROD |
| H3F3B | GAPDH |
| BTF3L4 | PANK3 |
| UBQLN1 | EEF2 |
| SNAP91 | NT5E |
| BCL11A | RERE |
| DICER1 | IGF2BP2 |
| RBM20 | MBNL1 |
| RRM1 | CDK6 |
| DMRT2 | TP53 |
| TRIP12 | PI4KB |
| PARD3 | DLG1 |
| IP6K1 | PI4KB |
| KDM5A | PBRM1 |
| TMEM177 | RRM1 |
| HNRNPA2B1 | XRN1 |
| MYO9B | ACTB |
| UROD | GAPDH |
| ARID5B | SMARCC1 |
| SND1 | HNRNPU |
| CTPS1 | EEF2 |
| SNRPB | ILF3 |
| MECP2 | SMARCC1 |
| ACTG1 | SRCAP |
| ACSL4 | AASDH |
| ACTC1 | SRCAP |
| EPHB4 | EXOC2 |
| BCOR | IKZF1 |
| EMC1 | ILF3 |
| SRSF7 | CCNL1 |
| BCL9 | CCND1 |
| EMD | DHX9 |
| SP1 | DMWD |
| CD151 | GAPDH |
| SYNRG | WASL |
| SATB1 | PDLIM7 |
| CDK6 | KDM6B |
| TNK2 | DIAPH2 |
| BDP1 | ABI2 |
| ARID1A | MLLT1 |
| NIPA1 | UROD |
| VEGFA | PARP1 |
| VEGFA | CADM1 |
| SPOP | MYBBP1A |
| GRB2 | NISCH |
| MDC1 | POLE |
| ADIPOR1 | SRRM2 |
| ELN | TP53 |
| SHANK2 | NF1 |
| STARD8 | SDR42E1 |
| DRG1 | AGPAT5 |
| TJP1 | TP53 |
| CCDC28A | PHF23 |
| ANP32A | HNRNPK |
| ZFP14 | TTLL12 |
| NFATC2IP | SMC1A |
| AKT2 | NF1 |
| PTPRF | RTN4 |
